# Antibacterial and Antibiofilm Activity of 8‐Hydroxyquinoline Derivatives Against *Mycobacterium* and *Staphylococcus* Species

**DOI:** 10.1002/cbdv.202501892

**Published:** 2025-08-06

**Authors:** Namrita Lall, Anna‐Mari Kok, Carel B. Oosthuizen, Surjeet Verma, François Chassagne, Phuc H. Vo, Khanh‐Van Ho, Chung‐Ho Lin, Cassandra L. Quave, Danielle Twilley

**Affiliations:** ^1^ Department of Plant and Soil Sciences, Faculty of Natural and Agricultural Sciences University of Pretoria Pretoria Gauteng South Africa; ^2^ School of Natural Resources University of Missouri Columbia Missouri USA; ^3^ College of Pharmacy JSS Academy of Higher Education and Research, Mysuru Bonne Terre Vacoas Mauritius; ^4^ South African International Maritime Institute (SAIMI) Nelson Mandela University Gqeberha South Africa; ^5^ Center for the Study of Human Health Emory University College of Arts and Sciences Atlanta Georgia USA; ^6^ Department of Dermatology Emory University School of Medicine Atlanta Georgia USA

**Keywords:** antibiofilm, antimycobacterial, antistaphylococcal, cytotoxicity, quinolines, structure–activity relationships

## Abstract

A series of 8‐alkoxyquinoline derivatives (**QD‐1–12**) were designed and synthesized on the basis of analogues of 8‐hydroxyquinoline (8‐HQ) (**HQ 1–4**). The compounds were evaluated for biofilm inhibition against *Mycobacterium smegmatis* and *Staphylococcus aureus*, including antibacterial activity against *Mycobacterium tuberculosis*, *M. smegmatis* and *S. aureus*. Cytotoxicity was evaluated against human monocyte (U937) and African green monkey kidney (Vero) cell lines. The 8‐*O*‐prenyl derivative (**QD‐12**) showed a minimum inhibitory concentration (MIC) of 12.5 µM, indicating an approximate 8‐fold increased selectivity for the biofilm phenotype and an increased inhibitory activity against methicillin‐resistant *S. aureus* (MRSA) by up to 2‐fold. 5,7‐Dichloro‐8‐hydroxy‐2‐methylquinoline (**HQ‐2**) showed the highest inhibitory potential with MIC values of 0.1, 1.56, 2.2 and 1.1 µM against *M. tuberculosis*, *M. smegmatis*, methicillin‐sensitive *S. aureus* (MSSA) and MRSA, respectively. The results indicate the importance of the 8‐OH group for antibacterial and antimycobacterial activity. Cytotoxicity revealed low‐to‐moderate toxicity of 8‐HQ (**HQ‐1**). All the compounds, except **HQ‐1**, were tested for the first time for their growth and biofilm inhibitory activity against *Mycobacterium* spp. and *S. aureus*.

## Graphical abstract

Synthesis of 8‐hydroxyquinoline derivatives (QD 1‐12) from different analogues of 8‐hydroxyquinoline (HQ 1‐4). The derivatives and parent compounds were evaluated for antibacterial and antibiofilm activity against *Mycobacterium tuberculosis*, *Mycobacterium smegmatis*, methicillin‐resistant *Staphylococcus aureus* and methicillin‐sensitive *Staphylococcus aureus*. Cytotoxicity was evaluated against human monocytes (U937) and African Green monkey kidney (Vero) cells.

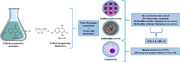

## 1 Introduction

The natural product, 8‐hydroxyquinoline (8‐HQ), is a phytotoxic component found in the roots of *Centaurea diffusa* Lam. [1]. It has shown potent activity against *Mycobacterium tuberculosis* (H37Rv) under replicating and non‐replicating conditions [2]. Its mono‐halogenated derivative, 5‐chloroquin‐8‐ol, has shown more prominent activity against standard strains and drug‐resistant clinical isolates of *M. tuberculosis* with a unique mode of action [3]. A dihalogenated derivative, clioquinol (5‐chloro‐7‐iodo‐quinolin‐8‐ol), was reported to have a synergistic effect, when combined with a sub‐active dose of streptomycin, on *M. tuberculosis*–infected guinea pigs [4]. To date, more than two hundred 8‐HQ derivatives have been evaluated for their antitubercular potential [5, 6]. Furthermore, derivatization of some natural products has been shown to result in increased biological activity [7]. Recently, a quinolone‐based molecule, bedaquiline, was approved by the US Food and Drug Administration (FDA) and the European Medicine Agency as a drug for the treatment of drug‐resistant TB [8]. Bedaquiline is a novel mycobacteria‐specific adenosine triphosphate (ATP) synthase inhibitor [9]. The dihalogenated 8‐HQs (quinolin‐8‐ols), including broxyquinoline, clioquinol, chlorquinaldol and iodoquinol, were widely used to treat intestinal infection during the mid‐19th century [3]. In addition, hydroxyquinolines have been used for the treatment of viral and protozoal infections [10]. Clioquinol, an antifungal and antiprotozoal drug, has also been investigated in a Phase II clinical trial for the treatment of Alzheimer's disease [11]. Furthermore, several 8‐HQ derivatives have shown activity against sensitive and resistant strains of bacteria and their associated persistent biofilms [12, 13, 14, 15].

Bacterial biofilms are intrinsically resistant to conventional antibiotics and are a leading cause of microbial survival, tolerance and antibiotic resistance. Biofilms are complex structures of sessile communities of microorganisms attached to biotic or abiotic surfaces and can be free‐floating. In most biofilms, microbial cells are embedded in self‐synthesized extracellular matrices, composed of polymeric substances (EPSs), such as polysaccharides, proteins, lipids and extracellular DNA (e‐DNA), as well as molecules originating from the host, including mucus and DNA. Microbial cells in biofilms are nearly one thousand times more tolerant to antibiotic therapies than their planktonic counterparts [16, 17]. Considerable research efforts have been made towards the development of new and non‐conventional antimicrobial agents suitable for the treatment of virulence factors and biofilm‐associated infections. A significant, and an often overlooked, contributor to infectious disease, including tuberculosis and *Staphylococcus* infections, is the ability of the bacteria to form biofilms within the host, providing a protective niche that confers tolerance to conventional antibiotic drugs and host immune responses [18, 19]. The presence of these highly resilient biofilms leads to longer treatment periods, increased rates of treatment failure and relapse, and the emergence of drug‐resistance, thereby exacerbating the disease burden and necessitating the urgent discovery of novel antibiofilm agents to improve therapeutic outcomes [20, 21, 22].

Numerous compounds have shown interesting activities in the prophylaxis and treatment of biofilm‐related diseases [23]. Quinoline‐based compounds are one of the noteworthy classes of promising antibiofilm compounds. Recently, several halogenated hydroxyquinolines and derivatives have shown promising antibiofilm activity against several bacteria, either by inhibiting biofilm formation or by dispersing pre‐formed biofilms [13, 24, 25]. Although 8‐alkoxyquinolines were reported to have poor antibacterial activity against *Staphylococcus aureus*, several acyl derivatives of 8‐HQs have shown prominent biofilm dispersion activity [13]. No such structure–activity relationship in 8‐HQs has been reported against tuberculosis; however, a few studies have shown that increasing the lipophilicity of compounds resulted in enhanced antitubercular activity [26, 27]. Therefore, the design of the 8‐HQ derivatives (**QD‐1–12**) was driven by the hypothesis that increasing the lipophilicity through alkylation of the C8‐hydroxyl group would enhance their antimycobacterial, antistaphylococcal and/or antibiofilm activity.

In this study, the aim was to investigate the inhibitory potential of newly designed and synthesized 8‐alkoxyquinoline derivatives for their antibiofilm and antibacterial activity against mycobacterial and staphylococcal species and to assess their structure–activity relationships. Furthermore, human monocytes (U937) and African green monkey kidney cells (Vero) were used to determine the potential cytotoxicity of the derivatives. These findings may highlight the potential for further development of this scaffold in a formal hit assessment or hit‐to‐lead and lead‐optimization phase in a drug discovery programme.

## 2 Results and Discussion

A total of 12 8‐alkoxyquinolines were synthesized on the basis of four parent 8‐HQ compounds. The synthesis was achieved by stirring quinoline with corresponding alkyl bromides in the presence of K_2_CO_3_ (prepared in dimethylformamide [DMF]) (Scheme 1). The reaction products in Entries 10 and 11 were pentyl 8‐(pentyloxy)quinoline‐2‐carboxylate (**QD‐10**) and isopentyl 8‐(isopentyloxy)quinoline‐2‐carboxylate (**QD‐11**), respectively, instead of 8‐(pentyloxy)quinoline‐2‐carbaldehyde and 8‐(isopentyloxy)quinoline‐2‐carbaldehyde, respectively (Scheme 2). The spectroscopic data are available in Figures S3–S54.

**SCHEME 1:**
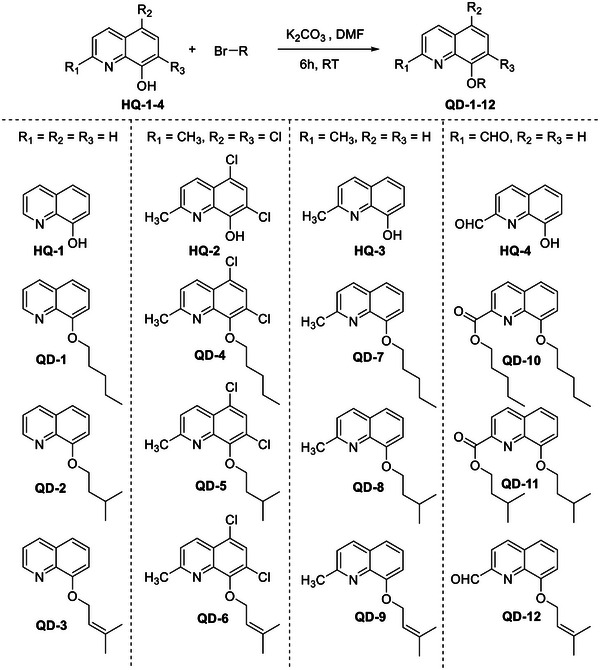
Reaction scheme for the synthesis of **QD‐1–12**.

**SCHEME 2:**
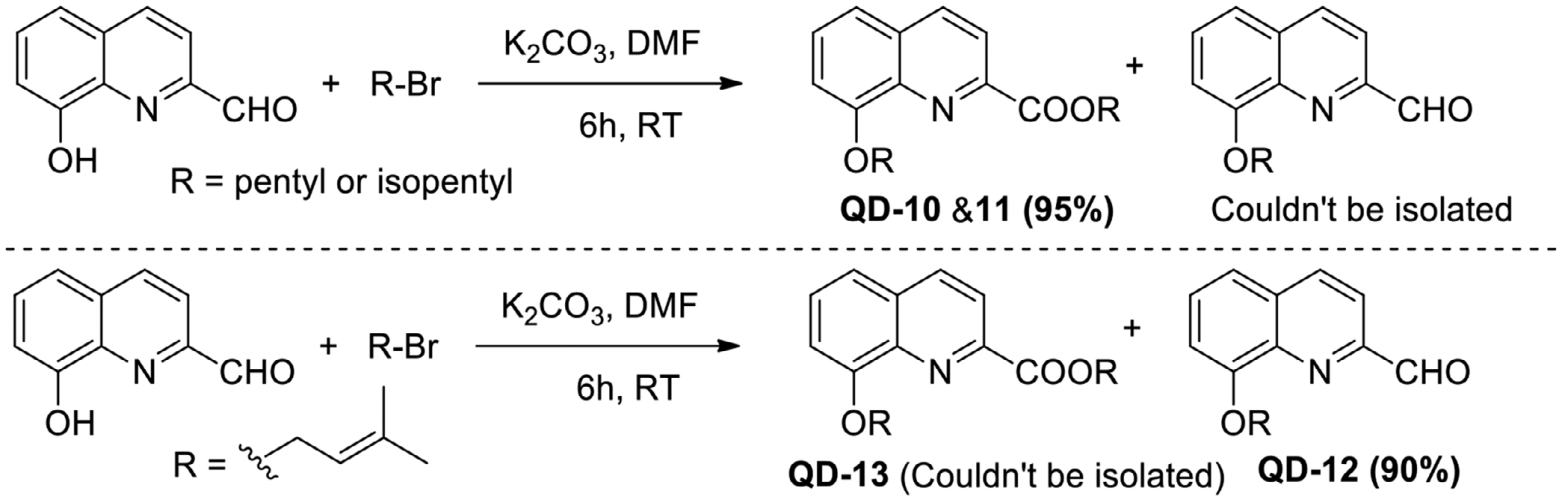
Reaction and products in Entries 10–12. DMF, dimethylformamide.

**SCHEME 3:**
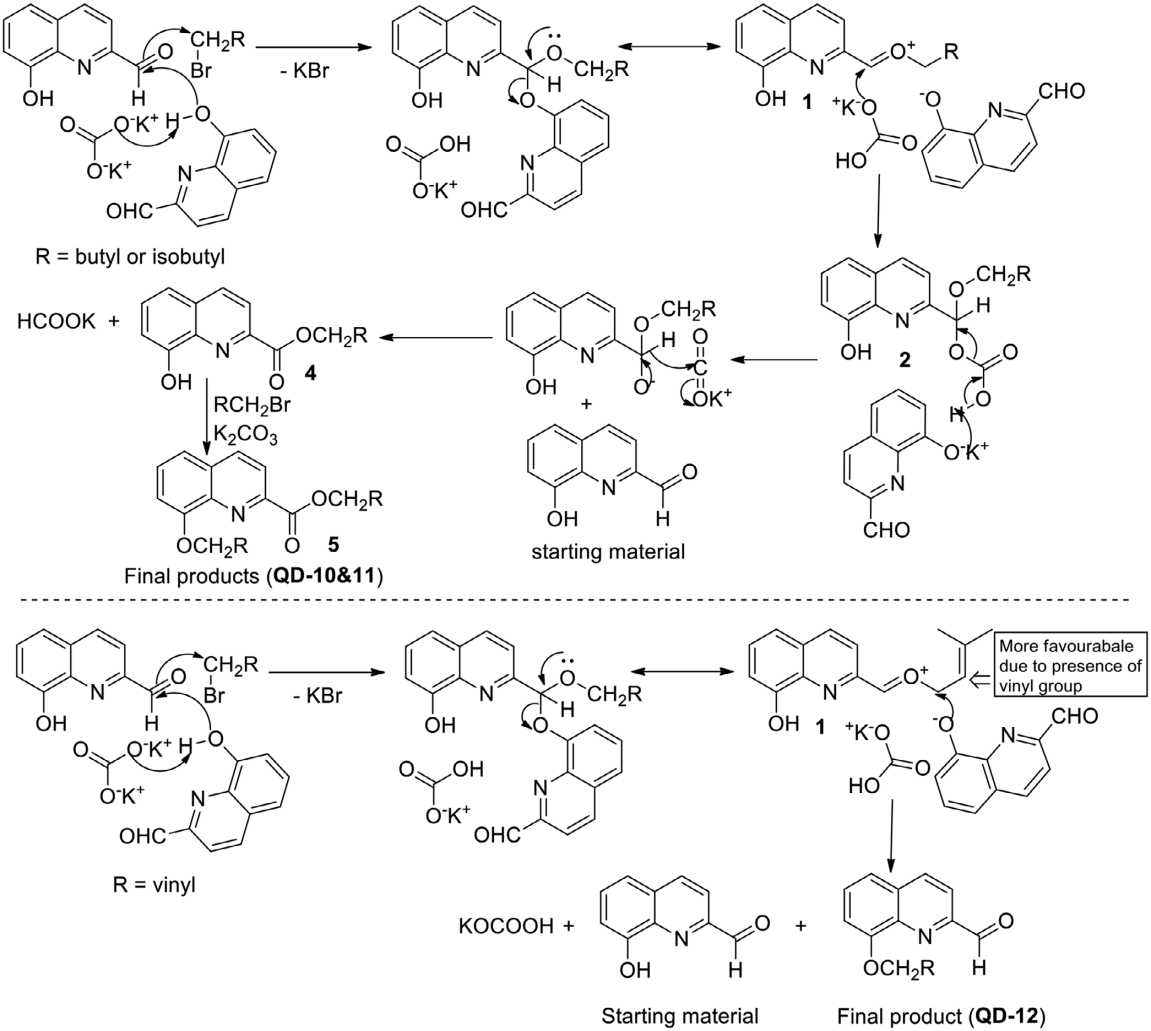
Proposed reaction mechanism involved in the formation of **QD‐10–13**.

The formation of these unexpected products could be explained by the mechanism proposed in Scheme 3. The first step involved the mutual reaction of a 2‐formylquinolin‐8‐olate molecule to produce 2‐(((2‐formylquinolin‐8‐yl)oxy)oxidomethyl)quinolin‐8‐olate and concerted alkylation at oxidomethyl group to produce 2‐(alkoxy((2‐formylquinolin‐8‐yl)oxy)methyl)quinolin‐8‐olate (**1**). The carbonate ion then attacks the highly electrophilic intermediate product (**1**) to produce (8‐hydroxyquinolin‐2‐yl)(alkoxy)methyl hydrogen carbonate (**2**), which undergoes concerted decarboxylation and hydride transfer to release CO_2_ molecule to produce alkyl 8‐hydroxyquinoline‐2‐carboxylate (**4**) and formate ion.

The final step involved alkylation of 8‐hydroxy group in **4** to produce alkyl 8‐(alkoxy)quinoline‐2‐carboxylate (**5**, **QD‐10&11**). The 8‐(alkoxy)quinoline‐2‐carbaldehydes could not be identified in this reaction, which indicated that the carbonate ion attack (at aldehyde group) is preferred over aryloxide ion (at alkyl group). However, in Entry 12, 8‐(alkoxy)quinoline‐2‐carbaldehyde (**QD‐12**) was the major product (90%) (Scheme 2). The corresponding ester (**QD‐13**) could not be isolated. This can be explained by the higher affinity of the prenyl group for the nucleophile than the saturated alkyl group and the preferred attack by aryloxide ion at the prenyl group over the carbonate attack at aldehyde group, which preferably gives Product 3 (Scheme 3).

**SCHEME 4:**
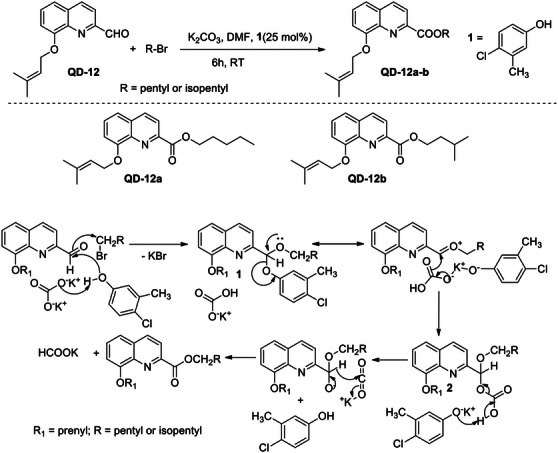
Synthetic scheme for **QD‐12a** and **b** and proposed mechanism.

To confirm the proposed mechanism, **QD‐12** was treated with pentyl and isopentyl bromide under the same reaction condition, and 25 mol% of a phenol (4‐chloro‐3‐methylphenol) was added as a catalyst, which initiated the reaction (Scheme 4). The reaction products (**QD‐12a&b**) were in agreement with the proposed reaction mechanism (Scheme 3), and the products formed were pentyl and isopentyl esters of **QD‐12**, respectively. Further standardization and validation of this synthetic method are in progress.

The quinolone‐based compounds, **HQ‐1**, **HQ‐2**, and **HQ‐3**, showed potent antimycobacterial activity as well as the reduction in *Mycobacterium smegmatis* biofilm formation (Table 1). **HQ‐2** displayed the most prominent antimycobacterial activity with a minimum inhibitory concentration (MIC) of 0.1 µM and a minimum biofilm inhibitory concentration (MBIC) of 3.13 µM. However, there was no indication of a selective inhibition towards biofilm formation (biofilm selective index [BSI] ≤1), and the observed reduction in biofilm is most likely due to the bacterial growth inhibition. Although **QD‐12** showed a moderate selectivity (BSI = 8) towards the inhibition of biofilm formation, this activity could be ascribed to residual inhibition of bacterial growth, and further analysis is needed to assess the specificity. The remaining derivatives showed poor activity against the mycobacterial strains.

**TABLE 1:** Antibacterial and antibiofilm activities and selectivity indexes (SIs) of quinolines and derivatives.

Compounds (molecular weight in g/mol)	*M. tb* MIC (µM)	*M. smeg* MIC (µM)	*M. smeg* MBIC (µM)	*M. smeg* BSI (^MIC^/_MBIC_)	MSSA MIC (µM)	MRSA MIC (µM)	MSSA MBIC (µM)	MRSA MBIC (µM)	MRSA BSI (^MIC^/_MBIC_)
**HQ‐1 (145)**	1	3.13	3.13	1	13.8	6.90	13.80	6.90	1
**HQ‐2 (227)**	0.1	1.56	3.13	0.5	2.2	1.1	17.6	35.2	0.03
**HQ‐3 (159)**	1	6.25	6.25	1	201.23	100.63	100.63	201.23	0.5
**HQ‐4 (173)**	1	>100	>100	—	369.94	369.94	739.88	739.88	0.5
**QD‐1 (215)**	100	>100	>100	—	595.35	595.35	595.35	595.35	1
**QD‐2 (215)**	>100	>100	>100	—	—^a^	1190.70	1190.70	1190.70	1
**QD‐3 (213)**	100	>100	100	1	300.47	300.47	600.94	600.94	0.5
**QD‐4 (297)**	>100	>100	>100	—	—	—	—	—	—
**QD‐5 (297)**	>100	>100	>100	—	—	—	—	—	—
**QD‐6 (295)**	100	>100	>100	—	—	—	—	256.0	—
**QD‐7 (229)**	100	>100	>100	—	1117.90	1117.90	—	—	—
**QD‐8 (229)**	100	>100	100	1	558.95	—	—	—	—
**QD‐9 (227)**	100	>100	>100	—	—	—	—	—	—
**QD‐10 (329)**	100	>100	>100	—	389.06	389.06	—	—	—
**QD‐11 (329)**	100	>100	>100	—	389.06	389.06	—	—	—
**QD‐12 (241)**	100	100	12.5	8	265.56	132.78	531.12	265.56	0.5
**Isoniazid** ^b^	0.3	—	—	—	—	—	—	—	—
**Ciprofloxacin** ^c^	—	0.6	0.325	2	—	—	2.0	1.0	—
**Oxacillin** ^d^	—	—	—	—	1.0	64.0	—	—	—
**Vancomycin** ^e^	—	—	—	—	1.0	1.0	—	—	—

Abbreviations: BSI, biofilm selective index; *M. smeg*, *Mycobacterium smegmatis*; *M. tb*, *Mycobacterium tuberculosis*; MBIC, minimum biofilm inhibitory concentration; MIC, minimum inhibitory concentration.

^a^ Not determined (due to inactivity)

^b^ Positive control against M. tuberculosis.

^c^ Positive control against Mycobacterium smegmatis.

^d^ Positive control against methicillin‐sensitive Staphylococcus aureus.

^e^ Positive control against methicillin‐resistant Staphylococcus aureus.

Two compounds (**HQ‐1** and **HQ‐2**) exhibited noteworthy antibacterial activity against methicillin‐sensitive *S. aureus* (MSSA) UAMS‐1 and methicillin‐resistant *S. aureus* (MRSA) AH1263 or LAC. **HQ‐1** showed an MIC value of 13.8 and 6.9 µM on MSSA and MRSA, respectively, whereas **HQ‐2** showed an MIC value of 2.2 and 1.1 µM on MSSA and MRSA, respectively (Table 1). Both, including **HQ‐4**, showed a higher selectivity towards the resistant versus the susceptible strain with a 2‐fold increase in the antibacterial activity against MRSA.

**HQ‐1** and **HQ‐2** also showed the highest inhibitory activity against biofilm formation of susceptible and resistant *S. aureus* with MBIC values of 6.90 and 35.2 µM, respectively. However, this observed activity is likely due to the potent growth inhibitory effects, rather than true antibiofilm activity. This is evident in the calculated BSI against MRSA, with no biofilm selectivity observed. The alkyl derivatives showed poor activity against bacterial strains and biofilm formation. However, similar to the antimycobacterial activity, the prenyl derivative (**QD‐12**) exhibited increased antibacterial activity compared to the parent compound **HQ‐4**. The staphylococcal growth and biofilm inhibitory curves are available in Figures S1 and S2.

A similar trend to the antibacterial activity was observed when the cytotoxicity of the quinoline compounds was investigated, where the parent compounds (**HQ‐1**, **HQ‐2** and **HQ‐4**) showed significant toxicity when compared to the derivatives, with **HQ‐1**, **HQ‐2** and **HQ‐4** displaying 50% inhibitory concentrating (IC_50_) values <100 µM against the U937 cells and **HQ‐1** and **HQ‐2** showing IC_50_ values <100 µM against the Vero cells (Table 2). Most of the derivatives had IC_50_ values above 100 μ M, indicative of moderate‐to‐low toxicity [28].

**TABLE 2:** Cytotoxicity and selective indexes (SIs) of quinoline derivatives against human monocytes (U937) and monkey kidney (Vero) cells.

Compounds	U937		Vero	
	IC_50_ ^a^ ± SD (µM)	SI	IC_50_ ± SD (µM)	SI
HQ‐1	32.82 ± 11.43	10.5	47.81 ± 6.59	15.3
HQ‐2	2.70 ± 0.31	1.7	82.77 ± 7.09	53.1
HQ‐3	>100	—	>100	—
HQ‐4	4.97 ± 1.93	0.05	>100	—
QD‐1	>100	—	>100	—
QD‐2	>100	—	>100	—
QD‐3	83.83 ± 0.80	0.8	>100	—
QD‐4	>100	—	>100	—
QD‐5	>100	—	>100	—
QD‐6	>100	—	>100	—
QD‐7	>100	—	>100	—
QD‐8	>100	—	>100	—
QD‐9	>100	—	>100	—
QD‐10	69.48 ± 0.16	0.7	84.77 ± 3.42	0.8
QD‐11	>100	—	70.44 ± 3.99	0.7
QD‐12	>100	1	>100	1
Actinomycin D	2.0 × 10^−3^ ± 6.1 × 10^−4^	—	2.0 × 10^−2^ ± 8.0 × 10^−3^	—

Abbreviations: SD, standard deviation; SI, selective index (IC_50_/MIC against *Mycobacterium smegmatis*).

^a^Fifty per cent inhibitory concentration.

From Table 1, it is evident that the 8‐HQs showed an overall increased inhibitory activity against mycobacterial species compared to the staphylococcal bacterial strains. The substitution at the 2‐position did not affect the activity of 8‐HQs against *M. tuberculosis*; however, in the case of **QD‐12**, it reduced the growth and biofilm inhibitory potential of both *M. smegmatis* and *S. aureus*. On the contrary, substitution with halides in ring‐A, significantly improved the antibacterial activity. This is also evident from a previous study conducted against *S. aureus* [12]. Unfortunately, the alkylation of 8‐hydroxy group resulted in a significant reduction in the activity of almost all the 8‐HQs against each of the tested mycobacterial and staphylococcal strains. Alkylation in 8‐hydroxy‐2‐quinoline carboxaldehyde (**HQ‐4**/**QD‐12**) improved the growth inhibitory activity against *M. smegmatis*, MSSA and MRSA by 2‐ to 4‐fold. Enhanced inhibitory activity was observed in *M. smegmatis* biofilm growth with a reduction from >100 to 12.5 µM. Although a promising finding, the observed increased biofilm selectivity could also be explained due to residual bacterial growth inhibition, and further optimization and analysis are required. The structure of the prenyl group at the C8 position of **QD‐12** appears to be important for this biofilm selectivity. Unlike the saturated pentyl or isopentyl groups, the prenyl moiety may have an optimal balance of lipophilicity and molecular shape that enhances specific interactions with components of the biofilm matrix or modulates bacterial quorum‐sensing pathways, rather than directly targeting essential metabolic processes. This suggests a distinct SAR for antibiofilm activity compared to direct antibacterial effects. On the basis of the observations, it may be concluded that the 8‐hydroxy is crucial to increase activity, whereas alkylation of the group may not always produce results that show increased antimycobacterial, antistaphylococcal or antibiofilm activity.

Acylhydroxyquinolines have shown equivalent or better antibacterial activity compared to the corresponding hydroxyquinolines in several previously reported studies [13]. It has been observed, from literature, that increasing the lipophilicity of the molecules generally resulted in increased antimycobacterial activity [26, 27]. Several alkyl hydroxyquinoline/phenazine molecules have also been reported to possess significant antibacterial/antibiofilm activity [24, 25, 29, 30]. Although a reduction in antibacterial activity against *S. aureus* after alkylation of 8‐OH group of a few 8‐HQs has also been reported by Lam et al., there were only a few examples, and therefore, it was difficult to conclude that alkylation at 8‐OH position results in reduced antibacterial activity of diversely substituted quinolines [12]. This study was designed to assess the effect of alkylation of 8‐OH groups in diversely substituted 8‐HQs, resulting in increased lipophilicity of the molecules and may result in increased antistaphylococcal and antimycobacterial activity. However, this hypothesis should be rejected, as most of the synthesized derivatives resulted in the reduction of antimycobacterial and antibacterial activity, whereas only **QD‐12** showed enhanced antibiofilm activity against *M. smegmatis*. It can be concluded that the 8‐OH group is crucial for antibacterial and antimycobacterial activity. This indicates that simple C8‐alkylation, despite increasing lipophilicity, does not typically lead to improved direct antibacterial activity for these quinoline scaffolds; instead, it suggests that the free 8‐OH group is critical for the antibacterial mechanism of action.

In another study, 8‐hydroxy‐2‐quinolinecarbaldehyde has been reported to possess in vitro cytotoxicity against several human cancer cell lines, including breast cancer (MDA‐MB‐231, T‐47D, Hs578t), osteosarcoma (SaoS2), leukaemia (K562), hepatocellular carcinoma (SKHep1) (with a 50% reduction of MTS assay signal compared to the control (MTS_50_) range of 12.5−25 µg/mL) and hepatoma (Hep3B) (with an MTS_50_ of 6.25 ± 0.034 µg/mL). It was observed that the cytotoxicity increased with the alkylation of the 8‐OH group in other quinoline molecules. An increase in the alkyl chain length also resulted in increased cytotoxicity [31]. Thus, on the basis of this observation, it was hypothesized that 8‐alkoxy‐2‐quinolinecarbaldehyde might have more cytotoxic potential than 8‐hydroxy‐2‐quinolinecarbaldehyde. The compounds were evaluated for their cytotoxicity against U937 and Vero cells. Almost all the derivatives were found to have IC_50_ values >100 µM; therefore, they could potentially be considered moderately toxic (if the IC_50_ value was <300 µM); however, they could also be considered to have low toxicity if the IC_50_ values were >300 µM against the tested non‐tumorigenic cell lines. Compounds with IC_50_ <100 µM are considered significantly toxic, as shown with **HQ‐1**, **HQ‐2** and **HQ‐4**. This result confirms that **QD‐12** could potentially be used as an antibiofilm agent against mycobacterial biofilms. However, further analyses through a formal hit assessment and additional toxicity studies are required.

Furthermore, the 8‐alkoxyquinoline‐2‐carboxylates, similar in structure to **QD‐10**, **QD‐11** and **QD‐12**, have been previously reported to have anti‐obesity and mitochondrial uncoupling activities [32]. Thus, the compounds in this study, along with similar compounds, should be considered for evaluation for their anti‐obesity and mitochondrial uncoupling potential. Synthesis of the other quinoline‐2‐carboxylate compounds for the validation of our synthetic method as well as for the evaluation of their anti‐obesity potential is in progress. The synthetic method developed for the synthesis of symmetrical and unsymmetrical 8‐alkoxyquinoline‐2‐carboxylates (**QD‐10**, **QD‐11** and **QD‐12**) is a simple and cost‐effective technique, which produces high yields and involves fewer reaction steps compared to the method followed by Kikuchi et al. [32]. Furthermore, this is the first reported method for the direct conversion of quinolinecarbaldehyde into quinolinecarboxylates using alkyl halide and K_2_CO_3_. Conversion of heterocyclic aldehydes to esters using alcohols has been reported by Goswami et al. [33]. However, this method is limited to the conversion of aldehydes to esters only and cannot be applied for the direct synthesis of alkoxy quinoline carboxylates. Furthermore, this method utilizes hazardous sodium cyanoborohydride (Na(CN)BH_3_) as a catalyst, and the yield is low in comparison to the method described in the current study. Hence, the method described in this study is advantageous over previously reported methods, as it can be applied for the conversion of quinolinecarbaldehydes to corresponding carboxylates and hydroxyquinoline carbaldehydes to alkoxy quinoline carboxylates using mild reaction conditions.

## 3 Conclusions

From Table 1, it is evident that the 8‐HQs showed an overall increased inhibitory activity against mycobacterial species compared to the staphylococcal bacterial strains. The substitution at the 2‐position did not affect the activity of 8‐HQs against *M. tuberculosis*; however, in the case of **QD‐12**, it reduced the growth and biofilm inhibitory potential of both *M. smegmatis* and *S. aureus*. On the contrary, substitution with halides in ring‐A significantly improved the antibacterial activity. This is also evident from a previous study conducted against *S. aureus* [12]. Unfortunately, the alkylation of 8‐hydroxy group resulted in a significant reduction in the activity of almost all the 8‐HQs against each of the tested mycobacterial and staphylococcal strains. Alkylation in 8‐hydroxy‐2‐quinoline carboxaldehyde (**HQ‐4**/**QD‐12**) improved the growth inhibitory activity against *M. smegmatis*, MSSA and MRSA by 2‐ to 4‐fold. Enhanced inhibitory activity was observed in *M. smegmatis* biofilm growth with a reduction from >100 to 12.5 µM. Although a promising finding, the observed increased biofilm selectivity could also be explained due to residual bacterial growth inhibition, and further optimization and analysis are needed. On the basis of the observations, it may be concluded that the 8‐hydroxy is crucial to increase activity, whereas alkylation of the group may not always produce results that show increased antimycobacterial, antistaphylococcal or antibiofilm activity.

## 4 Experimental Section

### 4.1 General

All reagents used in this study were purchased from Sigma‐Aldrich (Johannesburg, South Africa) unless otherwise stated. The NMR spectra were recorded on the Bruker Avance 400 MHz NMR spectrometer using tetramethylsilane (TMS) as an internal standard and CDCl_3_ as a solvent. The HR‐ESIMS data were obtained using a Waters UPLC‐MS system with PDA detector and Waters Synapt G2 QTOF mass spectrometer. The 8‐HQ compounds (**HQ1–4**) were purchased from Sigma Aldrich.

### 4.2 Synthesis

A total of 12 derivatives were synthesized on the basis of the four parent quinoline compounds, namely, 8‐HQ (**HQ‐1**), 5,7‐dichloro‐8‐hydroxy‐2‐methylquinoline (**HQ‐2**), 2‐methyl‐8‐hydroxyquinoline (**HQ‐3**) and 8‐hydroxyquinoline‐2‐carbaldehyde (**HQ‐4**) using three different alkyl bromides (pentyl bromide, isopentyl bromide and prenyl bromide). The quinolines (1 mM) were prepared in 10 mL of dried DMF in a round‐bottom flask. The corresponding alkyl bromide and potassium carbonate (3 mM each) were added to the quinoline solution and stirred for 6 h at room temperature.

The progress of the reaction was monitored by thin layer chromatography (TLC). After the disappearance of the quinoline spot, the reaction was quenched by the addition of distilled water. The reaction mixture was neutralized with 4% HCl solution, and the product was extracted with ethyl acetate. The ethyl acetate layer was washed four times with water to remove residual DMF. Finally, it was washed with a concentrated salt solution, dried over anhydrous sodium sulphate (Na_2_SO_4_) and concentrated under vacuum to yield the crude product. The desired product was purified using column chromatography. The yield of the reactions was between 60% and 95%. Compound structures were confirmed on the basis of NMR and mass spectroscopic data. The synthetic scheme and final products are presented in Scheme 1.

For the full synthetic and characterization details, please review the Supporting Information section.

### 4.3 Antimycobacterial and Biofilm Inhibitory Activity

#### 4.3.1 Mycobacterium Species

The microorganisms, *M. smegmatis* (MC^2^ 155) and a drug‐susceptible strain of *M. tuberculosis*, H37Rv (ATCC 27264), were kindly donated by the South African Medical Research Council, Pretoria, South Africa. Cultures of *M. smegmatis* were maintained on Middlebrook 7H11 agar base plates and allowed to grow for 24 h at 37°C, followed by sub‐culturing for an additional 24 h. *Mycobacterium tuberculosis* was plated onto Löwenstein–Jensen medium and allowed to grow for 3–4 weeks at 37°C.

#### 4.3.2 Antimycobacterial Activity

The antimycobacterial activity of the quinoline compounds and derivatives was tested using the microtitre Alamar Blue assay method, as described by Franzblau et al., with minor modifications with the use of the viability reagent, Presto Blue [34, 35, 36]. Briefly, 100 µL of 7H9 broth (supplemented with 0.4% glycerol, 0.5% Tween 80 and OADC) was dispensed in each well of a sterile flat‐bottom 96‐well plate. The compounds were dissolved to a stock concentration of 20 mM (100% DMSO) and further diluted in media, whereafter 100 µL of the prepared compounds and the positive controls, ciprofloxacin (*M. smegmatis*) and isoniazid (*M. tuberculosis*), were added to the first row of wells, followed by a 2‐fold serial dilution yielding a test concentration range of 0.8–100 µM for the compounds (10‐fold serial dilution for *M. tuberculosis* [0.0001–100 µM] and 0.04–5 µg/mL for ciprofloxacin [0.2–15 µM] and isoniazid [0.3–36.5 µM]). Negative untreated bacteria, sterility and vehicle (1% DMSO) controls were included in the assay. The bacterial inoculum was prepared to a 0.5 McFarland and diluted to yield a test concentration of 1.5 × 10^6^ CFU/mL. The inoculum of 100 mL was added to all the wells, excluding the sterility controls, to yield a final assay volume of 200 µL. After a 24‐h (5 days for *M. tuberculosis*) incubation period at 37°C, 20 µL of Presto Blue (Thermo Fisher Scientific, Massachusetts, USA) was added to all the wells and incubated for an additional 3–4 h. The samples were tested in triplicate in two or more independent experiments. The MIC values were defined as the lowest concentration where no colour change from blue to pink could be observed.

#### 4.3.3 Mycobacterial Biofilm Inhibition

A microtitre antibiofilm assay was conducted in a sterile flat‐bottomed 96‐well plate for the determination of the biofilm inhibitory activity. Briefly, 100 µL of basic 7H9 media (without Tween 80) was added to the plates. The subsequent addition of 100 µL of the samples was added to the first row of the wells, followed by a 2‐fold serial dilution yielding the same concentration range as mentioned above. The *M. smegmatis* inoculum was prepared to a 0.5 McFarland and diluted 100‐fold to yield a test concentration of 1.5 × 10^6^ CFU/mL. Negative untreated bacteria, sterility and vehicle (1% DMSO) controls were included in the assay [37, 38]. The plates were incubated for 2–4 days at 37°C. The MBIC was observed and determined as the lowest concentration at which no visible biofilm formation could be observed. The experiment was repeated in three independent experiments and tested in triplicate.

### 4.4 Antistaphylococcal Biofilm Inhibitory Activity

#### 4.4.1 *Staphylococcus aureus* Strains

To determine the antibacterial activity of the compounds, two *S. aureus* strains were used. An MSSA identified as *S. aureus* UAMS‐1 was provided by Mark Smeltzer, and an MRSA identified as AH1263 or LAC was provided by Alexander Horswill. The same strains used for MIC determination were employed for the MBIC assays. *Staphyllococcus aureus* UAMS‐929, the isogenic sarA mutant of UAMS‐1 (biofilm‐deficient phenotype), was used as a positive control (Table 3).

**TABLE 3:** Description of the *Staphylococcus aureus* bacterial strains used in this study.

Strain ID	Characteristics	Resistance profile	Source	Ref.
UAMS‐1, ATCC 49230	Osteomyelitis clinical isolate; prototype biofilm isolate. Methicillin‐sensitive	BEN	MS	[36]
LAC, AH1263	USA300. Methicillin‐resistant	BEN, CIP, LEV, MET, MOX, OXA, PIP	AH	[37]

*Note*: “MS” denotes M. Smeltzer, UAMS.3. “AH” denotes A. Horswill.

Abbreviations: BEN, benzylpenicillin; CIP, ciprofloxacin; LEV, levofloxacin; MET, methicillin; MOX, moxifloxacin; OXA, oxacillin; PIP, piperacillin.

#### 4.4.2 Antistaphylococcal Activity

A broth microdilution assay in a 96‐well plate was used to determine the MIC as described by the CLSI guidelines [39]. The overnight culture was standardized to 5 × 10^5^ CFU/mL in CAMHB media with a Cytation 3 multimode plate reader (BioTek, Winooski, VT, USA) with optical density OD_600_ nm, as previously described. Vehicle (100% DMSO) and positive controls (antibiotics: oxacillin and vancomycin) were included in each experiment. The final total per cent DMSO in the well volume was <5% for all assays. Compounds were dissolved in DMSO at a stock concentration of 20 mg/mL prior to dispensing in 96‐well plates to reach final test concentrations ranging from 0.125 to 256 µg/mL (approximately 0.5–1000 µM) by serial dilution. Plates were read at 0 h and after 18 h of static incubation with humidity at 37°C. Per cent inhibition of bacterial growth was calculated using the formula: (1 − (ΔOD_sample_/ΔOD_vehicle_)) × 100 [40]. All the compounds were tested in triplicate in two independent experiments.

#### 4.4.3 *Staphylococcal* Biofilm Inhibition

A microtitre test in a 96‐well plate was performed to determine the antibiofilm activity of the quinolone derivatives. Overnight culture was standardized to 5 × 10^5^ CFU/mL in biofilm media (TSB + 3% NaCl + 0.5% dextrose) by using a Cytation 3 multimode plate reader (BioTek, Winooski, VT, USA) with optical density OD_600_ nm, as previously described [41, 42]. Human plasma was added to the media at 2% of the total volume of biofilm media at the time of the experimental setup. Samples were dissolved in DMSO at a stock concentration of 20 mg/mL. The test concentration range was from 0.125 to 256 µg/mL (approximately 0.5–1000 µM) by serial dilution, and a vehicle control was included. Plates were incubated for 22 h of static incubation with humidity at 37°C. Following incubation, the wells were gently washed with phosphate‐buffered saline (PBS), fixed with ethanol, stained with 0.2% crystal violet, rinsed in tap water and the stain eluted into 33% acetic acid and transferred to a new plate prior to quantification of the eluent at OD_595_ nm. All the compounds were tested in triplicate in two independent experiments.

### 4.5 Cytotoxicity

The human monocyte (U937) and African green monkey kidney (Vero) cell lines were used in this study to determine the cytotoxic potential of the quinoline derivatives. The cell lines were purchased from Separation Scientific SA (Pty) Ltd. (Johannesburg, South Africa) and maintained in Roswell Park Memorial Institute (RPMI) 1640 and Eagle's Minimal Essential Medium (EMEM), supplemented with 10% heat‐inactivated foetal bovine serum (FBS), 1% antibiotics (100 U/mL penicillin and 100 µg/mL streptomycin) and 1% antifungal agent (250 µg/mL amphotericin B) as previously described [28]. The cells were cultured at 37°C, 5% CO_2_ in a humidified incubator to 80% confluency. Detachment was achieved by rinsing the cells with phosphate buffer saline followed by the addition of trypsin–EDTA (0.25% trypsin containing 0.01% EDTA). After 5 min, the reaction was inhibited through the addition of supplemented media.

Cell viability was assessed through the method described by Lall et al., using Presto Blue as the viability reagent [35]. The cells were seeded in a flat‐bottom 96‐well plate (100 µL) at a concentration of 1.0 × 10^6^ cells/mL and allowed to adhere for 24 h. The samples were prepared in DMSO at a stock concentration of 20 mM, serially diluted in complete media and added to the plate at a final concentration range of 0.78–100 µM. Controls included a vehicle control (2% DMSO), an untreated cell control, a 0% control (no cells) and actinomycin D, as the positive control, at a final test concentration range of 3.9 × 10^−4^ to 0.05 µg/mL. Cells were incubated for 72 h, followed by the addition of 20 µL of Presto Blue reagent to all the wells and an additional 2 h incubation. Cell viability was measured using a VICTOR Nivo Multimode Microplate Reader (PerkinElmer) at an excitation/emission wavelength of 540/590 nm. The 50% inhibitory concentration (IC_50_) was calculated using GraphPad Prism 4.

### 4.6 Statistical Analysis

All results are presented as the mean ± standard deviation (where applicable) of experiments done in triplicate and in two or three independent experiments (as stated in each methods section). Descriptive analysis was used to directly compare and rank the compounds and derivatives based on their activity in the biological assays. Additionally, by comparing the antibiofilm activity to the antibacterial activity and the antibacterial activity to the cytotoxicity, selectivity indices were used to assess the preferential activity of the compounds. Overall, 50% inhibitory concentrations (absolute IC_50_) were calculated using a four‐parameter logistic equation with constraints on the top (100) and bottom (0) parameters.

## Author Contributions

**Namrita Lall**: conceptualization, funding acquisition, project administration, resources, validation, writing – review and editing, supervision. **Anna‐Mari Kok**: writing – original draft preparation, writing – review and editing. **Carel B. Oosthuizen**: conceptualization, data curation, investigation, methodology, visualization, formal analysis, writing – original draft preparation, writing – review and editing. **Surjeet Verma**: conceptualization, data curation, investigation, methodology, visualization, formal analysis, writing – original draft preparation, writing – review and editing. **François Chassagne**: data curation, investigation, methodology, visualization, formal analysis, writing – review and editing. **Phuc H. Vo**: data curation, investigation, methodology, visualization, formal analysis. **Khanh‐Van Ho**: data curation, investigation, methodology, visualization, formal analysis. **Chung‐Ho Lin**: funding acquisition, project administration, resources, validation, writing – review and editing, supervision. **Cassandra L. Quave**: funding acquisition, project administration, resources, validation, writing – review and editing, supervision. **Danielle Twilley**: data curation, investigation, methodology, visualization, formal analysis, writing – original draft preparation, writing – review and editing. All authors have read and agreed to the published version of the manuscript.

## Conflicts of Interest

The authors declare no conflicts of interest.

## Supporting information




**Supporting File 1**: cbdv70329‐sup‐0001‐SuppMat.docx

## Data Availability

The data that support the findings of this study are available from the corresponding author upon reasonable request.
