# Supplementary material for: Antibacterial and Antibiofilm Activity of 8‐Hydroxyquinoline Derivatives Against Mycobacterium and Staphylococcus Species
Source: Chem Biodivers. 2025 Aug 6;22(12):e01892. doi: 10.1002/cbdv.202501892 (PMC12716008; doi:10.1002/cbdv.202501892)
Supplement: Supplementary file 1 — Supporting File 1: cbdv70329‐sup‐0001‐SuppMat.docx [file CBDV-22-e01892-s001.pdf]

Supplementary Material

Antibacterial and antibiofilm activity of 8-hydroxyquinoline derivatives against *Mycobacterium* and *Staphylococcus* species

Table of contents

1. General Experimental Procedure 1
2. Chemical synthesis 2
3. Antistaphylococcal activity 5
4. Spectroscopic data 7

1. General Experimental Procedure

The NMR spectra were recorded on the Brucker Avance, 400MHz NMR spectrometer using tetramethylsilane (TMS) as an internal standard $CDCl_3$ as solvent. The HR-ESIMS data were obtained using a Waters UPLC-MS system with PDA detector and Waters Synapt G2 QTOF mass spectrometer. The 8-hydroxyquinolines (HQ1-4) were purchased from Sigma Aldrich, South Africa.

2. Chemical Synthesis

The quinolines (1 mM) were taken into 10 mL of dried dimethyl formamide (DMF) in a round bottom flask. The corresponding alkyl bromide and potassium corbonate (3 mM each) were added to the quinoline solution and stirred for 6 h at room temperature. The progress of the reaction was monitored by TLC. After disappearance of the quinoline spot, the reaction was quenched by the addition of distilled water. The reaction mixture was then neutralized with 4% HCl solution and the product was extracted with ethyl acetate. The ethyl acetate layer was washed 4 times with water to remove the DMF. Finally, it was washed with brine, dried over anhydrous sodium sulfate (Na_2SO_4) and concentrated under vacuum to yield the crude product. The desired product was purified by using column chromatography. Yield of the reactions were between 60-95%. Compound structures were confirmed based on NMR and mass spectroscopic data.

2.1. 8-(Pentyloxy)quinoline (QD-1):

Yield: 91%; **^{1}H NMR ($CDCl_3$, 400 MHz):** δ_H, 0.91 (3H, t, *J* = 7.2 Hz), 1.40 (2H, m), 1.48 (2H, m), 2.00 (2H, m), 4.18 (2H, t, *J* = 7.0 Hz), 7.00 (1H, d, *J* = 7.7 Hz), 7.30-7.42 (3H, dd, *J* = 2.0, 2.2, 3.9, 4.1, 7.9, 8.2 Hz), 8.00 (1H, dd, *J* = 1.7, 8.3 Hz), 8.92 (1H, dd, *J* = 1.7, 4.2 Hz). **^{13}C NMR ($CDCl_3$, 100 MHz):** δ_C, 14.03 (CH_3), 22.53 (CH_2), 28.20 (CH_2), 28.68 (CH_2), 68.95 (CH_2), 108.57 (CH), 119.27 (CH), 121.52 (CH), 126.74 (CH), 129.48 (C), 136.04 (CH), 140.16 (C), 149.12 (CH), 154.79 (C). HRESI-MS: m/z, 216.1403 $[M+H]^+$ (Calcd; 216.1388 for $C_{14}H_{18}NO$).

2.2. 8-(Isopentyloxy)quinoline (QD-2):

Yield: 93%; **^{1}H NMR ($CDCl_3$, 400 MHz):** δ_H, 0.90 (6H, d, *J* = 5.8 Hz), 1.17 (1H, br s), 1.84 (2H, m), 4.17 (2H, t, *J* = 6.1 Hz), 6.95 (1H, br d, *J* = 7.6 Hz), 7.25-7.36 (3H, dd, *J* = 3.8, 4.1, 8.2 Hz), 8.00 (1H, dd, *J* = 1.7, 8.3), 8.85 (1H, dd, *J* = 1.7, 4.2). **^{13}C NMR ($CDCl_3$, 100 MHz):** δ_C, 22.79 (2 x CH_3), 25.35 (CH), 37.73 (CH_2), 67.52 (CH_2), 108.65 (CH), 119.42 (CH), 121.59 (CH), 126.78 (CH), 129.58 (C), 135.99 (CH), 140.47 (C), 149.32 (CH), 154.97 (C). HRESI-MS: m/z, 216.1395 $[M+H]^+$ (Calcd; 216.1388 for $C_{14}H_{18}NO$).

2.3. 8-((3-Methylbut-2-en-1-yl)oxy)quinoline (QD-3):

Yield: 84%; **^{1}H NMR (CDCl$_3$, 400 MHz):** δ_H, 1.703-1.707 (6H, s), 4.73 (2H, d, *J* = 6.6 Hz), 5.59 (1H, dt, *J* = 1.1, 6.6 Hz), 6.96 (1H, br d, *J* = 7.6 Hz), 7.27-7.37 (3H, dd, *J* = 1.5, 4.1, 7.7, 8.2 Hz), 8.01 (1H, br d, *J* = 8.3 Hz), 8.86 (1H, dd, *J* = 1.9, 2.2 Hz). **^{13}C NMR (CDCl$_3$, 100 MHz):** δ_C, 18.44 (CH_3), 25.97 (CH_3), 65.85 (CH_2), 108.89 (CH), 119.48 (CH), 120.06 (CH), 121.66 (CH), 126.78 (CH), 129.58 (CH), 136.00 (CH), 137.59 (C), 140.54 (C), 149.38 (CH), 154.73 (C). HRESI-MS: m/z, 214.1236 $[M+H]^+$ (Calcd; 214.1232 for $C_{14}H_{16}NO$).

2.4. 5,7-Dichloro-2-methyl-8-(pentyloxy)quinoline (QD-4):

Yield: 95%; **^{1}H NMR (CDCl$_3$, 400 MHz):** δ_H, 0.94 (3H, t, *J* = 7.3 Hz), 1.41 (2H, m), 1.55 (2H, m), 1.92 (2H, m), 2.76 (3H, s), 4.34 (2H, t, *J* = 6.7 Hz), 7.33 (1H, br d, *J* = 8.7 Hz), 7.53 (1H, br s), 8.33 (1H, br d, *J* = 8.6 Hz). **^{13}C NMR (CDCl$_3$, 100 MHz):** δ_C, 14.27 (CH_3), 22.68 (CH_2), 25.70 (CH_3), 28.29 (CH_2), 30.14 (CH_2), 75.57 (CH_2), 122.92 (CH), 124.67 (C), 125.82 (C), 126.76 (C), 126.98 (CH), 133.38 (CH), 143.59 (C), 150.70 (C), 159.97 (C). HRESI-MS: m/z, 298.0766 $[M+H]^+$ (Calcd; 298.0765 for $C_{15}H_{18}Cl_2NO$).

2.5. 5,7-Dichloro-8-(isopentyloxy)-2-methylquinoline (QD-5):

Yield: 89%; **^{1}H NMR (CDCl$_3$, 400 MHz):** δ_H, 0.96 (6H, d, *J* = 6.6 Hz), 1.78 (2H, m), 2.00 (1H, m), 2.73 (3H, s), 4.34 (2H, t, *J* = 6.7 Hz), 7.31 (1H, d, *J* = 8.7 Hz), 7.51 (1H, s), 8.30 (1H, d, *J* = 6.6 Hz). **^{13}C NMR (CDCl$_3$, 100 MHz):** δ_C, 22.85 (2 x CH_3), 24.98 (CH_3), 25.70 (CH), 39.26 (CH_2), 73.95 (CH_2), 122.97 (CH), 124.71 (C), 125.89 (C), 126.84 (C), 127.00 (CH), 133.44 (CH), 143.61 (C), 150.71 (C), 160.03 (C). HRESI-MS: m/z, 298.0767 $[M+H]^+$ (Calcd; 298.0765 for $C_{15}H_{18}Cl_2NO$).

2.6. 5,7-Dichloro-2-methyl-8-((3-methylbut-2-en-1-yl)oxy)quinoline (QD-6):

Yield: 82%; **^{1}H NMR (CDCl$_3$, 400 MHz):** δ_H, 1.70 (3H, s), 1.75 (3H, s), 2.76 (3H, s), 4.90 (2H, d, *J* = 7.4 Hz), 5.68 (1H, t, *J* = 7.4 Hz), 7.34 (1H, br d, *J* = 8.7 Hz), 7.51 (1H, br s), 8.32 (1H, br d, *J* = 8.6 Hz). **^{13}C NMR (CDCl$_3$, 100 MHz):** δ_C, 18.20 (CH_3), 25.64 (CH_3), 25.99 (CH_3), 71.47 (CH_2), 120.33 (CH), 122.89 (CH), 124.58 (C), 125.92 (C), 126.89 (CH), 127.18 (C), 133.38 (CH), 139.40 (C), 143.69 (C), 150.11 (C), 159.91 (C). HRESI-MS: Molecular ion peak could not be found probably due to instability. The base peak was observed at m/z 227.9985 (Calcd; 227.9983 for $C_{10}H_8Cl_2NO$).

2.7. 2-Methyl-8-(pentyloxy)quinoline (QD-7):

Yield: 78%; **^{1}H NMR (CDCl$_3$, 400 MHz):** δ_H, 0.87 (3H, t, *J* = 7.2 Hz), 1.31-1.44 (4H, m), 1.94 (2H, m), 2.69 (3H, s), 4.12 (2H, t, *J* = 7.1 Hz), 6.94 (1H, d, *J* = 7.6 Hz), 7.19-7.31 (3H, d, *J* = 8.0, 10.2, 11.0 Hz), 7.91 (1H, d, *J* = 8.6 Hz). **^{13}C NMR (CDCl$_3$, 100 MHz):** δ_C, 14.22 (CH_3), 22.72 (CH_2), 25.36 (CH_3), 28.31 (CH_2), 28.71 (CH_2), 69.21 (CH_2), 108.13 (CH), 119.35 (CH), 122.79 (CH), 126.01 (CH), 127.91 (C), 136.61 (CH), 139.62 (C), 154.33 (C), 158.20 (C). HRESI-MS: m/z, 230.1546 $[M+H]^+$ (Calcd; 230.1545 for $C_{15}H_{20}NO$).

2.8. 8-(Isopentyloxy)-2-methylquinoline (QD-8):

Yield: 74%; **^{1}H NMR (CDCl$_3$, 400 MHz):** δ_H, 0.93 (6H, d, *J* = 6.4 Hz), 1.78-1.89 (3H, m), 2.71 (3H, s), 4.19 (2H, t, *J* = 7.1 Hz), 6.97 (1H, dd, *J* = 1.3, 7.5 Hz), 7.19-7.33 (3H, dd, *J* = 1.3, 8.0, 8.2, 8.4 Hz), 7.93 (1H, d, *J* = 8.4 Hz). **^{13}C NMR (CDCl$_3$, 100 MHz):** δ_C, 22.97 (2 x CH_3), 25.59 (CH_3), 25.77 (CH), 37.69 (CH_2), 67.86 (CH_2), 109.21 (CH), 119.44 (CH), 122.73 (CH), 126.00 (CH), 127.92 (C), 136.51 (CH), 139.86 (C), 154.46 (C), 158.26 (C). HRESI-MS: m/z, 230.1551 $[M+H]^+$ (Calcd; 230.1545 for $C_{15}H_{20}NO$).

2.9. 2-Methyl-8-((3-methylbut-2-en-1-yl)oxy)quinoline (QD-9):

Yield: 60%; **^{1}H NMR ($CDCl_3$, 400 MHz):** δ_H, 1.70 (3H, s), 1.75 (3H, s), 2.76 (3H, s), 4.91 (2H, t, *J* = 7.1 Hz), 5.68 (1H, t, *J* = 7.4 Hz), 6.97 (1H, dd, *J* = 1.3, 7.5 Hz), 7.19-7.33 (3H, dd, *J* = 1.4, 7.2, 8.1, 8.4 Hz), 7.93 (1H, d, *J* = 8.4 Hz). **^{13}C NMR ($CDCl_3$, 100 MHz):** δ_C, 18.44 (CH_3), 25.64 (CH_3), 25.99 (CH_3), 71.47 (CH_2), 109.21 (CH), 119.44 (CH), 120.33 (CH), 122.73 (CH), 126.00 (CH), 127.92 (C), 136.51 (CH), 139.86 (C), 154.46 (C), 158.26 (C). HRESI-MS: Molecular ion peak was absent.

2.10. Pentyl 8-(pentyloxy)quinoline-2-carboxylate (QD-10):

Yield: 95%; **^{1}H NMR ($CDCl_3$, 400 MHz):** δ_H, 0.90 (3H, t, *J* = 7.1 Hz), 0.92 (3H, t, *J* = 7.3 Hz), 1.34-1.56 (8H, m), 1.84 (2H, m), 2.03 (2H, m), 4.17 (2H, t, *J* = 7.0 Hz), 4.39 (2H, t, *J* = 6.9 Hz), 7.03 (1H, d, *J* = 7.8 Hz), 7.36 (1H, br d, *J* = 7.6 Hz), 7.49 (1H, dt, *J* = 1.3, 7.8 Hz), 8.14 (1H, dd, *J* = 1.0, 8.5 Hz), 8.19 (1H, dd, *J* = 1.1, 8.6 Hz). **^{13}C NMR ($CDCl_3$, 100 MHz):** δ_C, 14.16 (CH_3), 14.23 (CH_3), 22.57 (CH_2), 22.72 (CH_2), 28.30 (CH_2), 28.35 (CH_2), 28.47 (CH_2), 28.81 (CH_2), 66.32 (CH_2), 69.35 (CH_2), 109.34 (CH), 119.10 (CH), 121.58 (CH), 129.26 (CH), 130.71 (C), 137.18 (CH), 139.93 (C), 147.07 (C), 155.88 (C), 165.85 (C). HRESI-MS: m/z, 330.2076 $[M+H]^+$ (Calcd; 330.2069 for $C_{20}H_{28}NO_3$).

2.11. Isopentyl 8-(isopentyloxy)quinoline-2-carboxylate (QD-11):

Yield: 95%; **^{1}H NMR ($CDCl_3$, 400 MHz):** δ_H, 0.95 (6H, dd, *J* = 0.7, 6.5 Hz), 0.98 (6H, d, *J* = 5.6 Hz), 1.71-1.93 (6H, m), 4.20 (2H, t, *J* = 6.0 Hz), 4.43 (2H, t, *J* = 6.9 Hz), 7.03 (1H, d, *J* = 6.1 Hz), 7.35 (1H, d, *J* = 8.3 Hz), 7.49 (1H, dt, *J* = 1.1, 8.0 Hz), 8.12 (1H, dd, *J* = 0.8, 8.5 Hz), 8.18 (1H, dd, *J* = 1.0, 8.5 Hz). **^{13}C NMR ($CDCl_3$, 100 MHz):** δ_C, 22.72 (CH_3), 22.89 (CH_3), 25.33 (CH), 25.46 (CH), 37.40 (CH_2), 37.73 (CH_2), 64.78 (CH_2), 67.78 (CH_2), 109.28 (CH), 119.06 (CH), 121.53 (CH), 129.22 (CH), 130.66 (C), 137.11 (CH), 139.93 (C), 147.00 (C), 155.90 (C), 165.83 (C). HRESI-MS: m/z, 330.2079 $[M+H]^+$ (Calcd; 330.2069 for $C_{20}H_{28}NO_3$).

2.12. 8-((3-Methylbut-2-en-1-yl)oxy)quinoline-2-carbaldehyde (QD-12):

Yield: 90%; **^{1}H NMR ($CDCl_3$, 400 MHz):** δ_H, 1.77 (6H, s), 4.84 (2H, d, *J* = 6.5 Hz), 5.63 (1H, dt, *J* = 1.3, 6.5 Hz), 7.10 (1H, d, *J* = 7.8 Hz), 7.40 (1H, td, *J* = 0.9, 8.2 Hz), 7.54 (1H, dt, *J* = 1.6, 8.0 Hz), 8.0 (1H, dd, *J* = 1.8, 8.4 Hz), 8.22 (1H, dd, *J* = 2.5, 8.4 Hz), 10.26 (1H, t, *J* = 0.8 Hz). **^{13}C NMR ($CDCl_3$, 100 MHz):** δ_C, 18.59 (CH_3), 26.03 (CH_3), 66.44 (CH_2), 110.10 (CH), 117.96 (CH), 119.58 (CH), 119.64 (CH), 129.93 (CH), 131.56 (C), 137.43 (CH), 138.12 (C), 140.33 (C), 151.54 (C), 155.50 (C), 194.06 (CH). HRESI-MS: m/z, 242.1186 $[M+H]^+$ (Calcd; 242.1181 for $C_{15}H_{16}NO_2$).

2.13. Pentyl 8-((3-methylbut-2-en-1-yl)oxy)quinoline-2-carboxylate (QD-12a):

Yield: 92%; **^{1}H NMR ($CDCl_3$, 400 MHz):** δ_H, 0.91 (3H, t, *J* = 7.12 Hz), 1.35-1.46 (4H, m), 1.76 (3H, s), 1.78 (3H, s), 1.85 (2H, m), 4.41 (2H, t, *J* = 7.00 Hz), 4.80 (2H, d, *J* = 6.4 Hz), 5.63 (1H, tt, *J* = 1.3, 6.5 Hz), 7.05 (1H, d, *J* = 7.2 Hz), 7.37 (1H, dd, *J* = 0.72, 8.2 Hz), 7.51 (1H, t, *J* = 8.0 Hz), 8.13 (1H, d, *J* = 8.48 Hz), 8.20 (1H, d, *J* = 8.5 Hz). **^{13}C NMR ($CDCl_3$, 100 MHz):** δ_C, 14.17 (CH_3), 18.56 (CH_3), 22.58 (CH_2), 25.99 (CH_3), 66.35(CH_2), 66.40(CH_2), 109.77 (CH), 119.19 (CH), 120.00 (CH), 121.62 (CH), 129.20 (CH), 130.73 (C), 137.20 (CH), 137.44 (C), 140.08 (C), 147.15 (C), 155.62 (C), 165.82 (C). HRESI-MS: m/z, 328.1909 $[M+H]^+$ (Calcd; 328.1913 for $C_{20}H_{26}NO_3$).

2.14. Isopentyl 8-((3-methylbut-2-en-1-yl)oxy)quinoline-2-carboxylate (QD-12b):

Yield: 89%; **^{1}H NMR ($CDCl_3$, 400 MHz):** δ_H, 0.96 (6H, d, *J* = 6.4 Hz), 1.76 (6H, s), 1.75-1.78 (3H, m), 4.44 (2H, t, *J* = 7.00 Hz), 4.79 (2H, d, *J* = 6.4 Hz), 5.66 (1H, tt, *J* = 1.4, 6.5 Hz), 7.05 (1H, d, *J* = 7.8 Hz), 7.37 (1H, d, *J* = 8.4 Hz), 7.50 (1H, t, *J* = 8.2 Hz), 8.14 (1H, d, *J* = 8.5 Hz), 8.20 (1H, d, *J* = 8.5 Hz). **^{13}C NMR ($CDCl_3$, 100 MHz):** δ_C, 18.55 (CH_3), 22.75 (2 x CH_3), 25.43 (CH), 25.99 (CH_3), 37.40 (CH_2), 64.97 (CH_2), 66.31(CH_2), 109.76 (CH), 119.17 (CH), 119.98

(CH), 121.58 (CH), 129.20 (CH), 130.73 (C), 137.19 (CH), 137.46 (C), 140.07 (C), 147.10 (C), 155.61 (C), 165.80 (C). HRESI-MS: m/z, 328.1913 $[M+H]^+$ (Calcd; 328.1913 for $C_{20}H_{26}NO_3$).

3. Antistaphylococcal activity

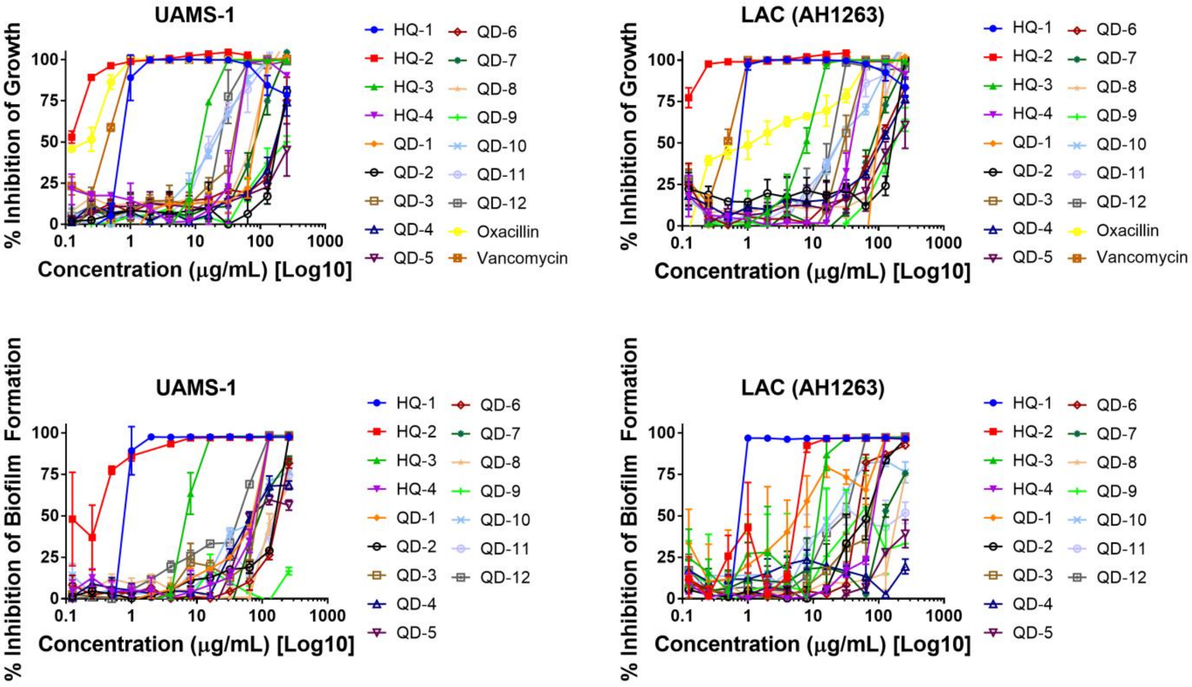

Figure S1: Antibacterial and antibiofilm activity of quinolines (HQ) and derivatives (QD) on *S. aureus* strains. Data points represented as % mean inhibition ± SD.

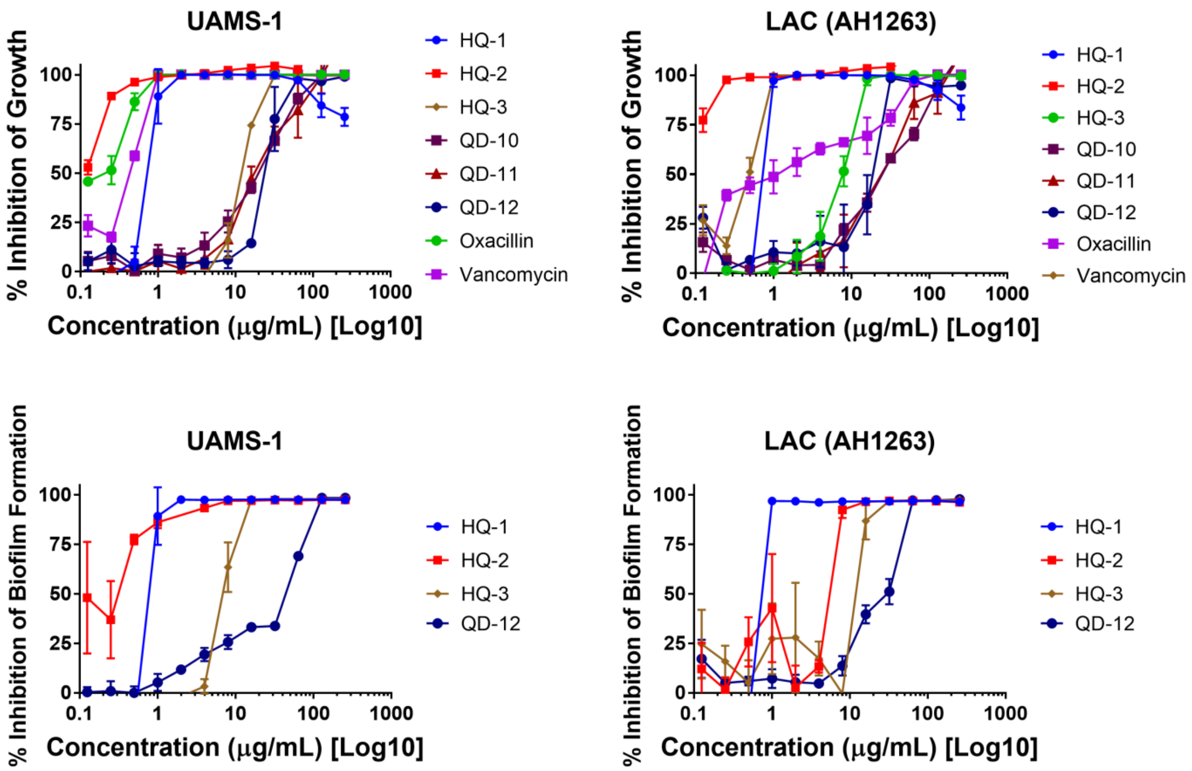

Figure S2: Top performers of the compounds tested against *S. aureus*. Data points represented as % mean inhibition ± SD.

4. Spectroscopic data

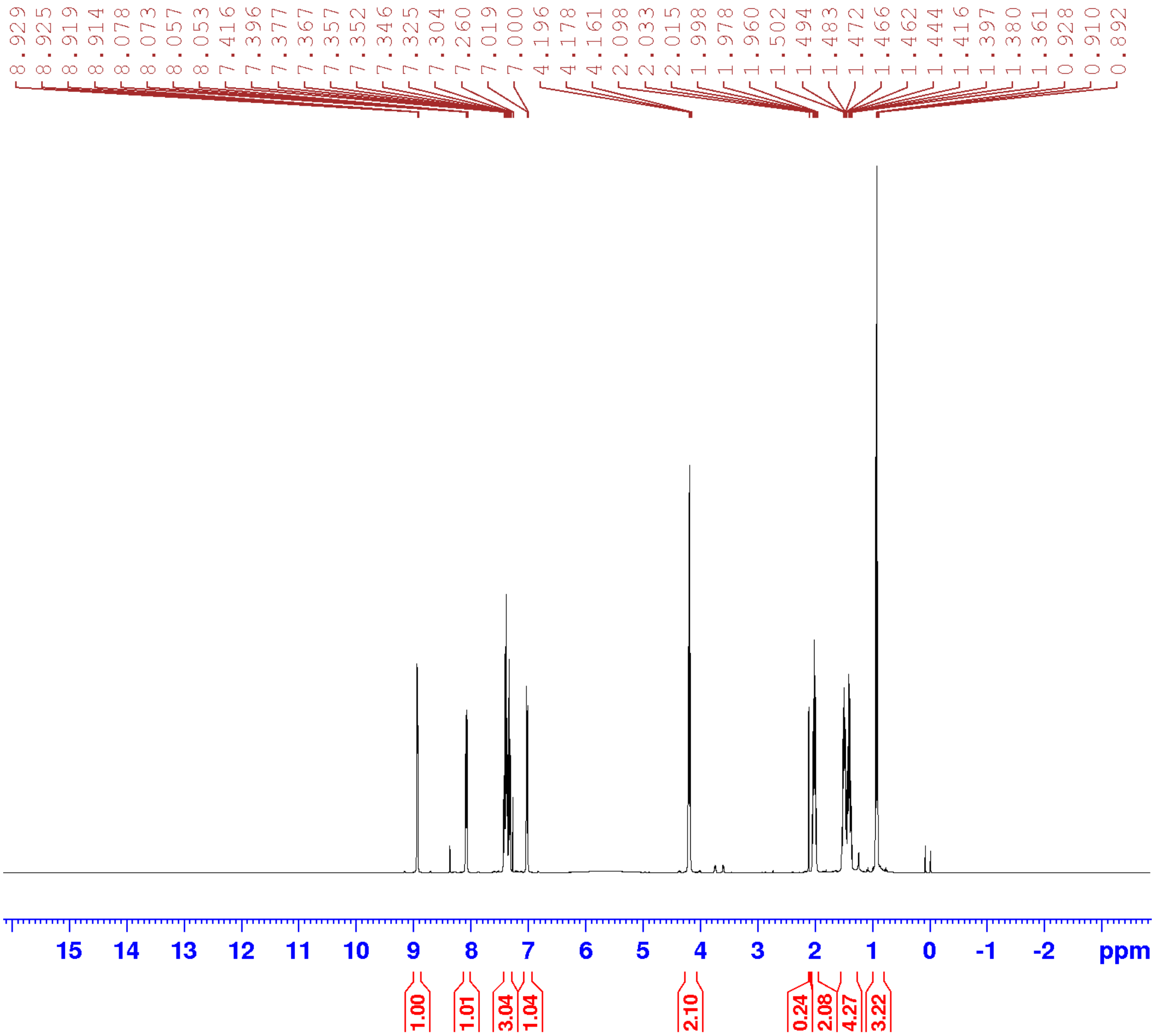

Figure S3: ^{1}H NMR of QD-1

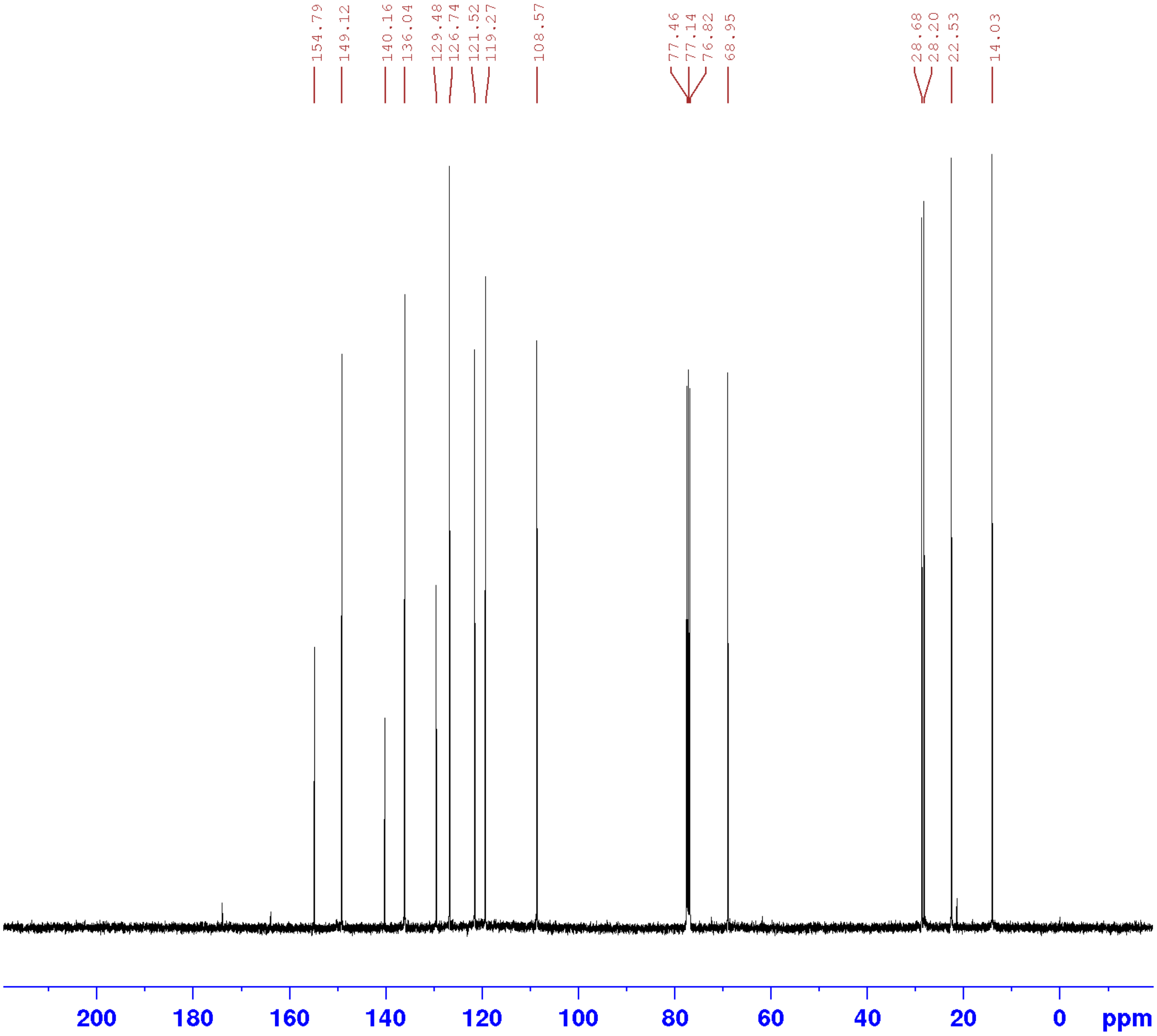

Figure S4: ^{13}C NMR of QD-1

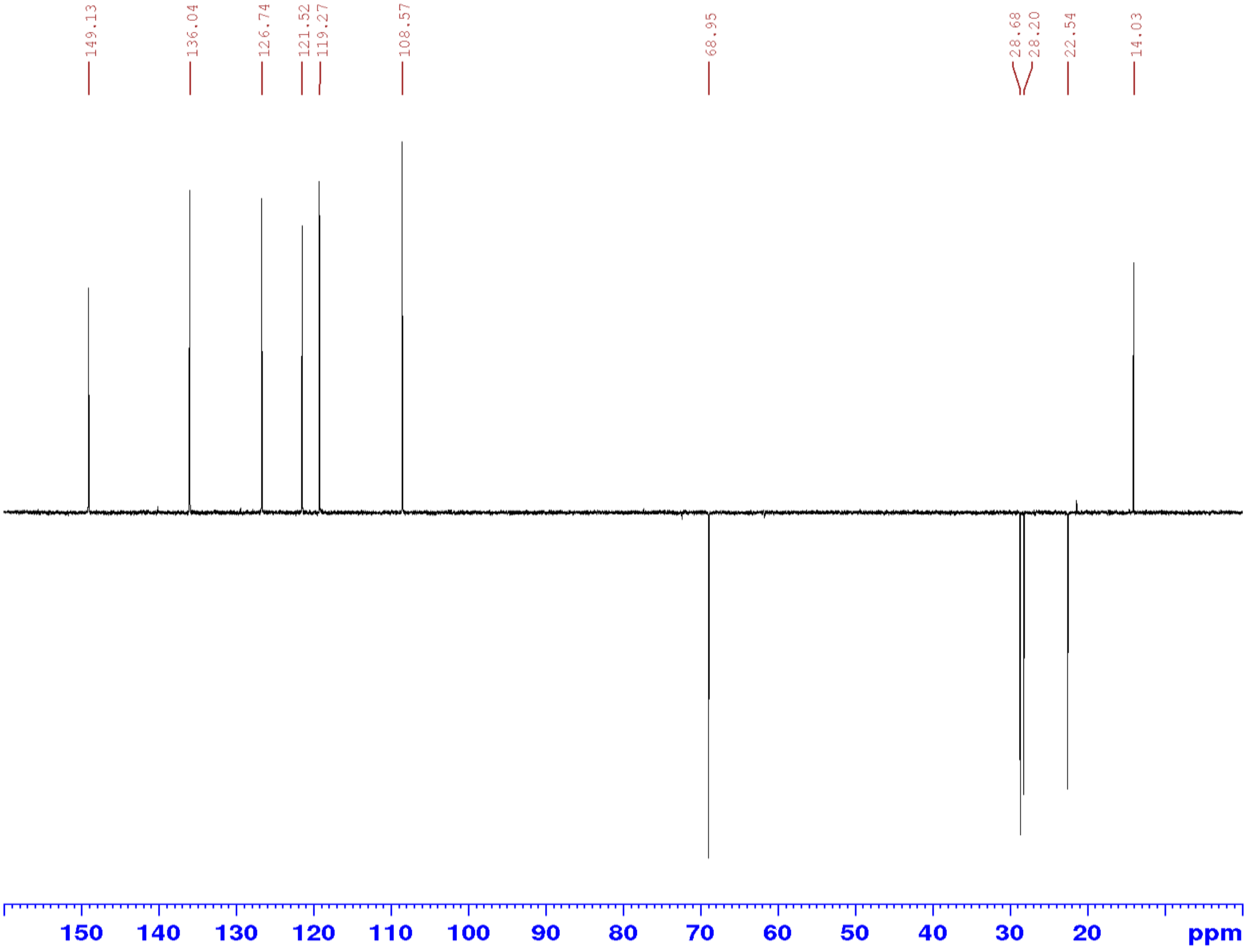

Figure S5: DEPT-135 NMR of QD-1

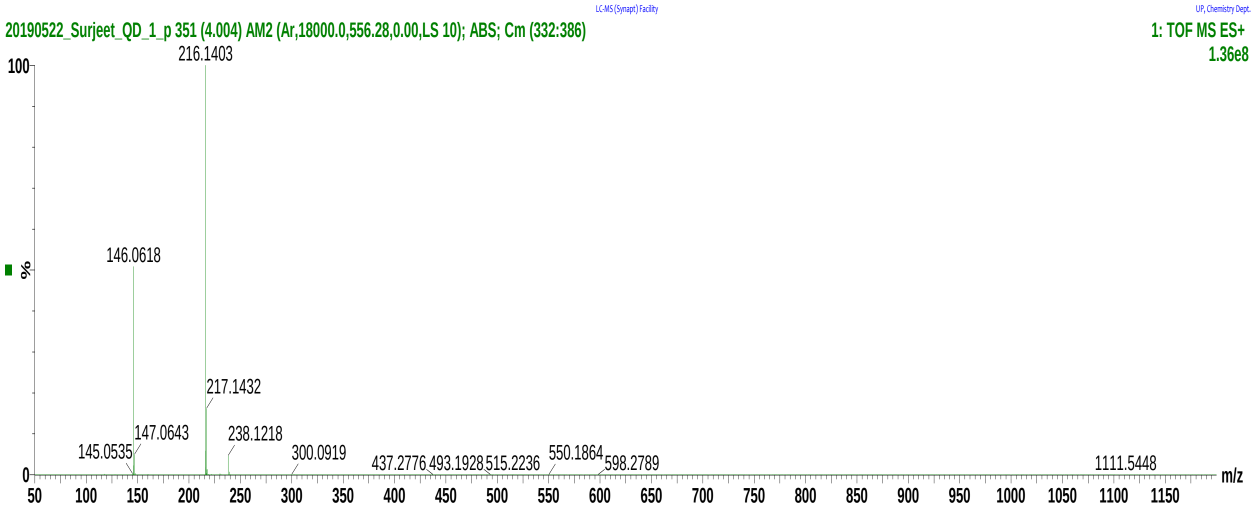

Figure S6: HRESI-MS of QD-1

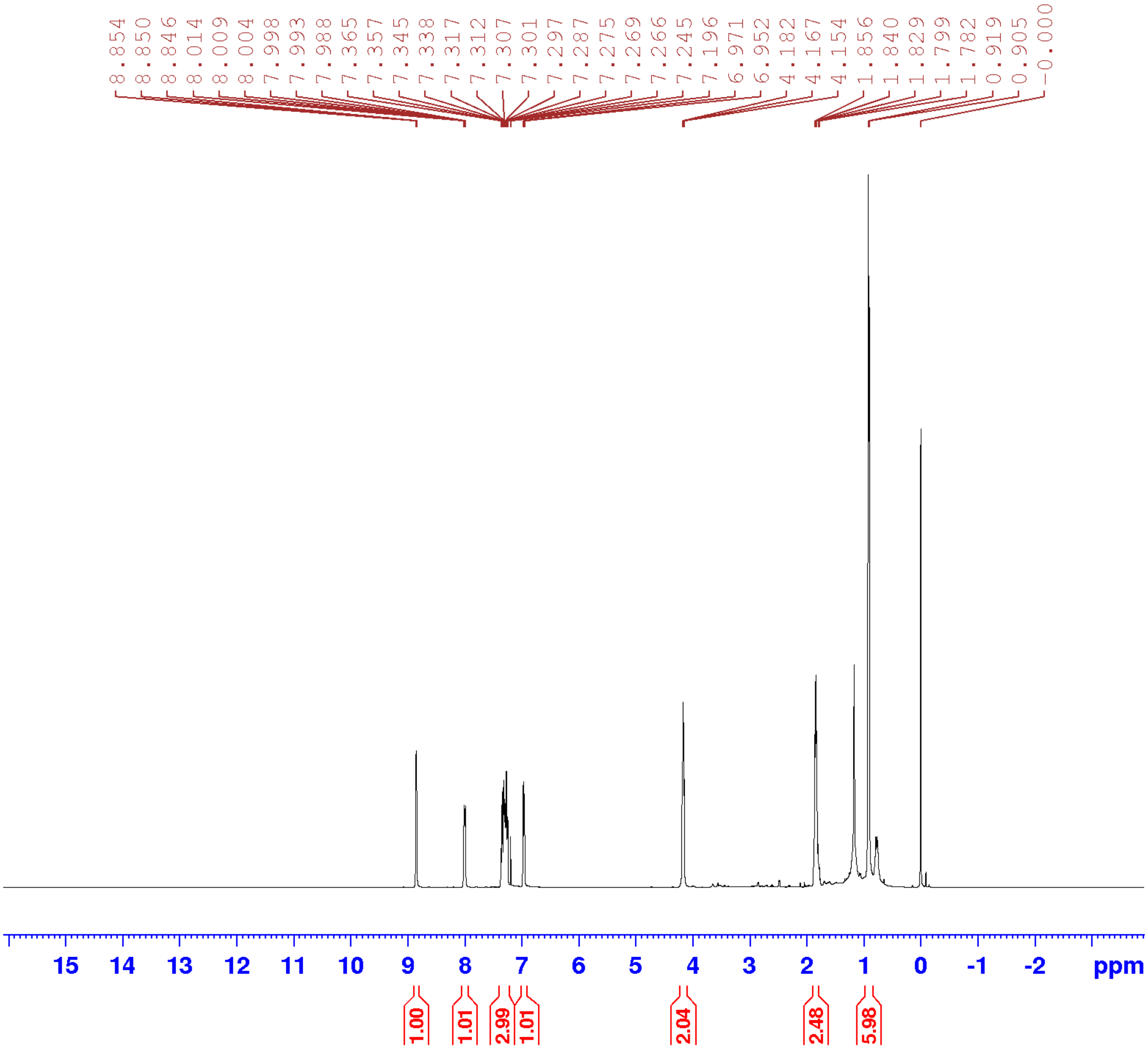

Figure S7: ^{1}H NMR of QD-2

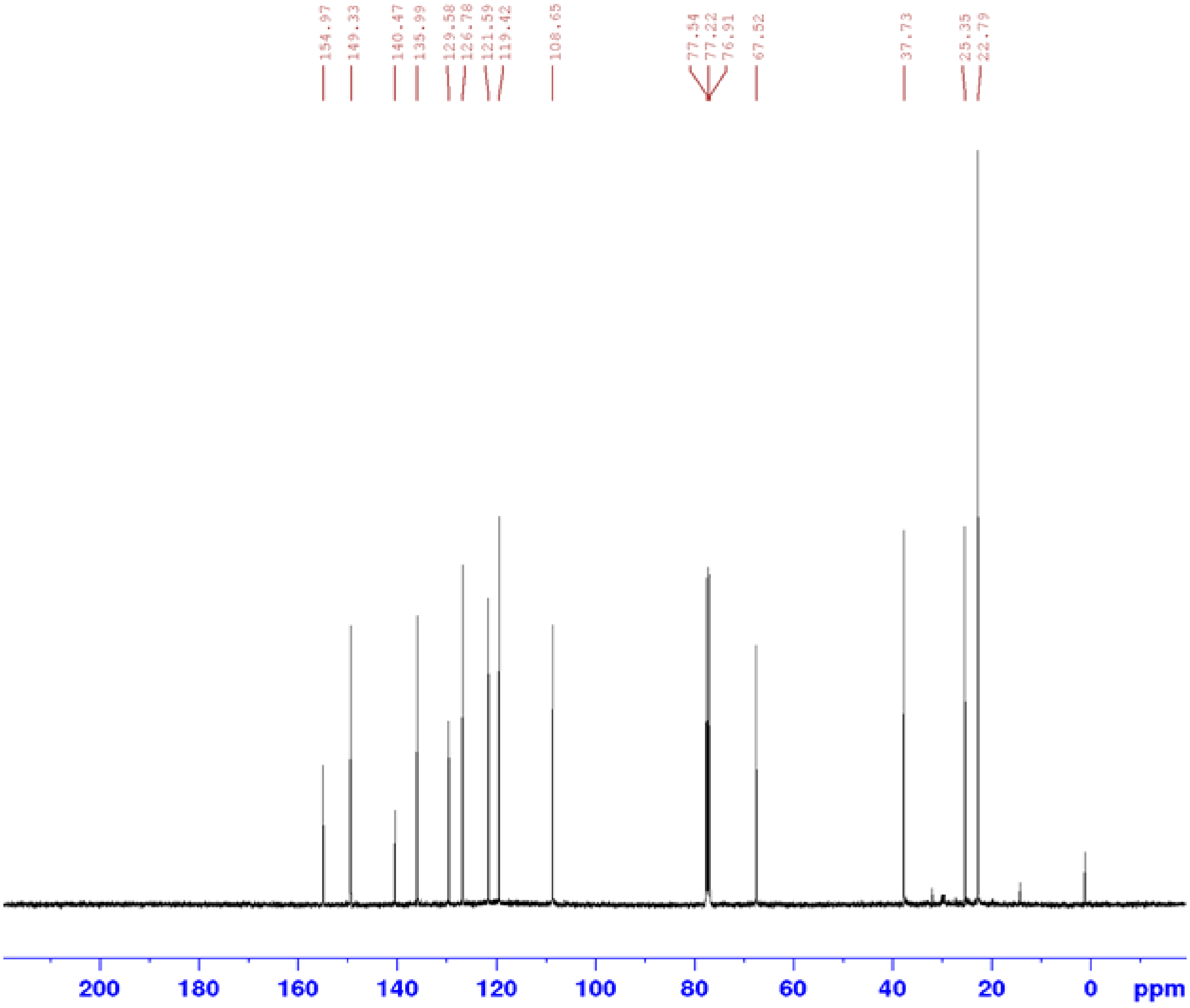

Figure S8: ^{13}C NMR of QD-2

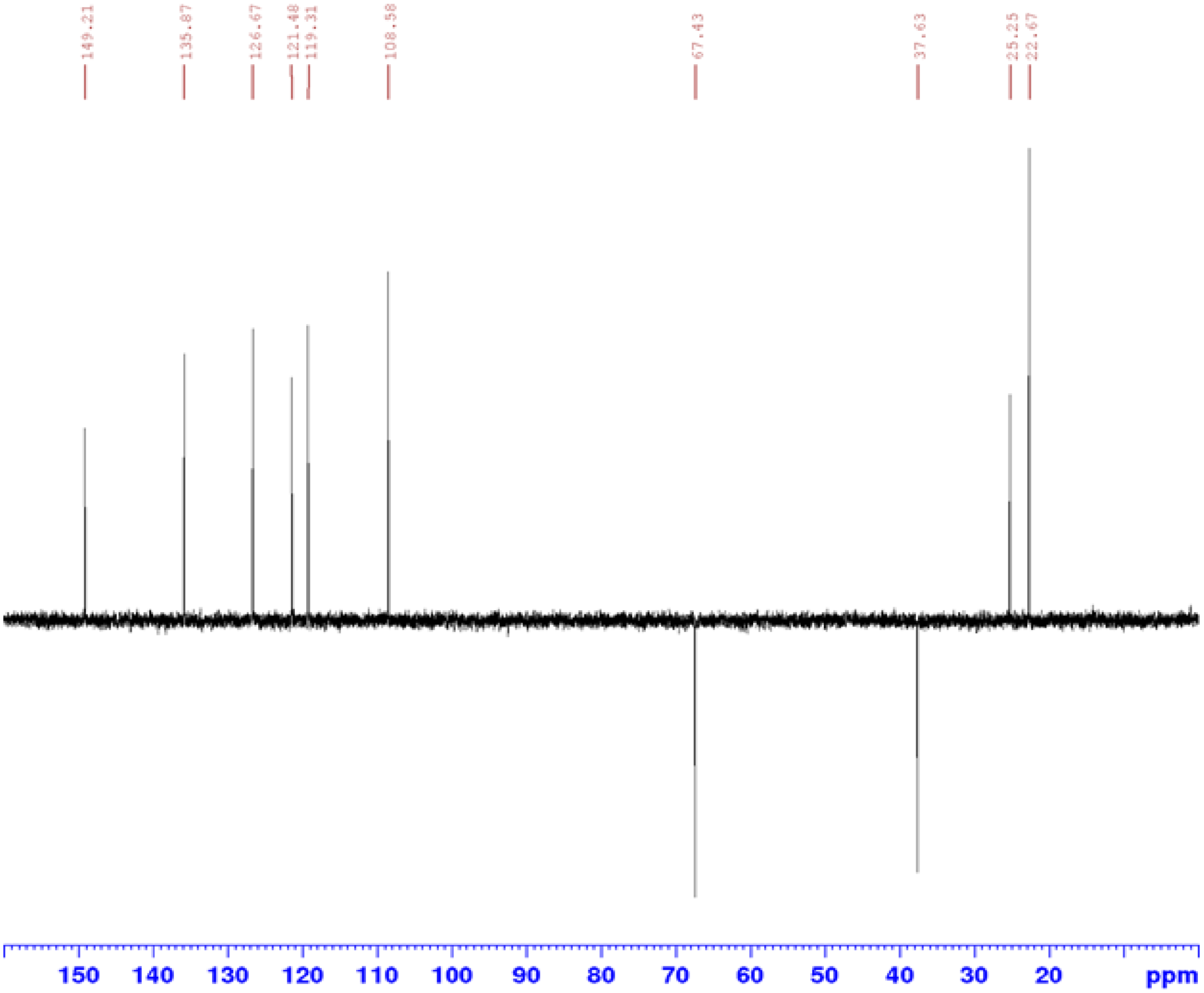

Figure S9: DEPT-135 NMR of QD-2

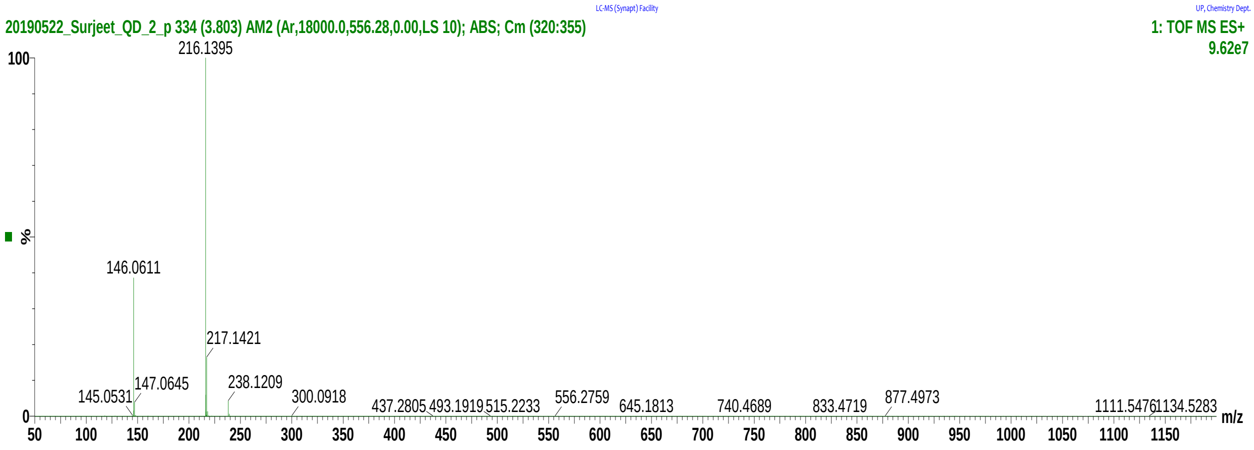

Figure S10: HRESI-MS of QD-2

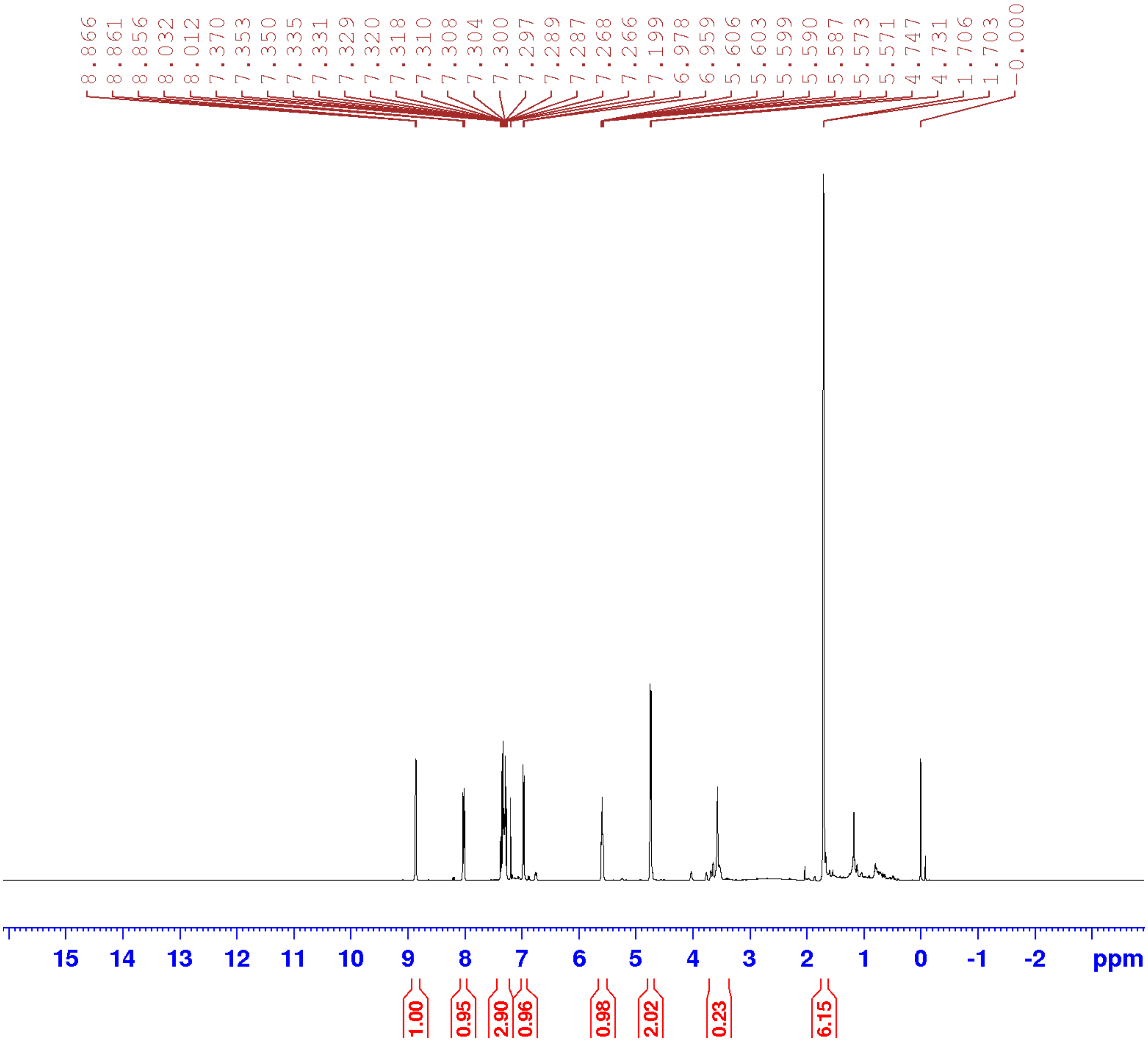

Figure S11: ^{1}H NMR of QD-3

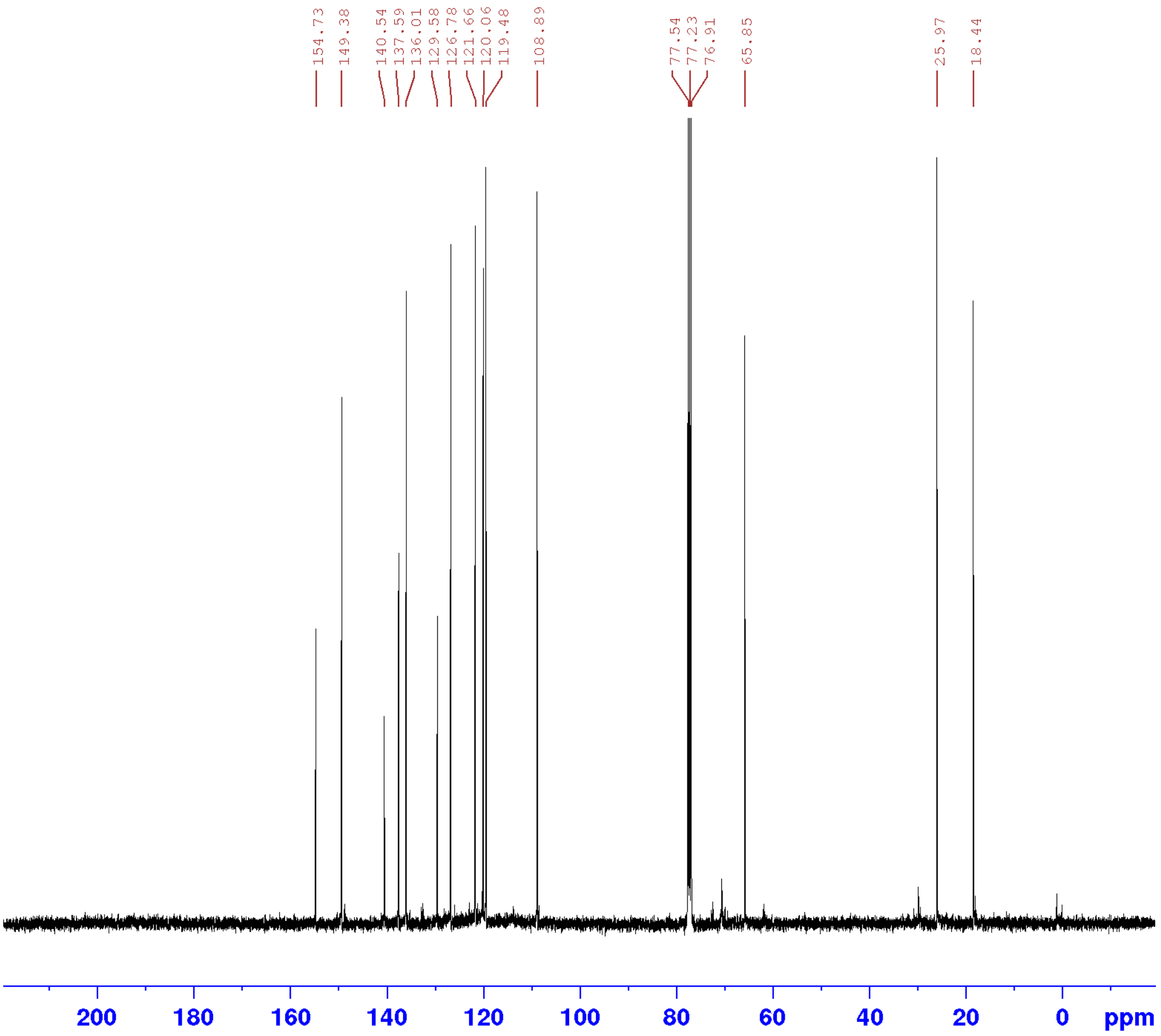

Figure S12: ^{13}C NMR of QD-3

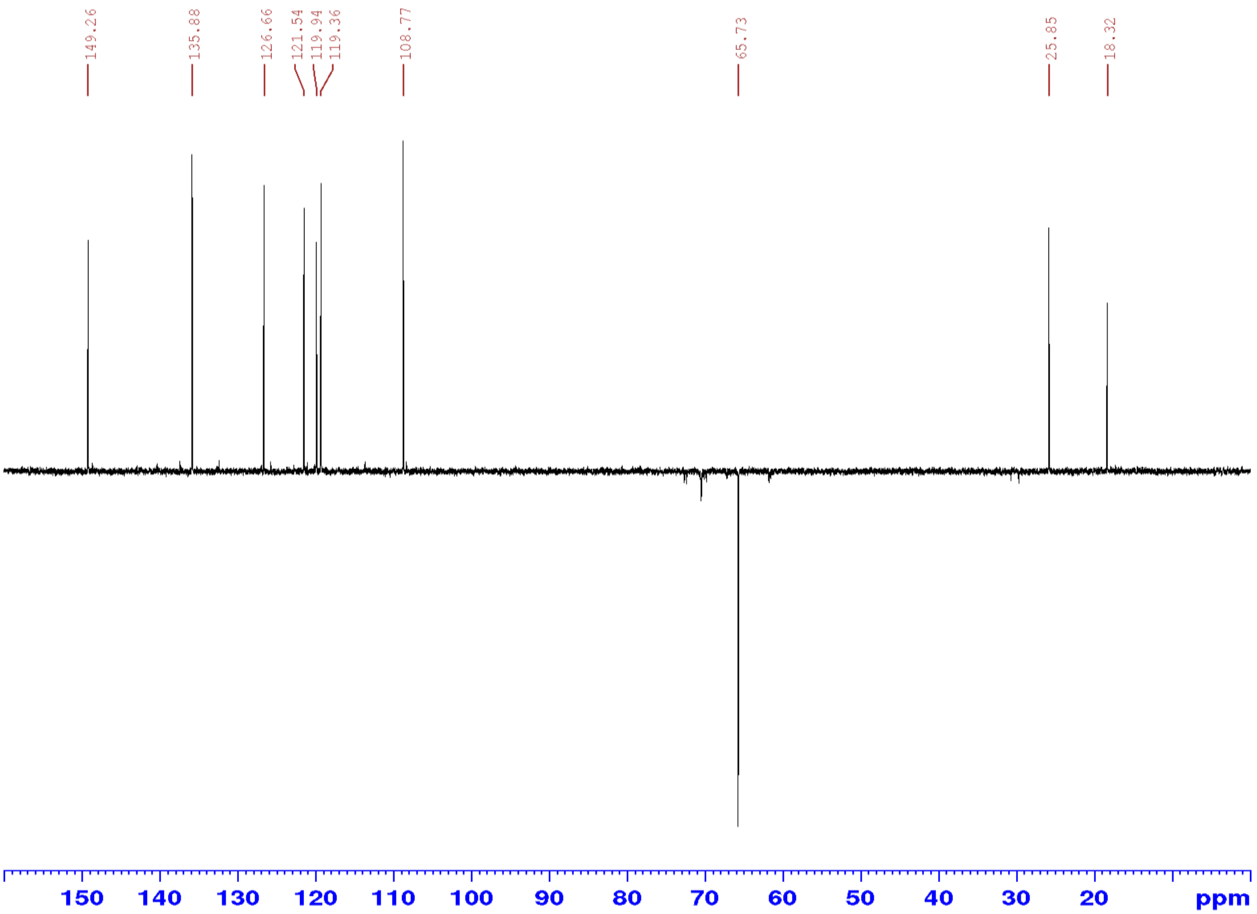

Figure S13: DEPT-135 NMR of QD-3

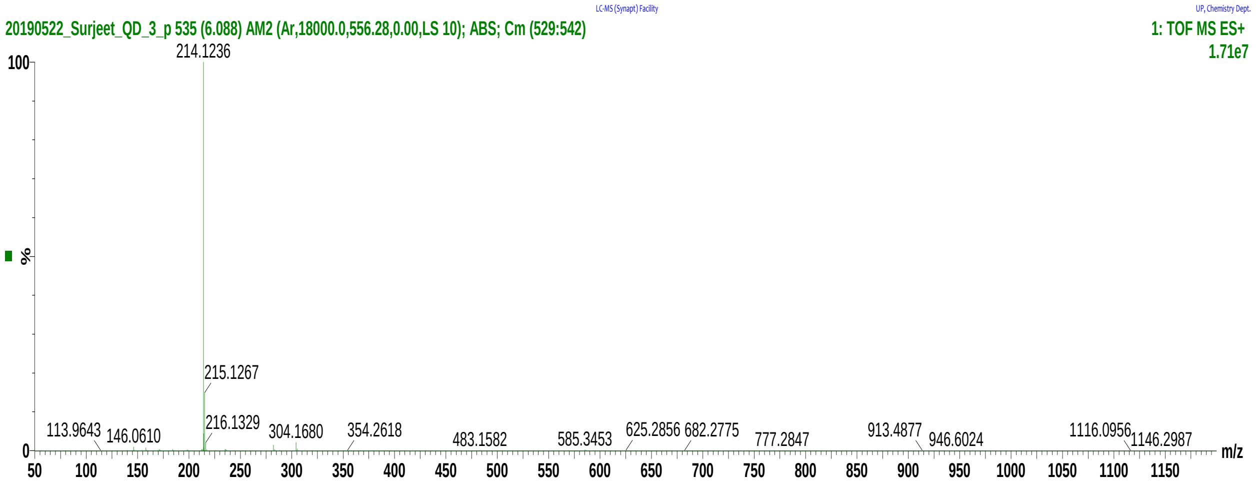

Figure S14: HRESI-MS of QD-3

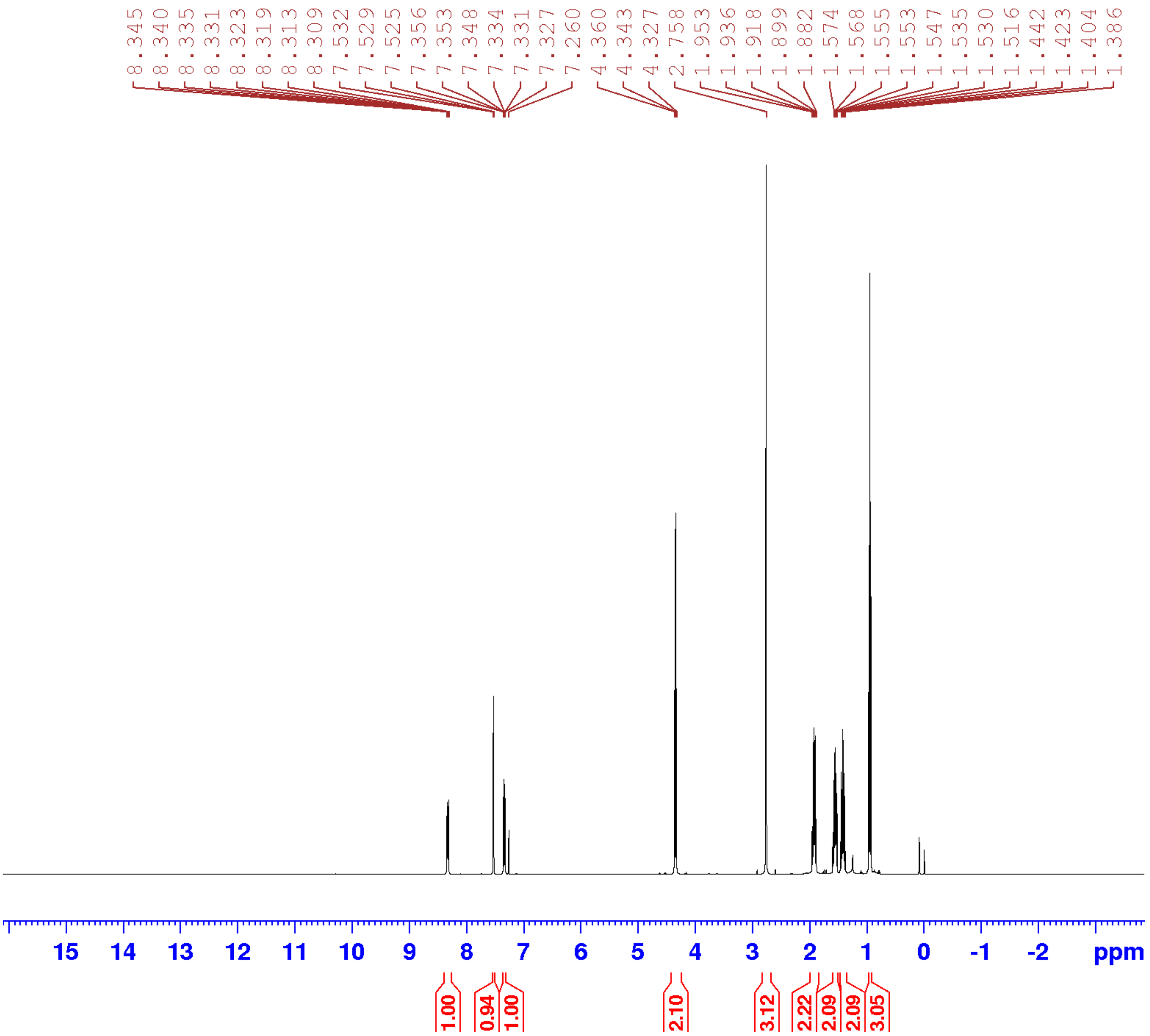

Figure S15: ^{1}H NMR of QD-4

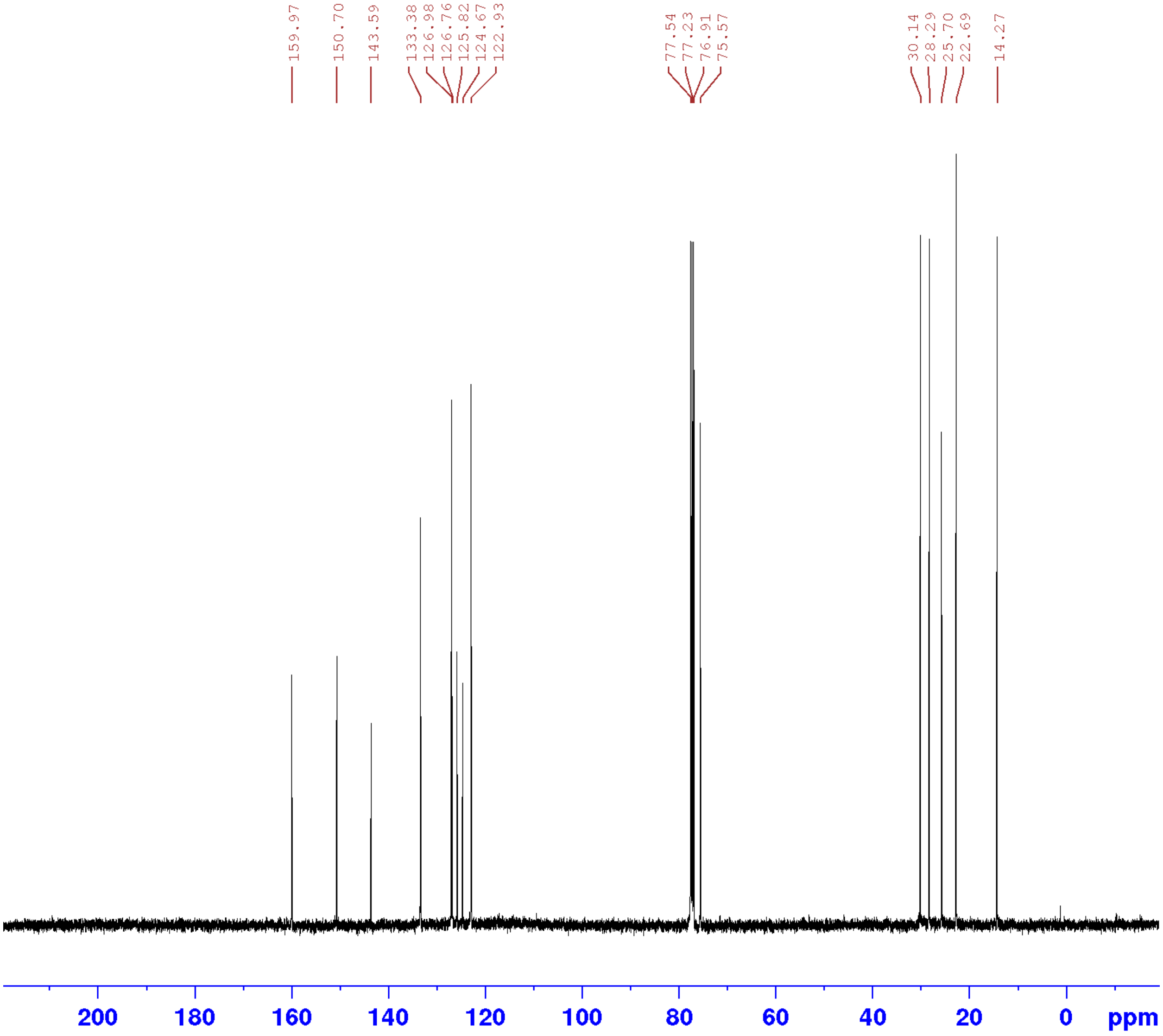

Figure S16: ^{13}C NMR of QD-4

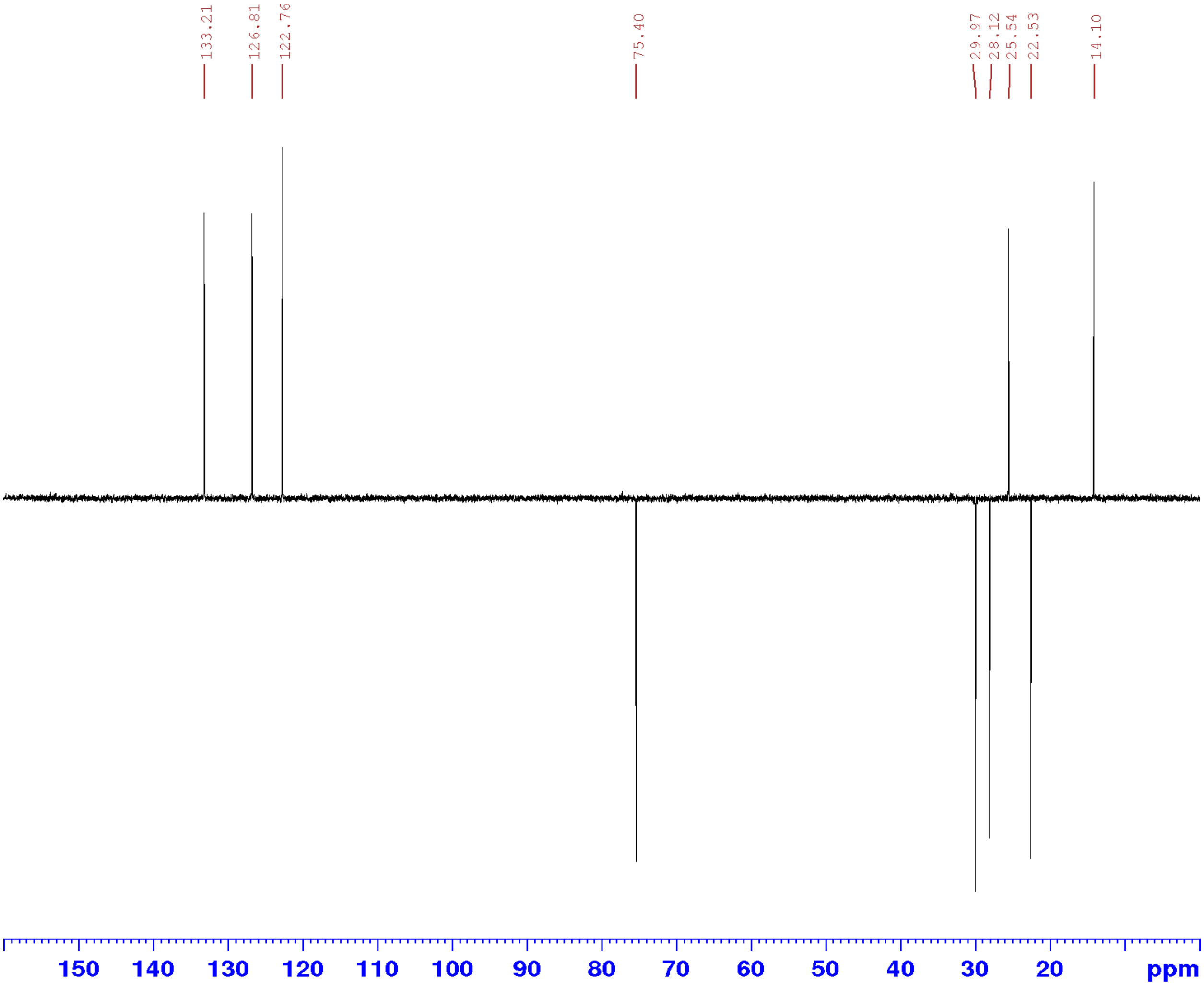

Figure S17: DEPT-135 NMR of QD-4

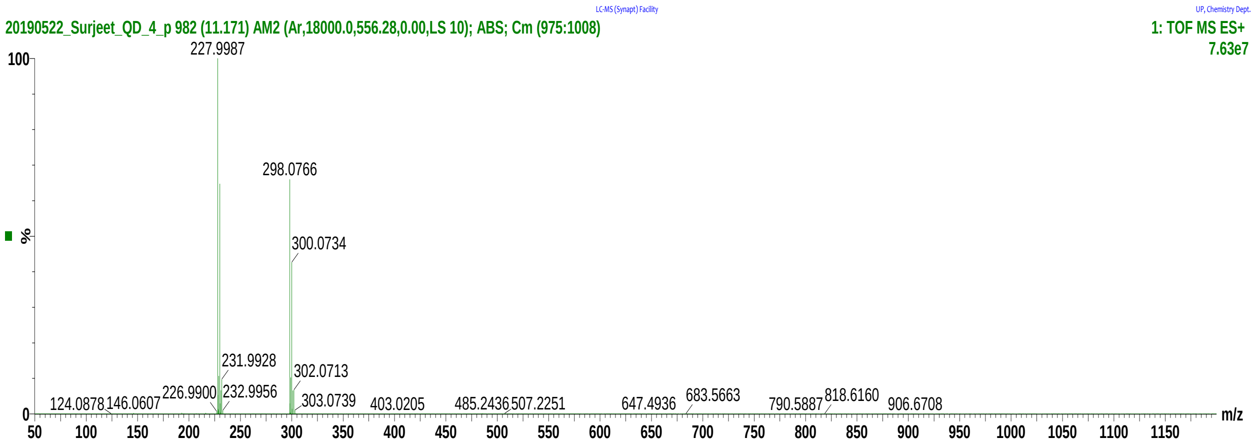

Figure S18: HRESI-MS of QD-4

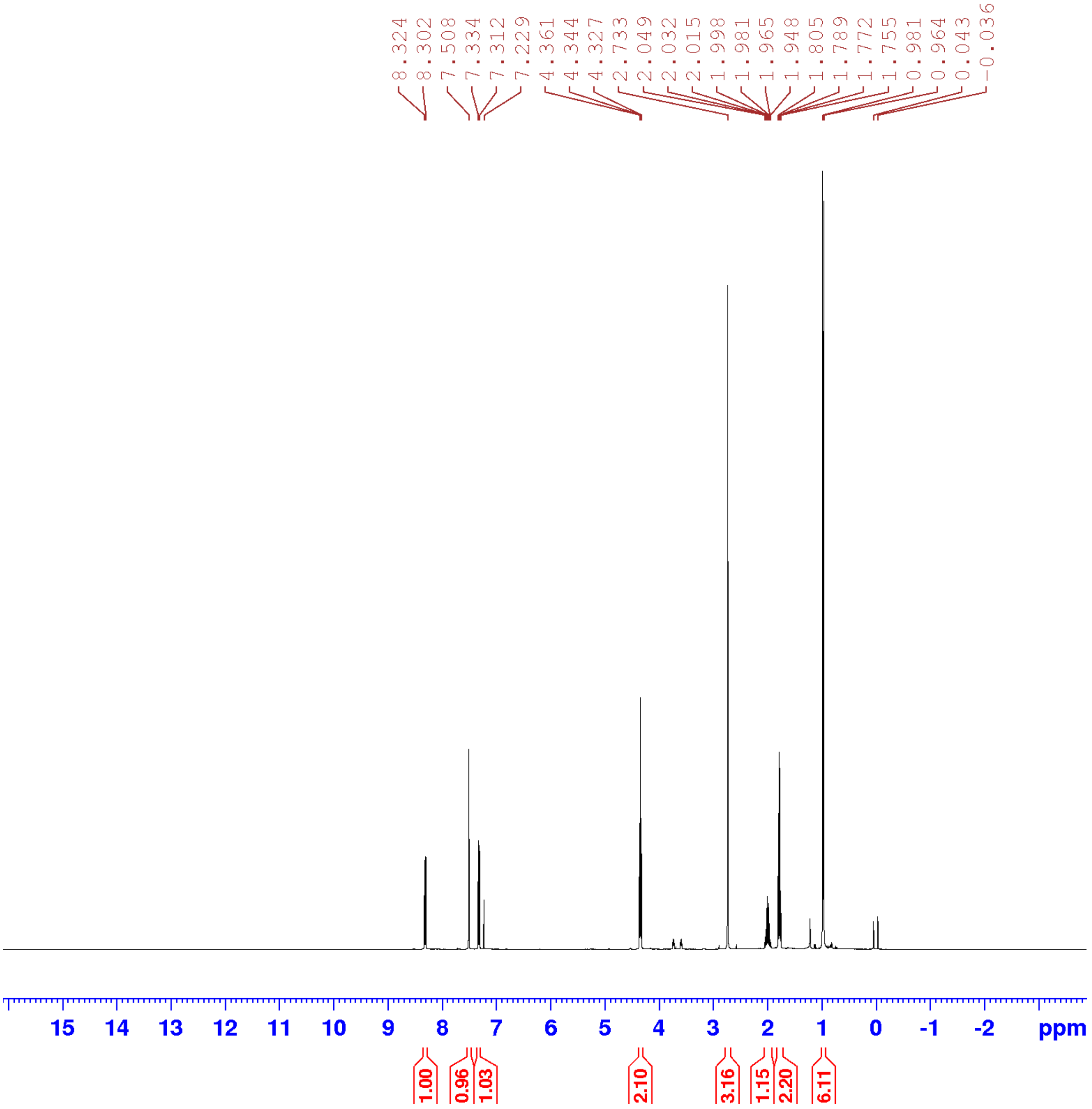

Figure S19: ^{1}H NMR of QD-5

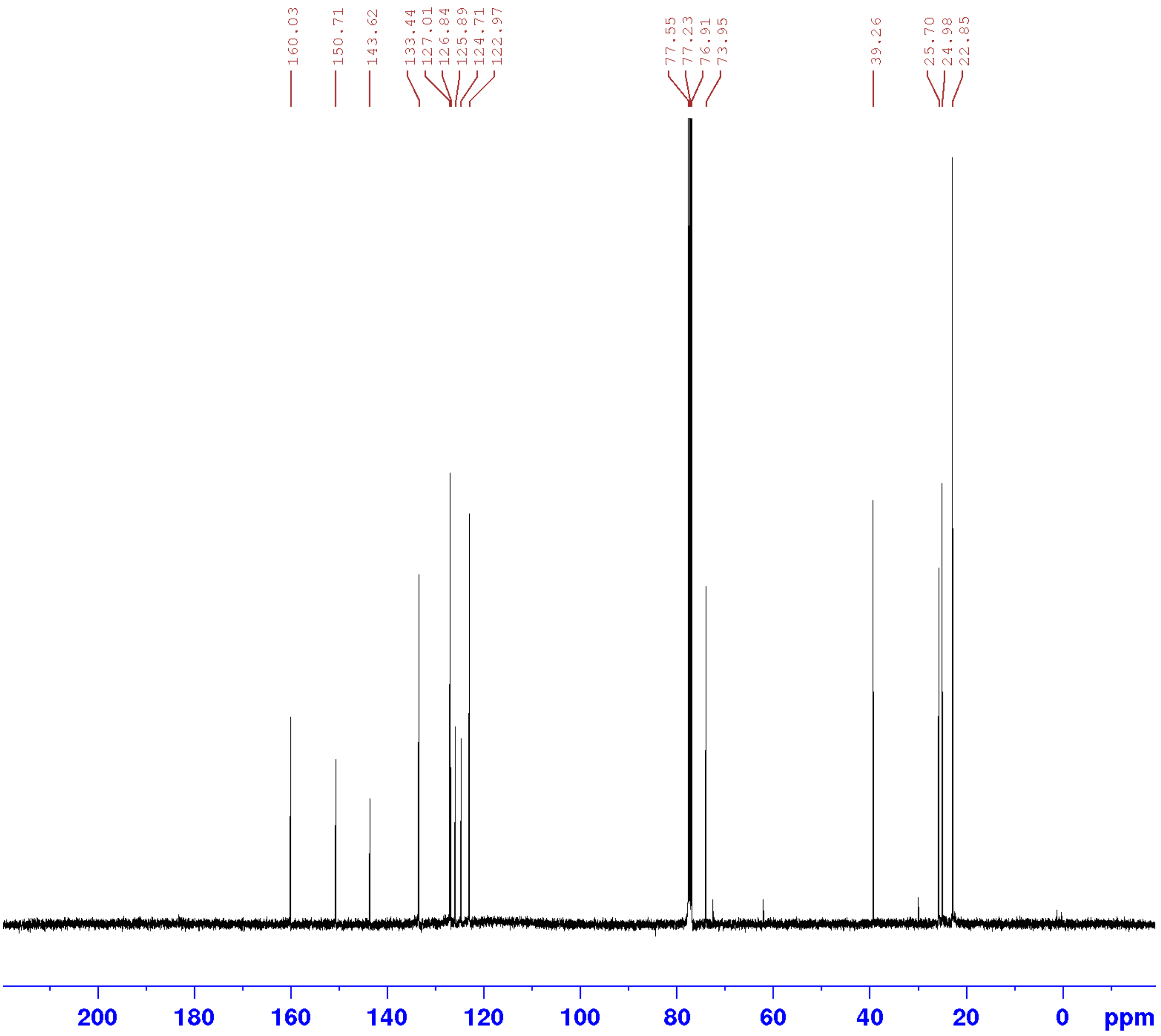

Figure S20: ^{13}C NMR of QD-5

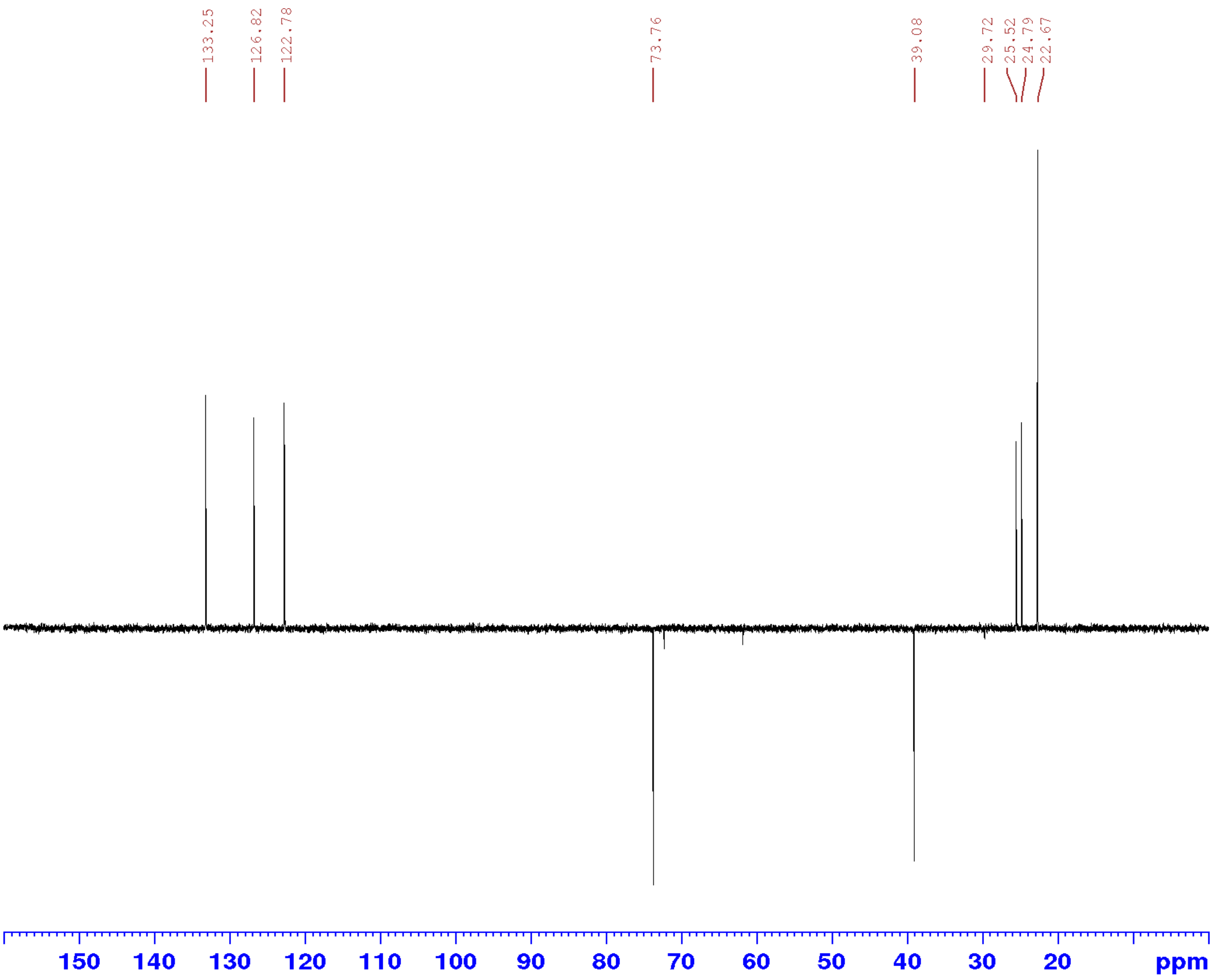

Figure S21: DEPT-135 NMR of QD-5

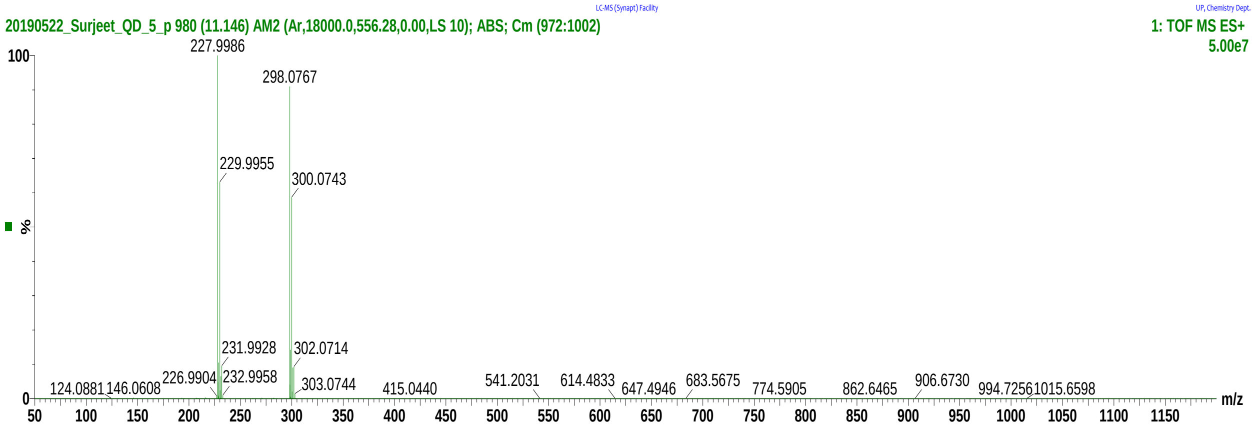

Figure S22: HRESI-MS of QD-5

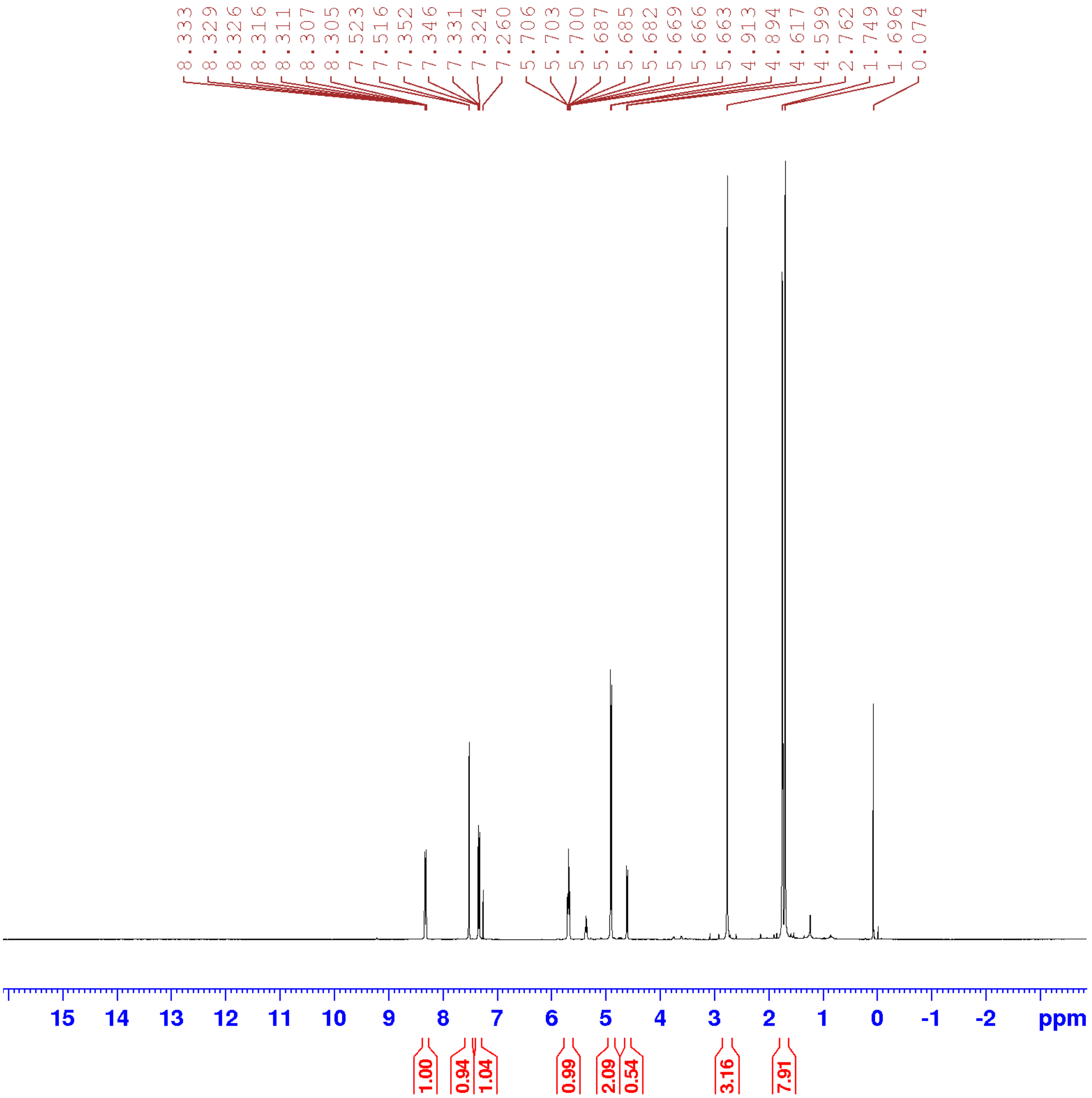

Figure S23: ^{1}H NMR of QD-6

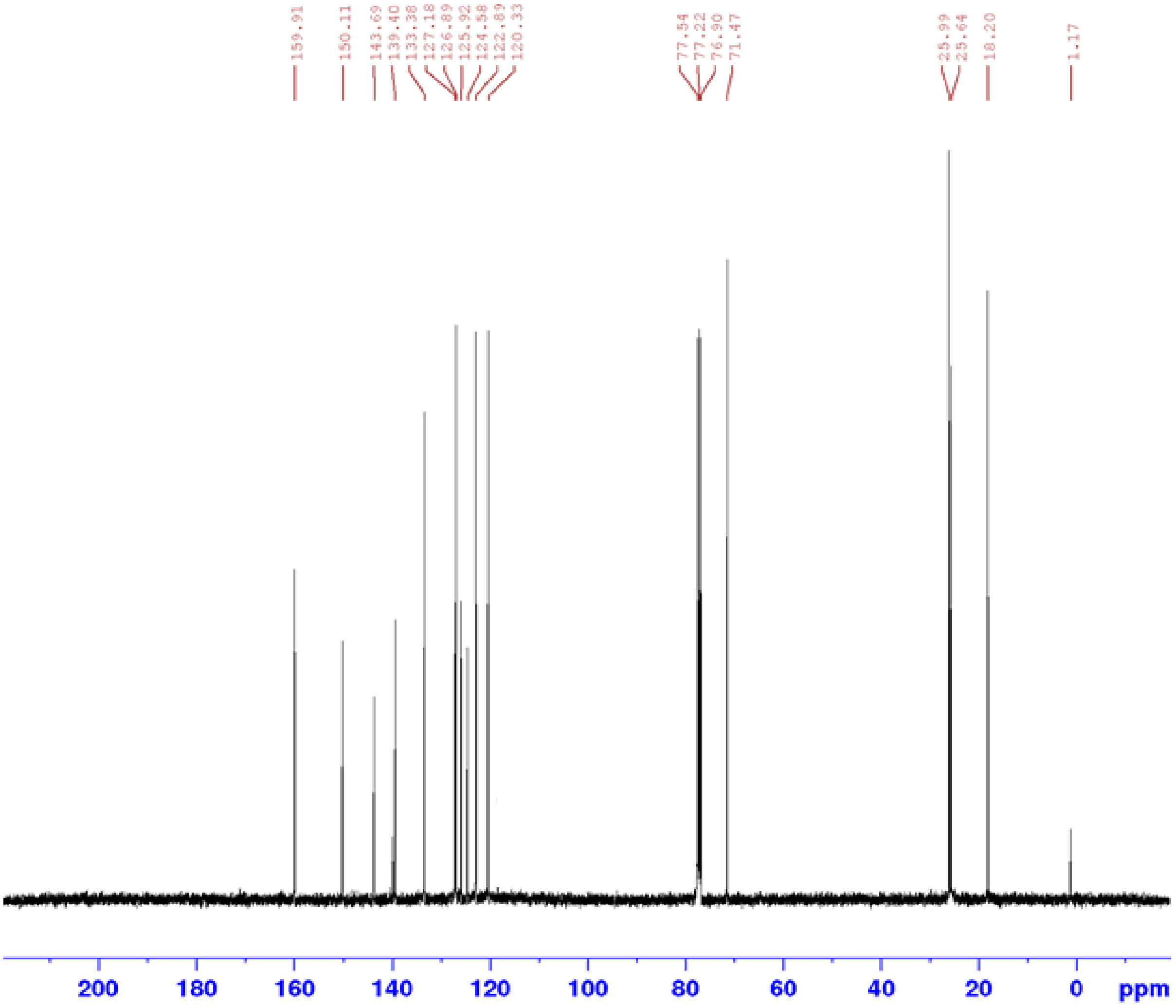

Figure S24: ^{13}C NMR of QD-6

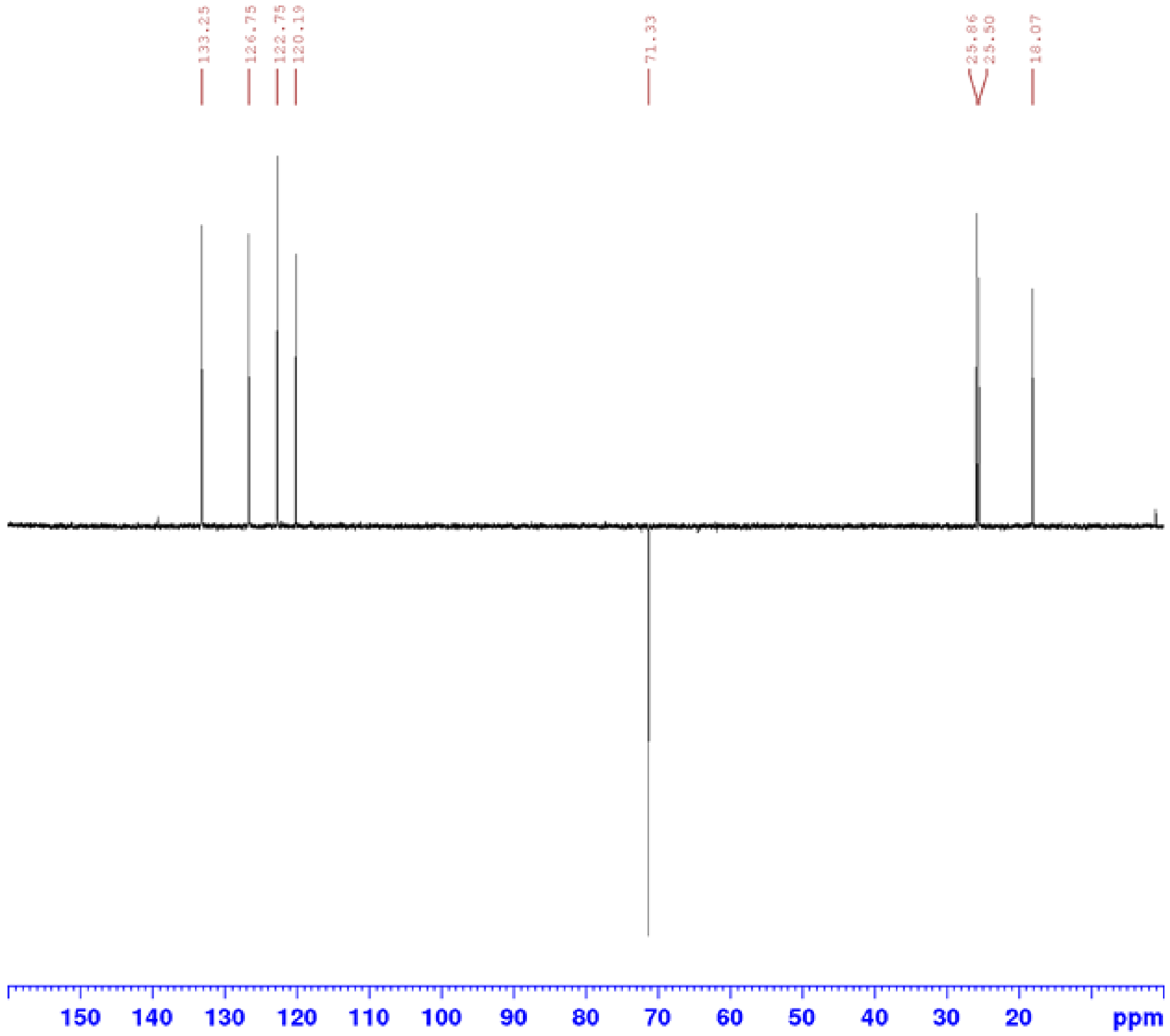

Figure S25: DEPT-135 NMR of QD-6

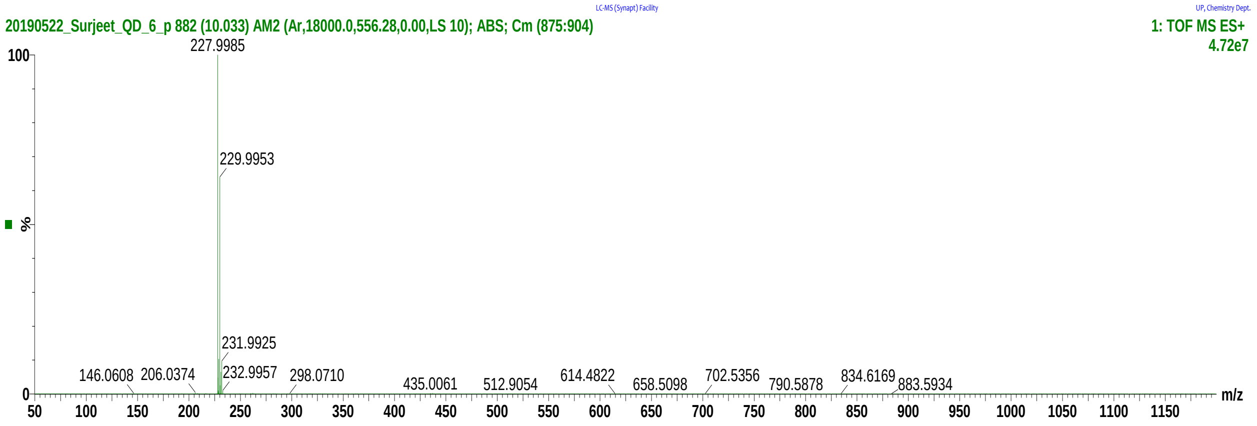

Figure S26: HRESI-MS of QD-6

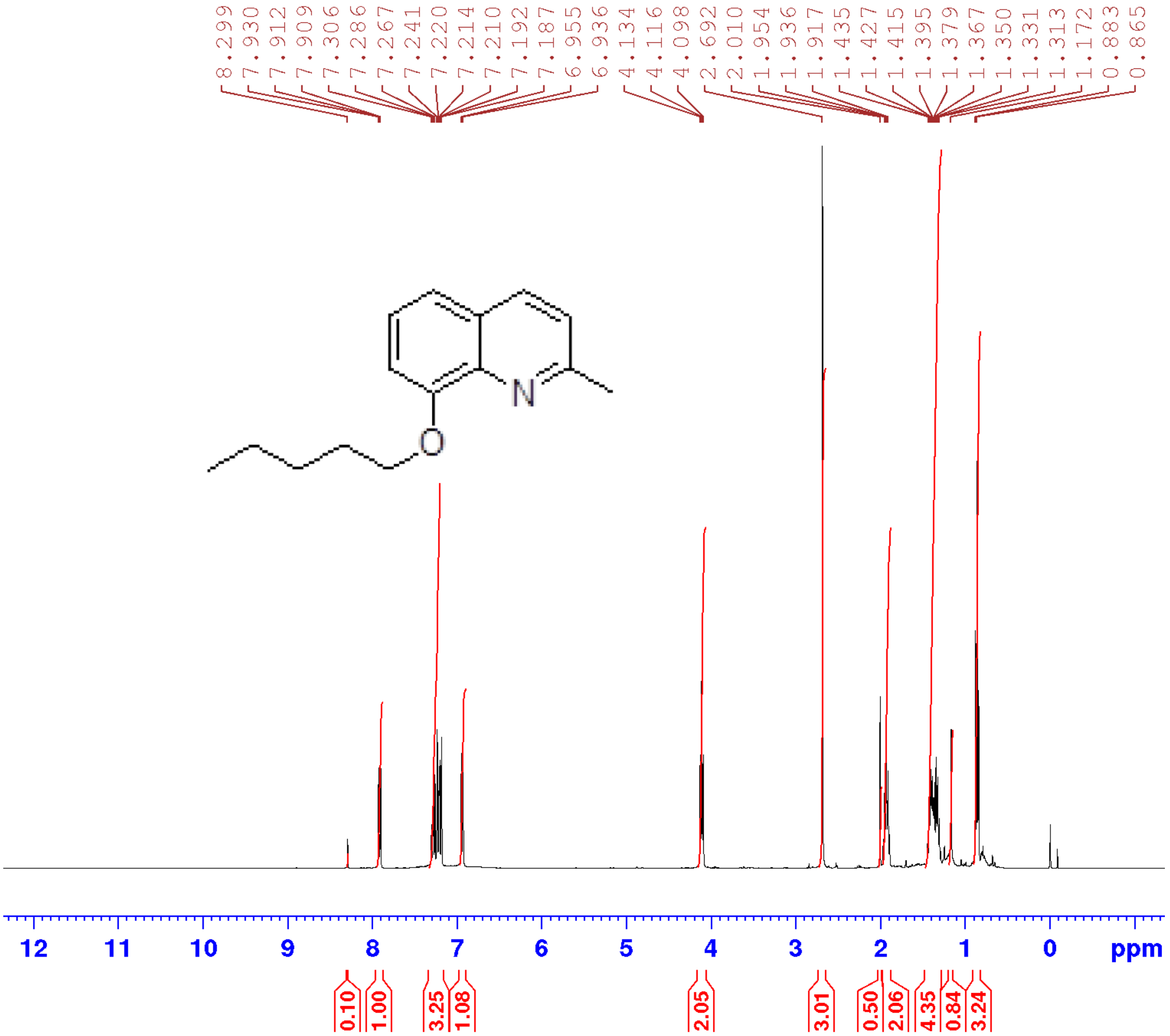
8.299
7.930
7.912
7.909
7.306
7.286
7.267
7.241
7.220
7.214
7.210
7.192
7.187
6.955
6.936
4.134
4.116
4.098
2.692
2.010
1.954
1.936
1.917
1.435
1.427
1.415
1.395
1.379
1.367
1.350
1.331
1.313
1.172
0.883
0.865
N
O
12 11 10 9 8 7 6 5 4 3 2 1 0 ppm
0.10
1.00
3.25
1.08
2.05
3.01
0.50
2.06
4.35
0.84
3.24

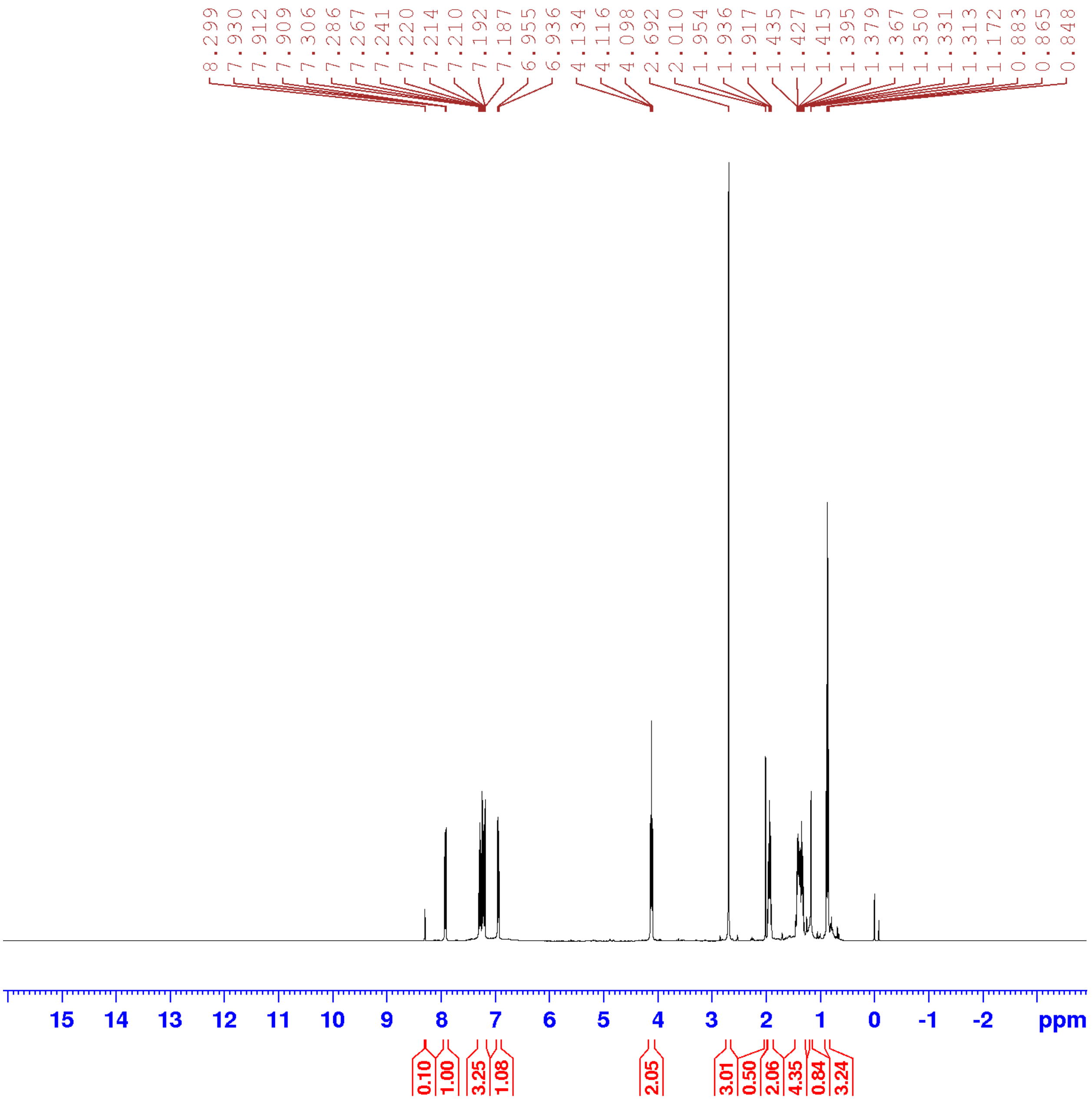

Figure S27: ^{1}H NMR of QD-7

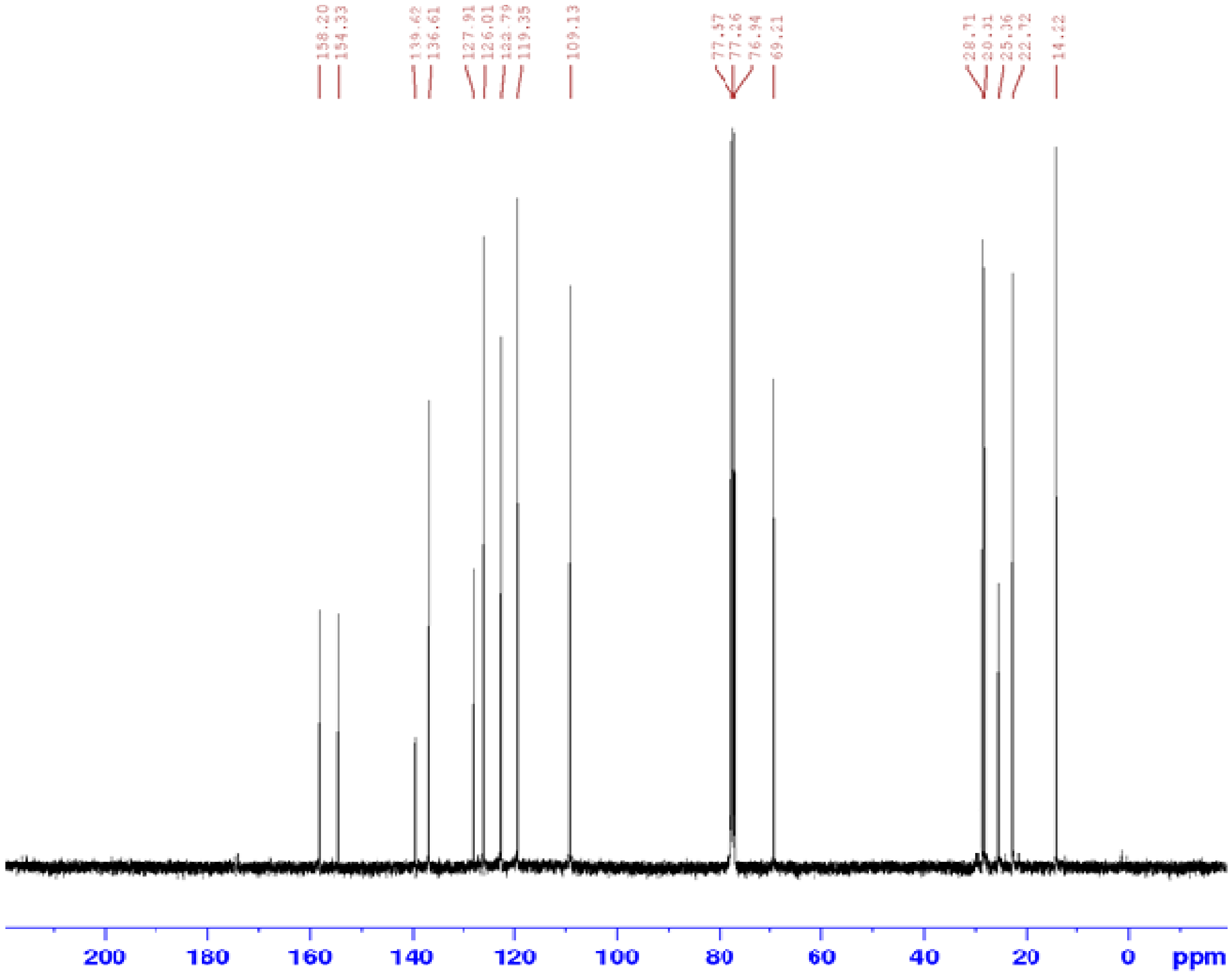

Figure S28: ^{13}C NMR of QD-7

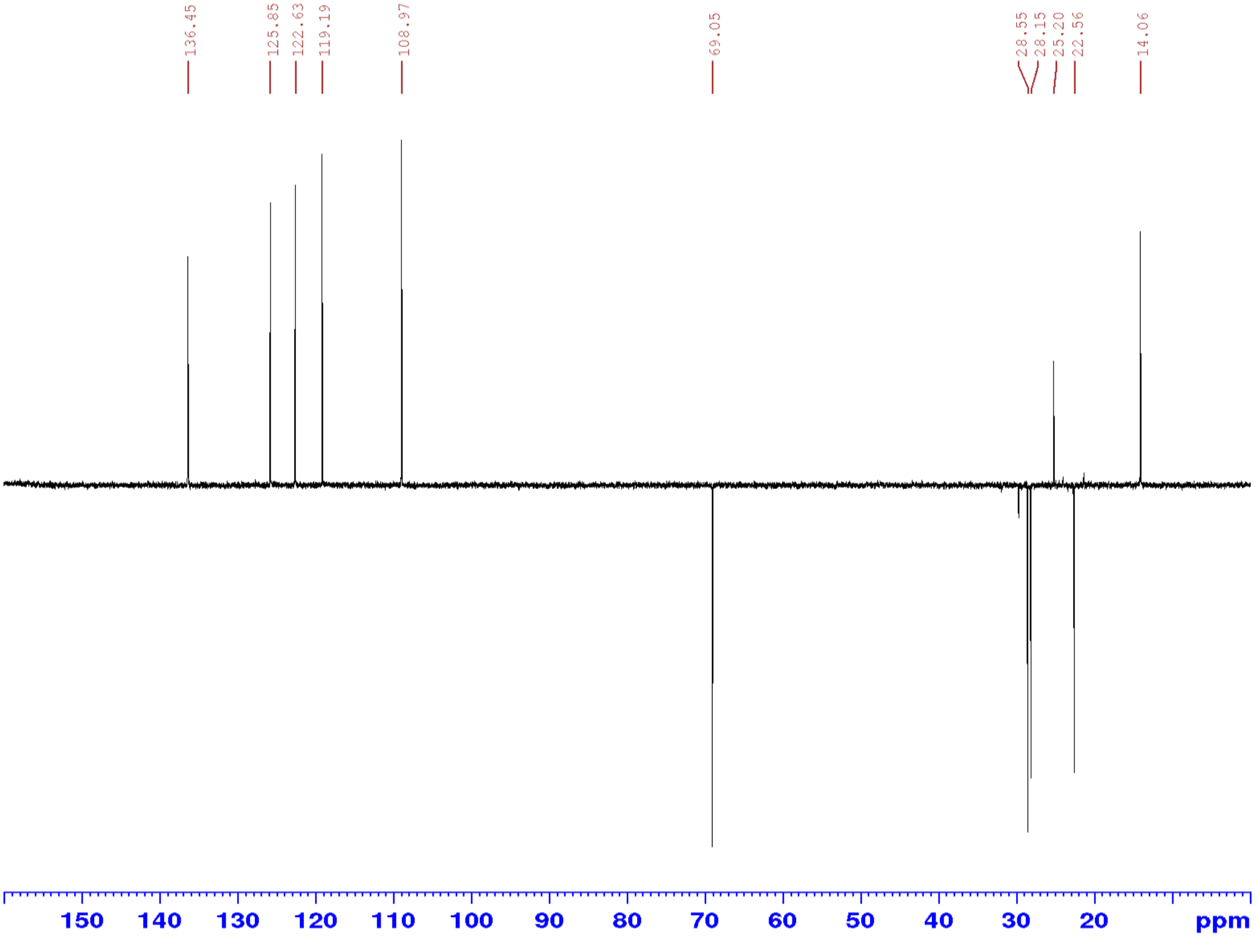

Figure S29: DEPT-135 NMR of QD-7

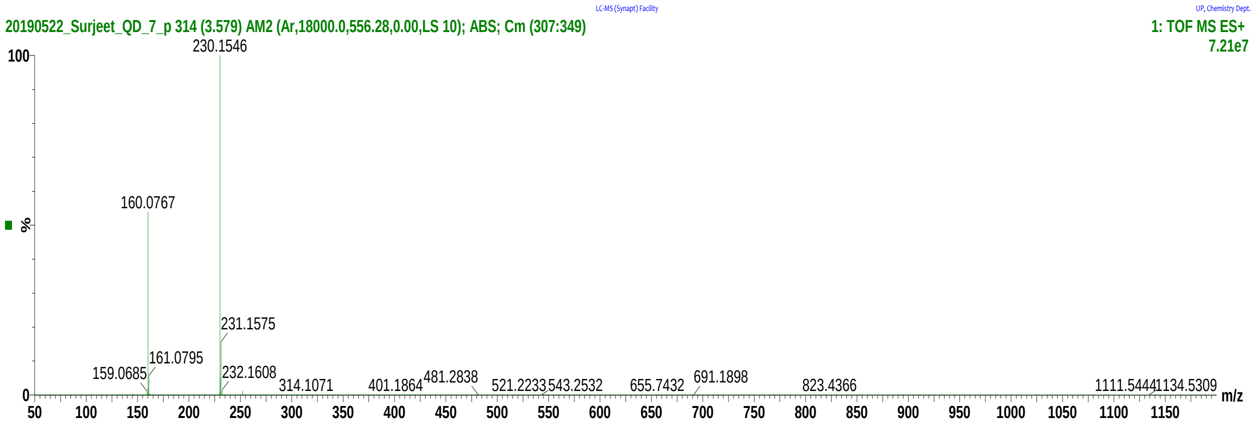

Figure S30: HRESI-MS of QD-7

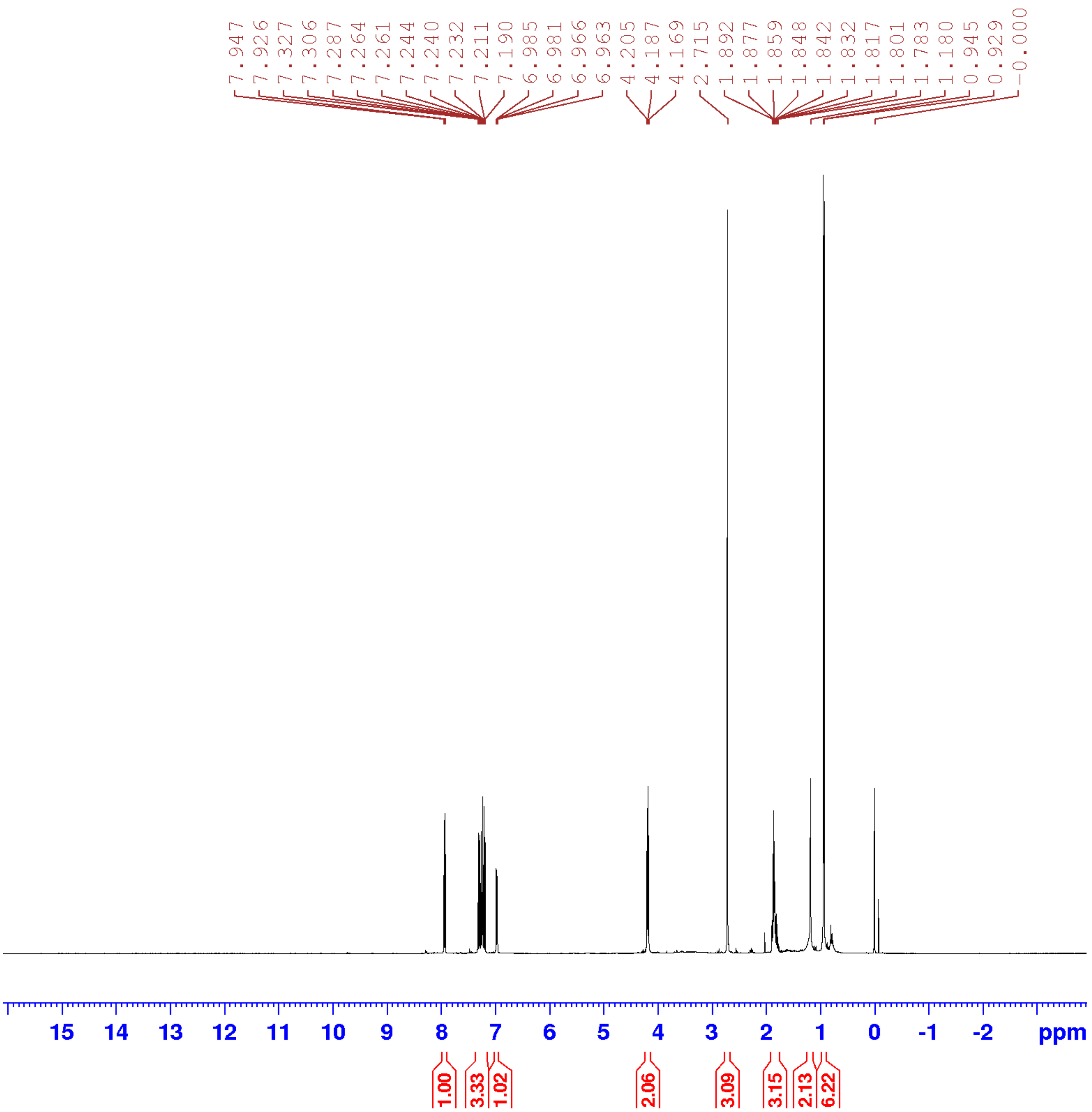

Figure S31: ^{1}H NMR of QD-8

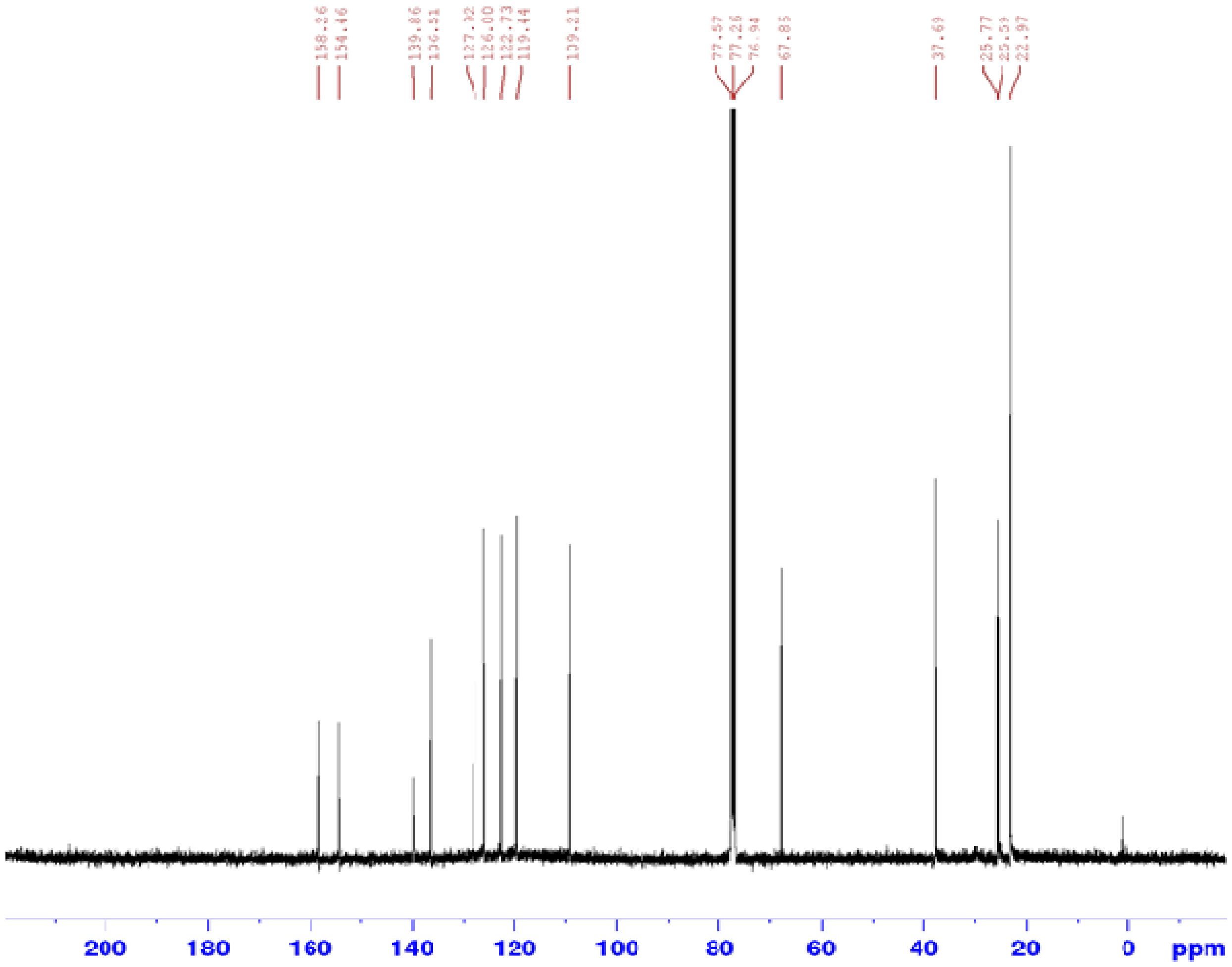

Figure S32: ^{13}C NMR of QD-8

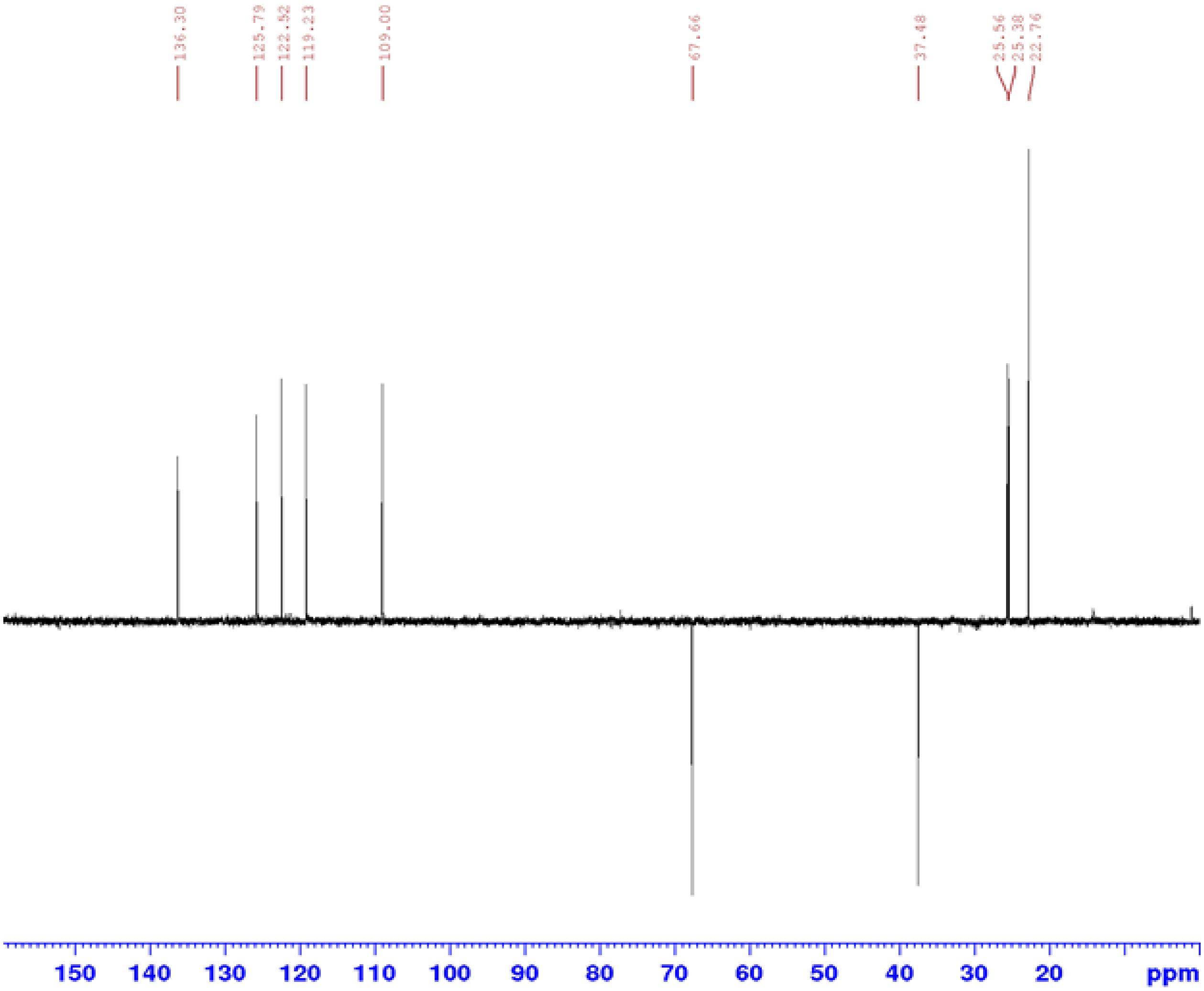

Figure S33: DEPT-135 NMR of QD-8

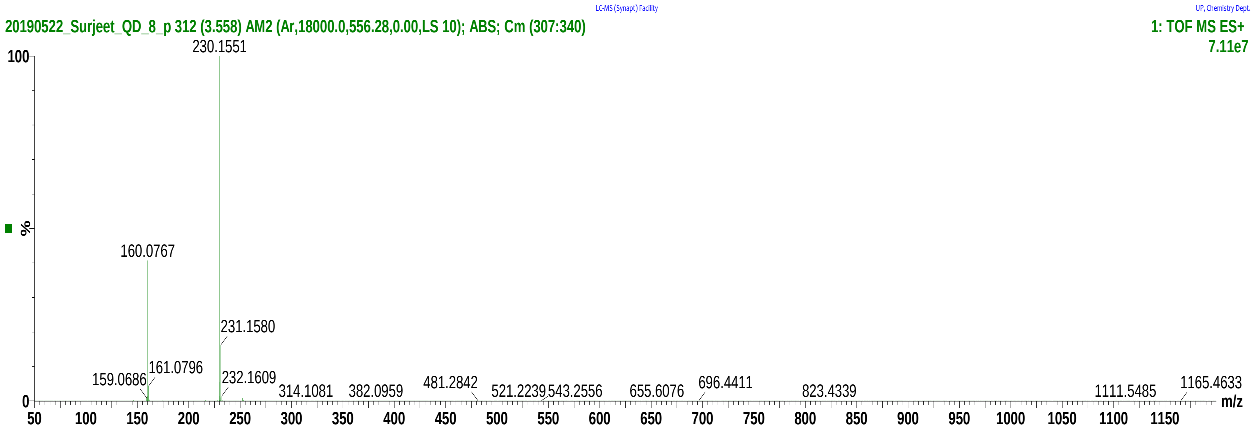

Figure S34: HRESI-MS of QD-8

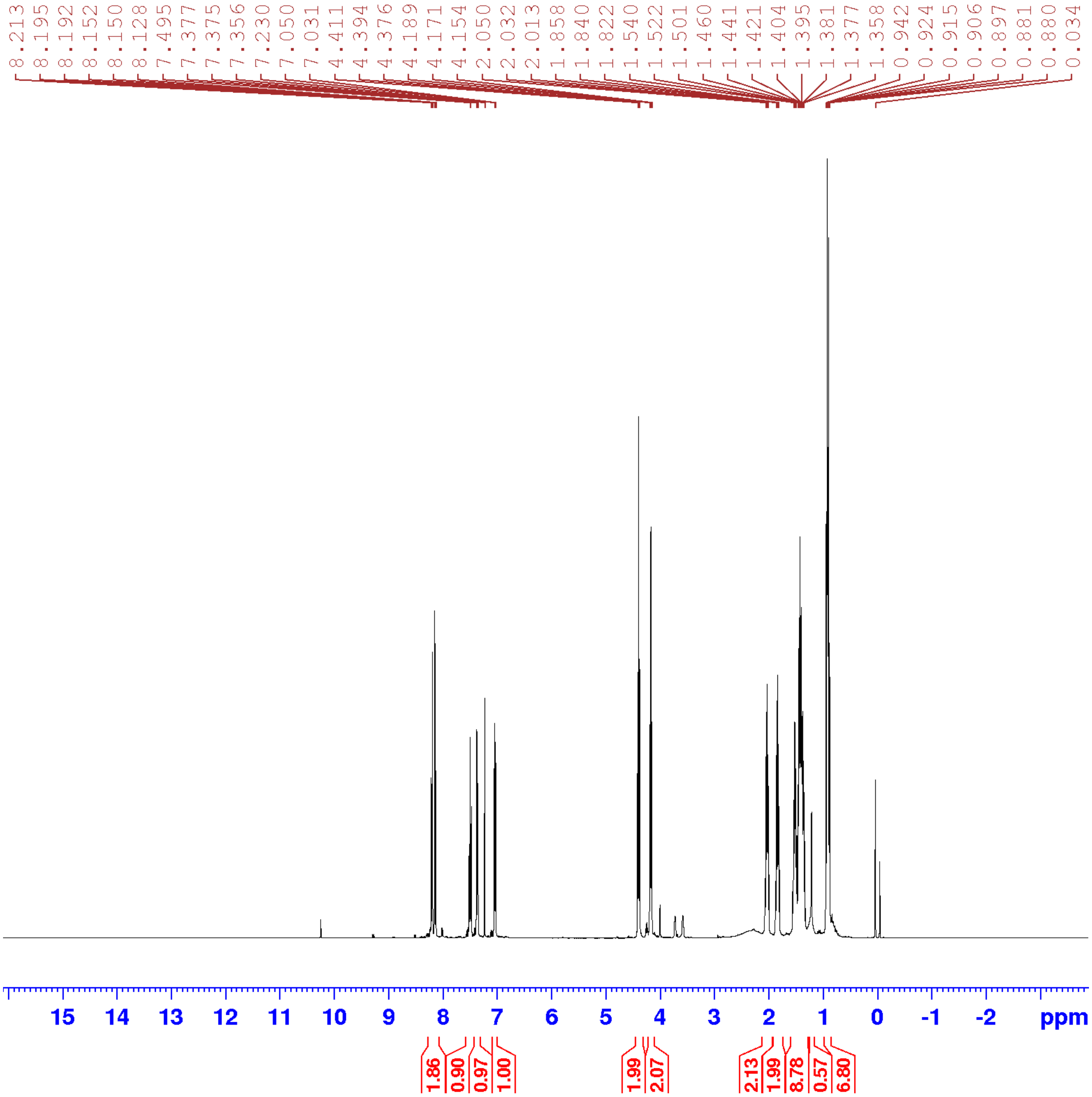

Figure S35: ^{1}H NMR of QD-10

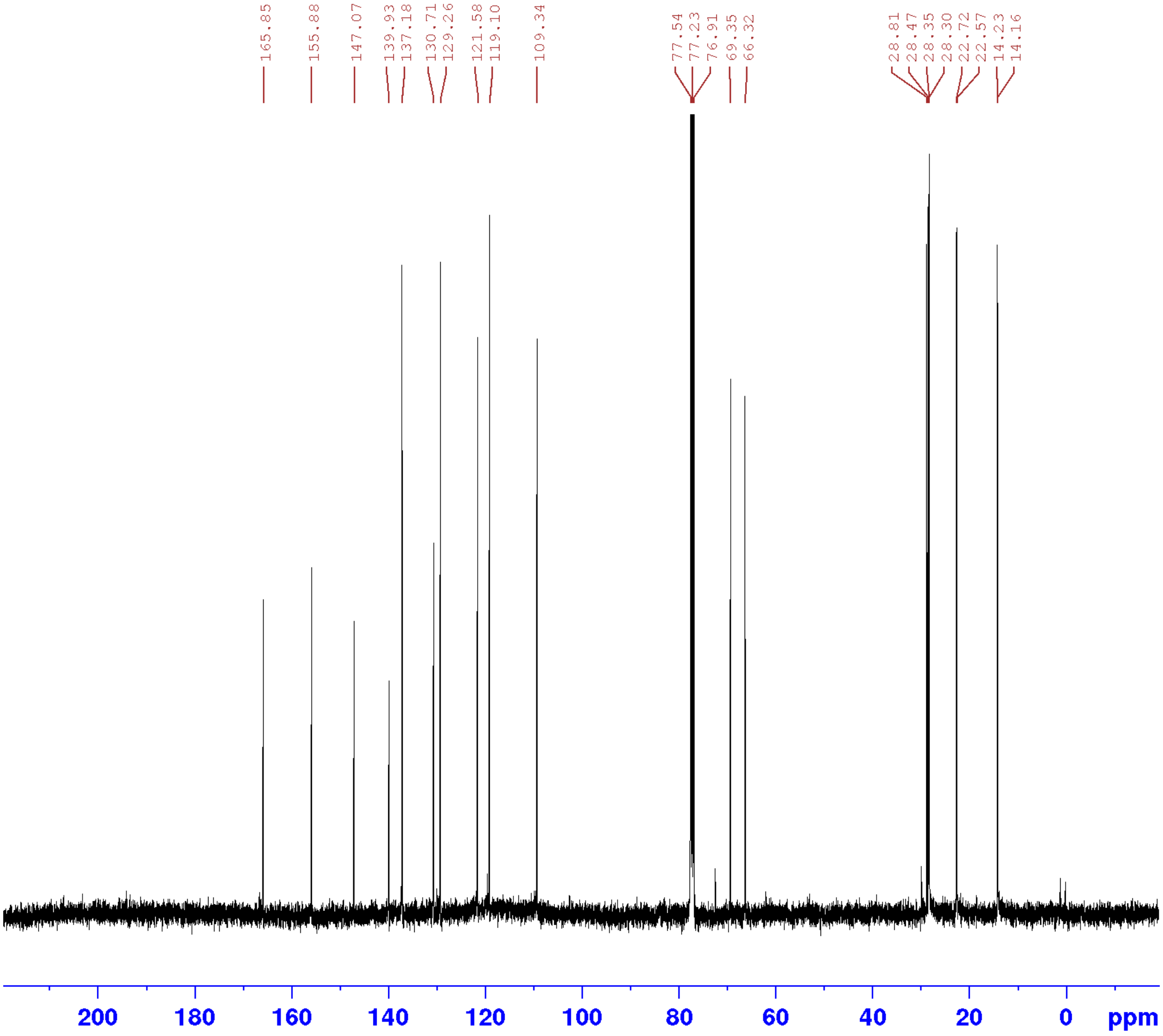

Figure S36: ^{13}C NMR of QD-10

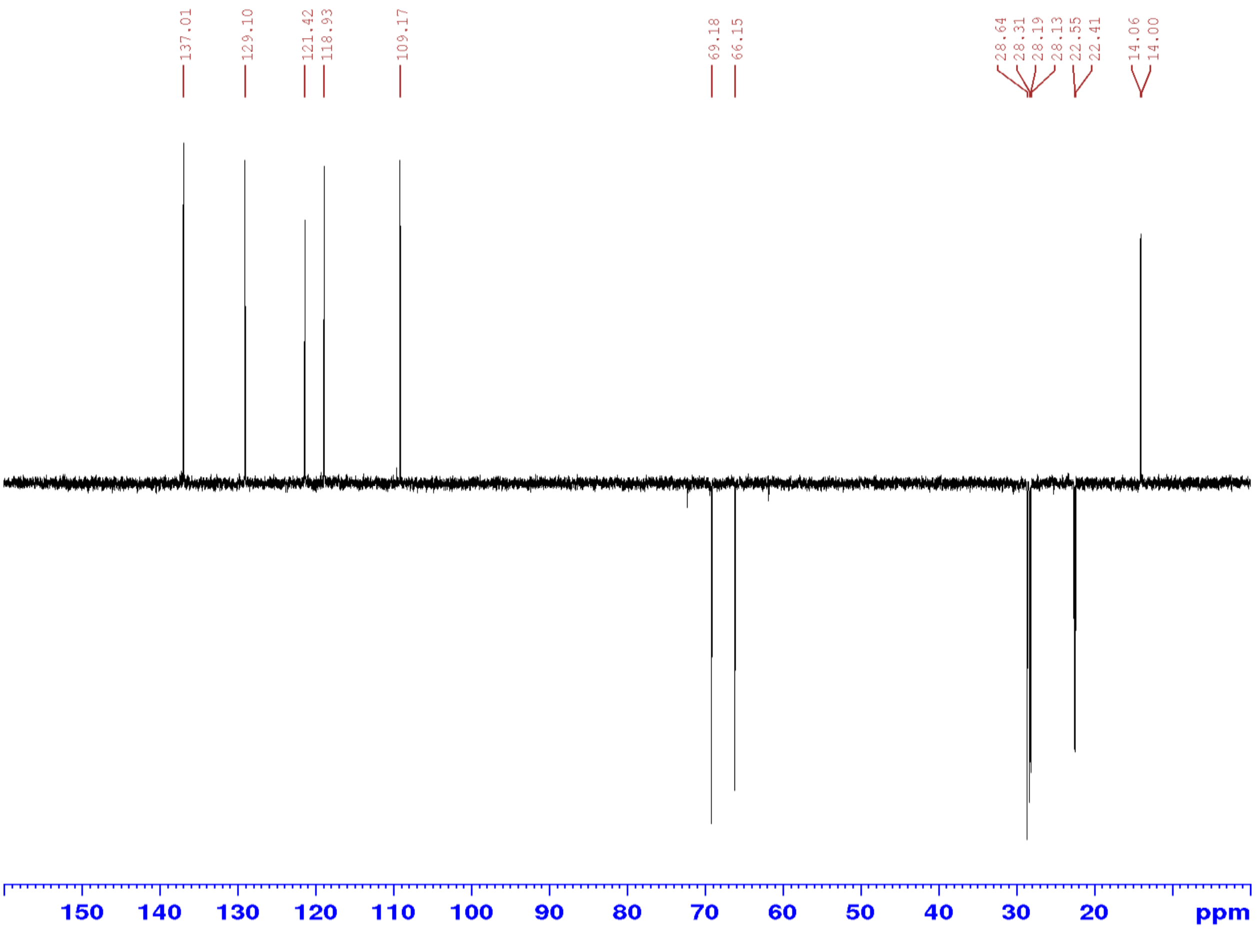

Figure S37: DEPT-135 NMR of QD-10

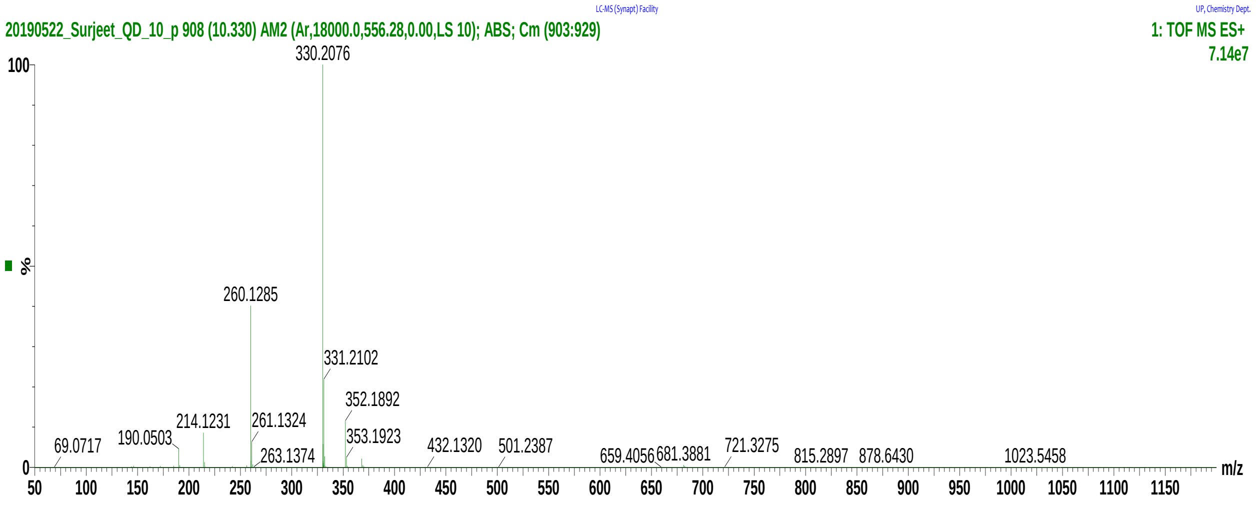

Figure S38: HRESI-MS of QD-10

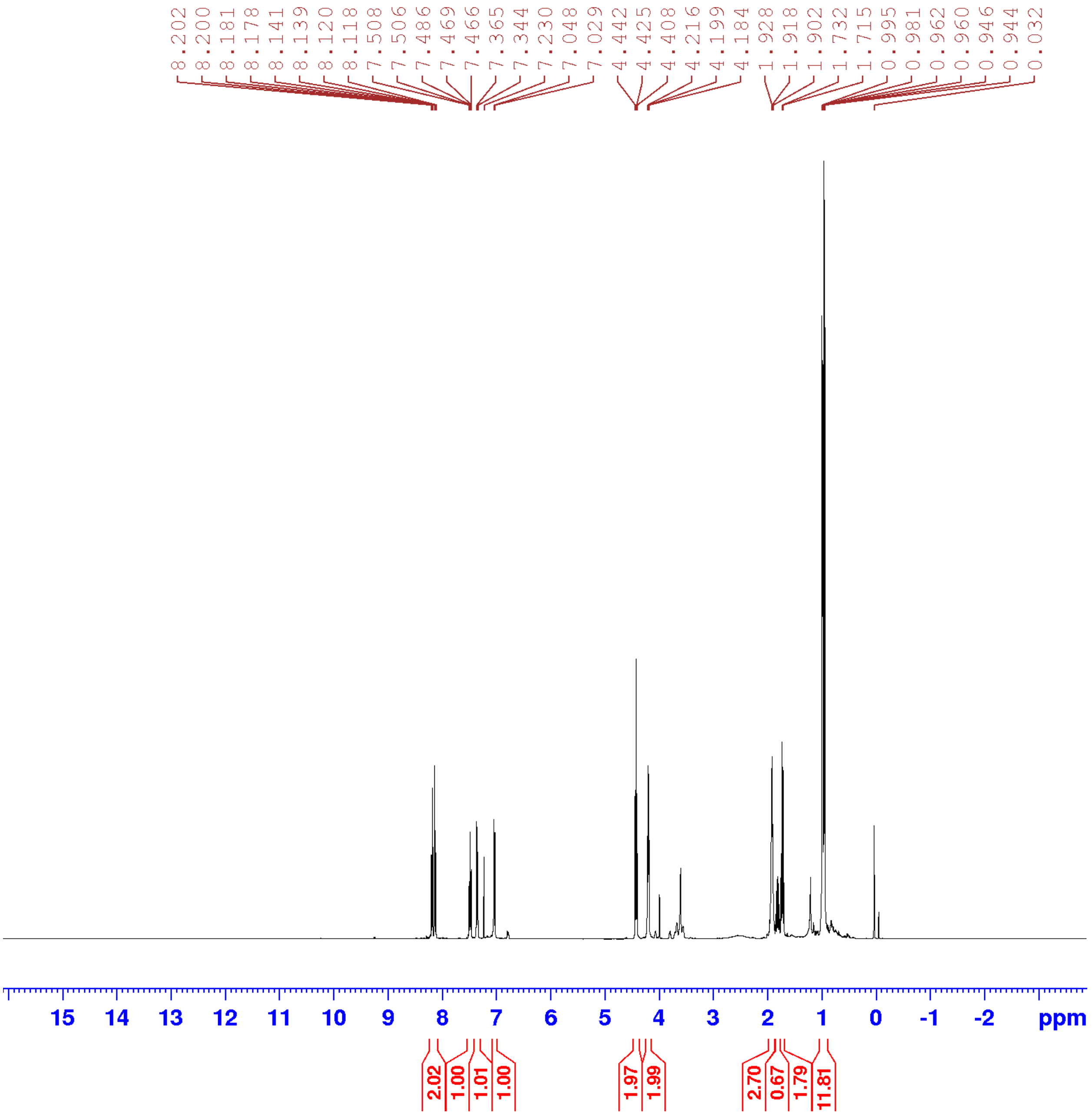

Figure S39: ^{1}H NMR of QD-11

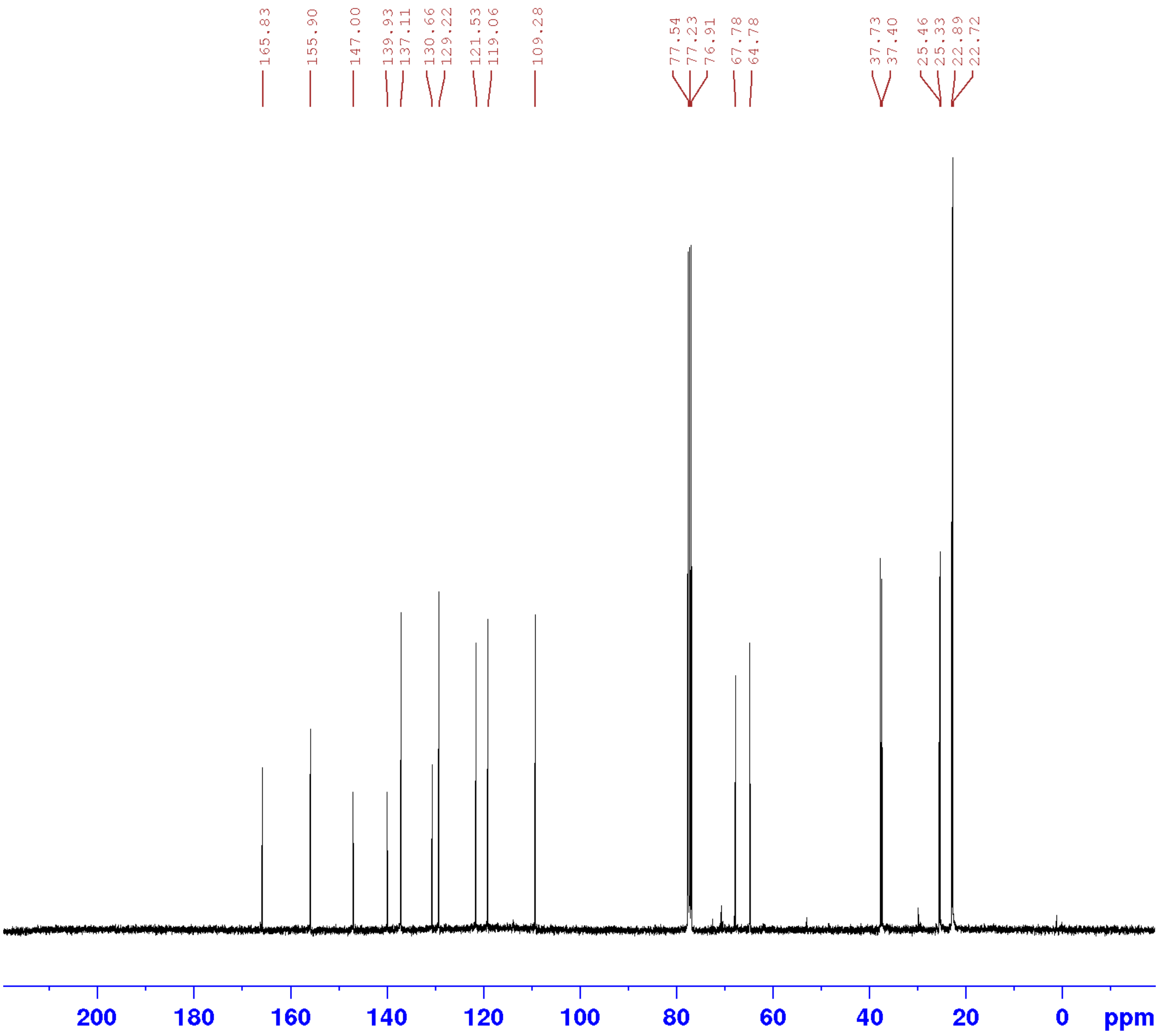

Figure S40: ^{13}C NMR of QD-11

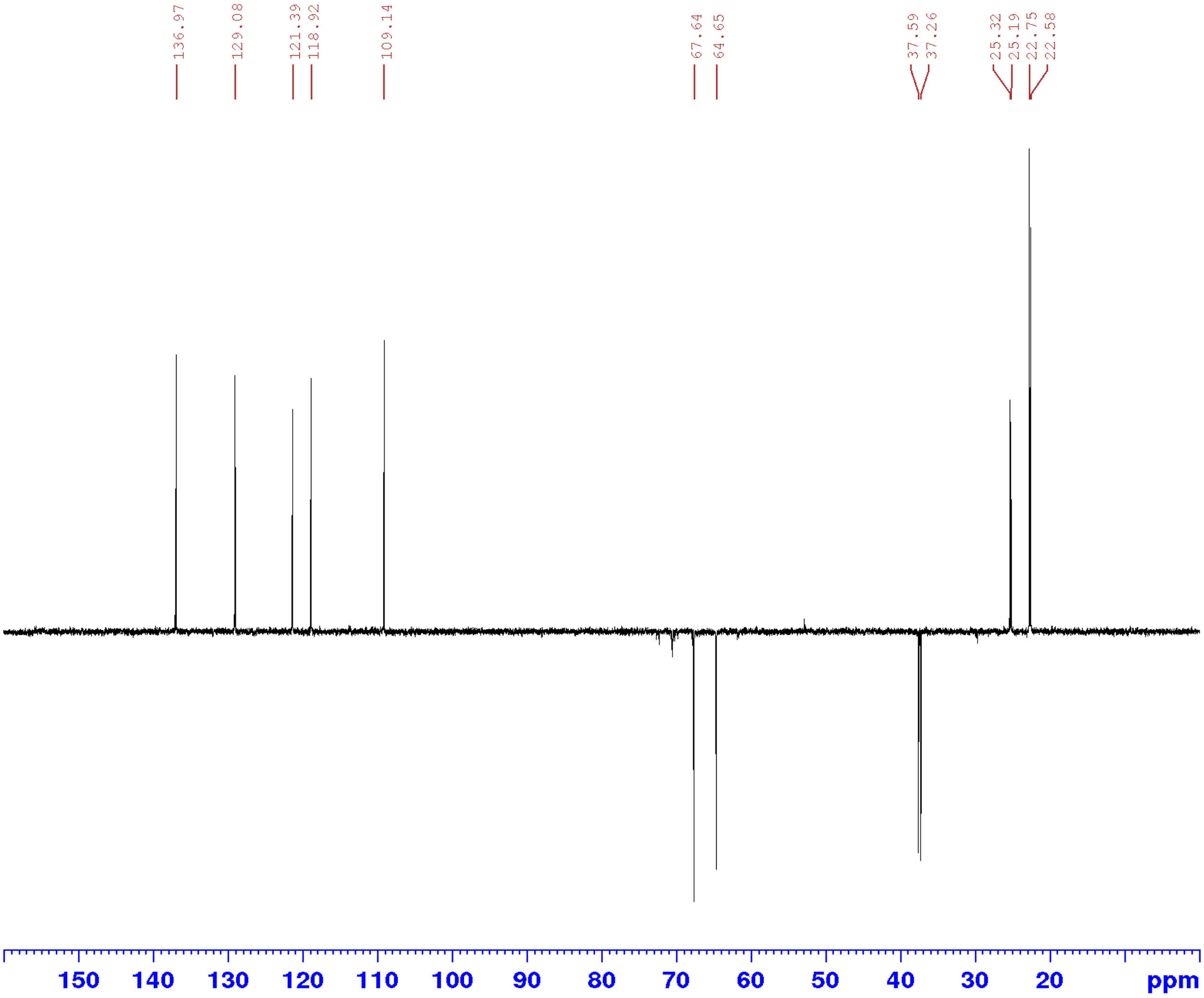

Figure S41: DEPT-135 NMR of QD-11

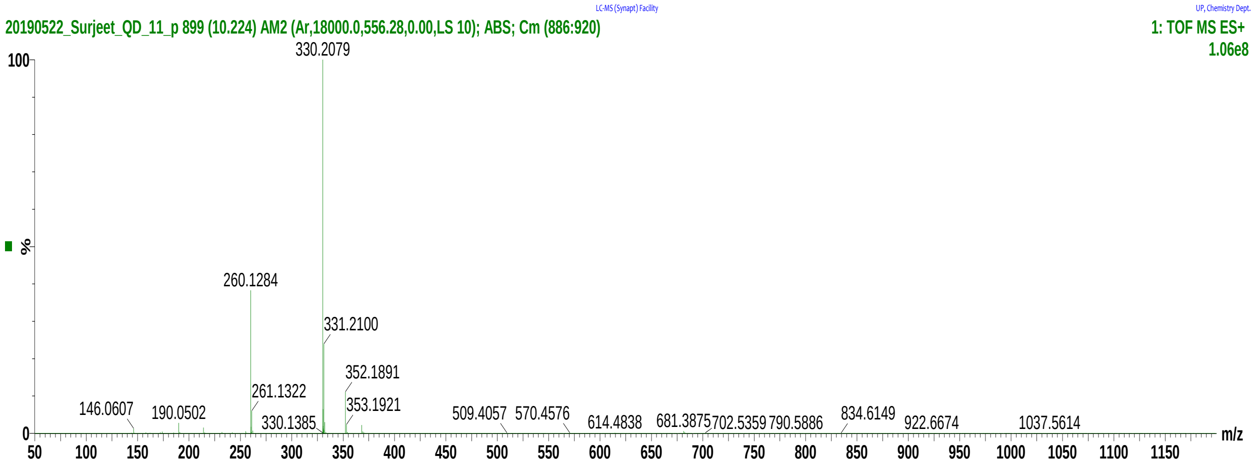

Figure S42: HRESI-MS of QD-11

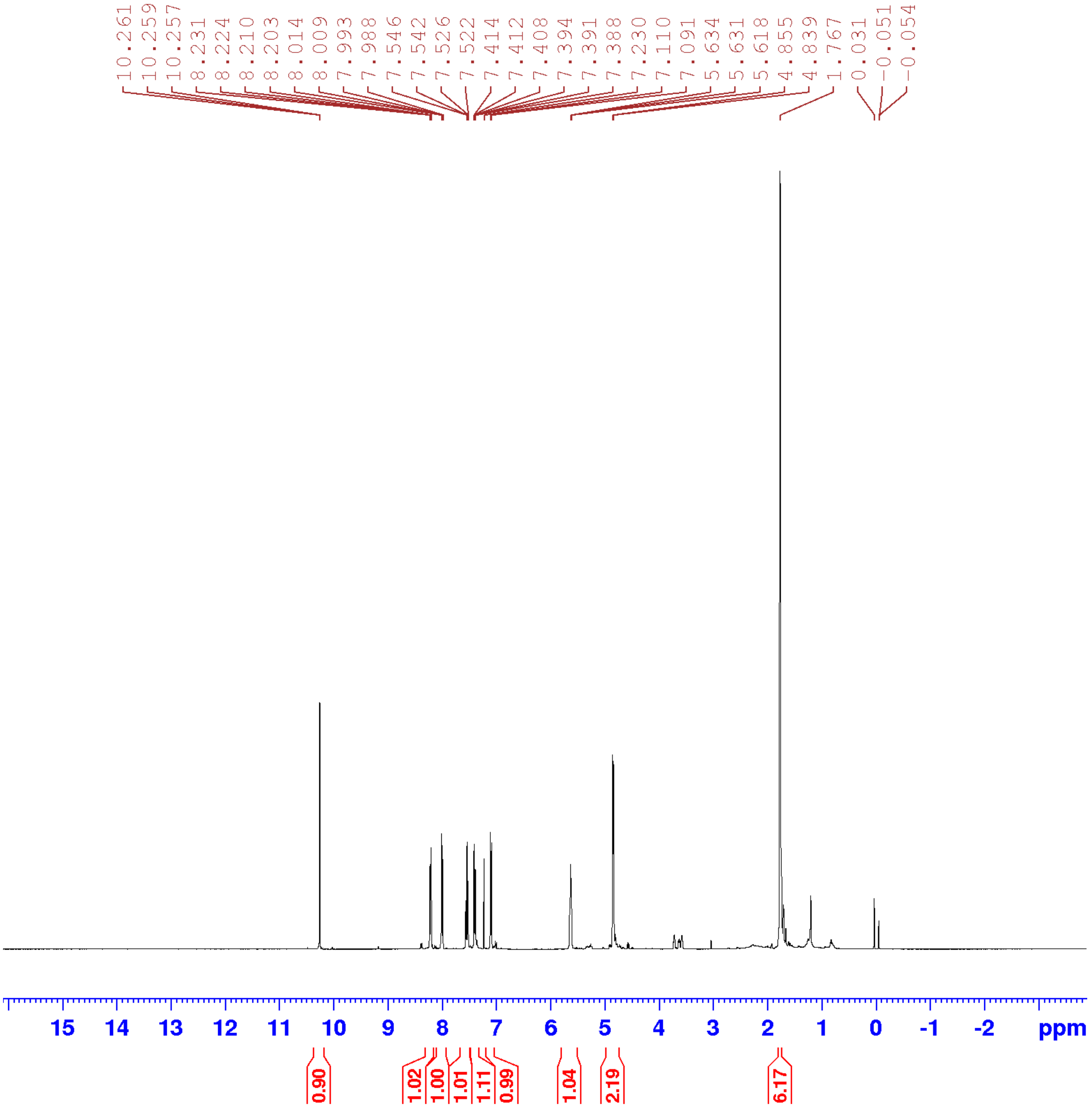

Figure S43: ^{1}H NMR of QD-12

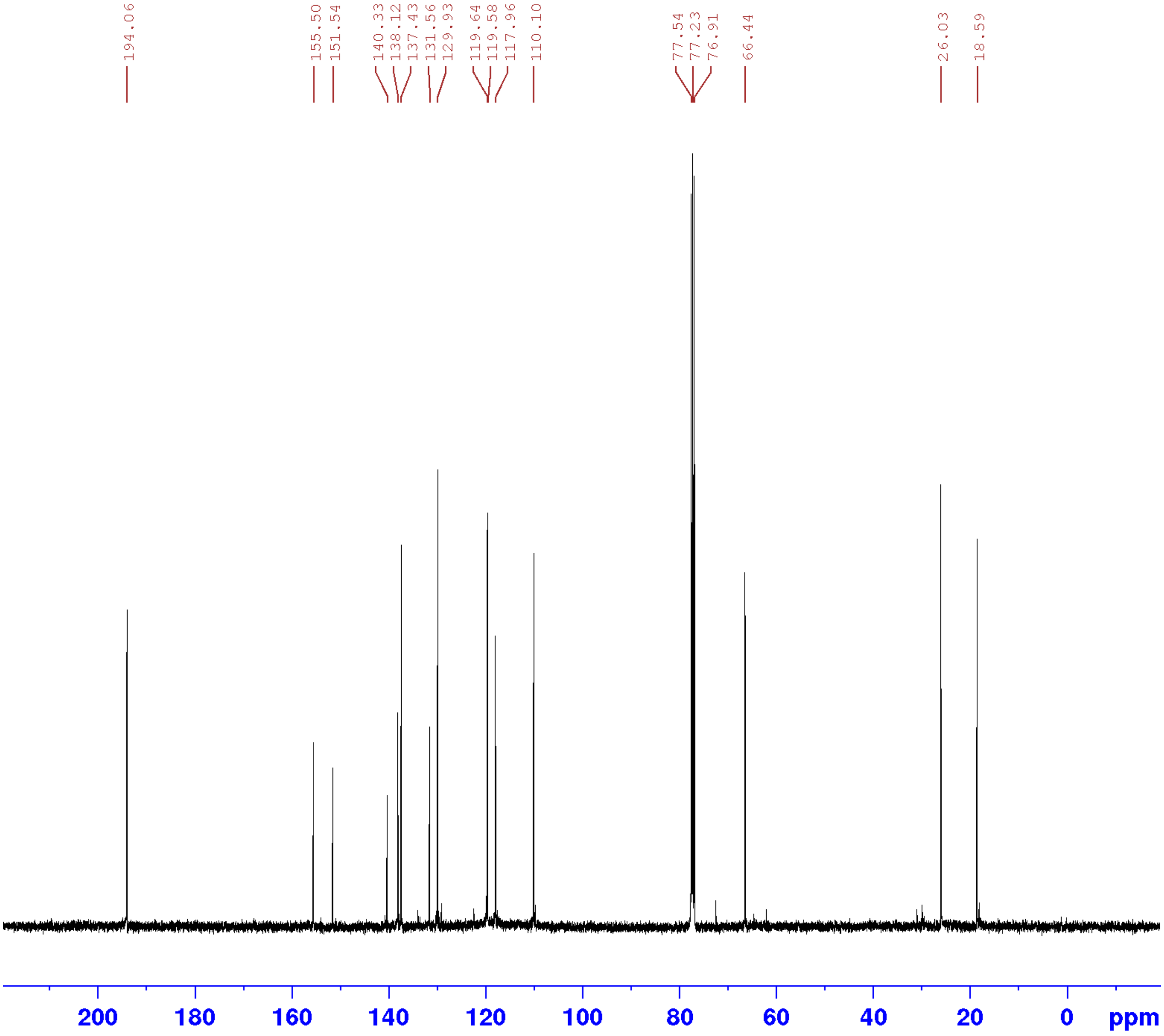

Figure S44: ^{13}C NMR of QD-12

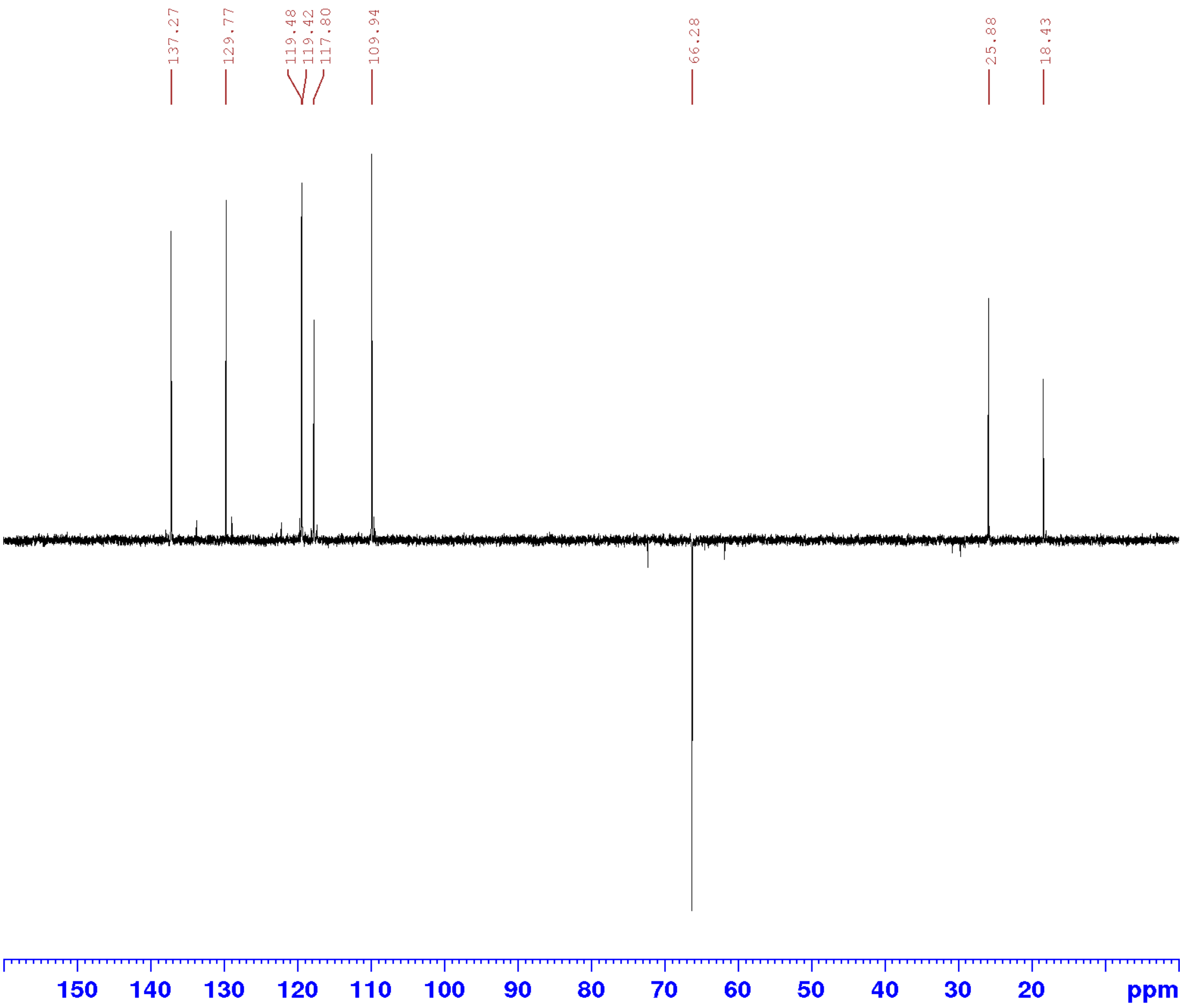

Figure S45: DEPT-135 NMR of QD-12

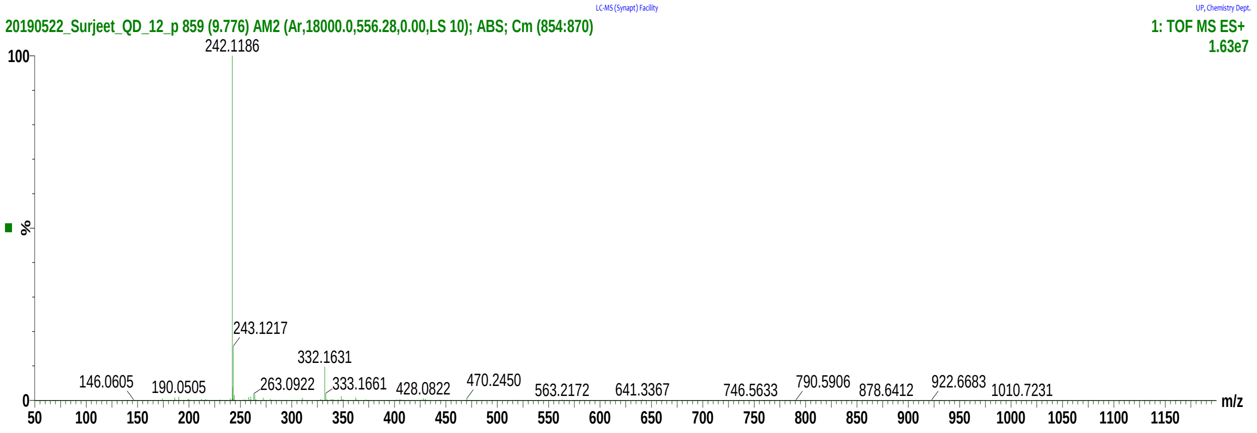

Figure S46: HRESI-MS NMR of QD-12

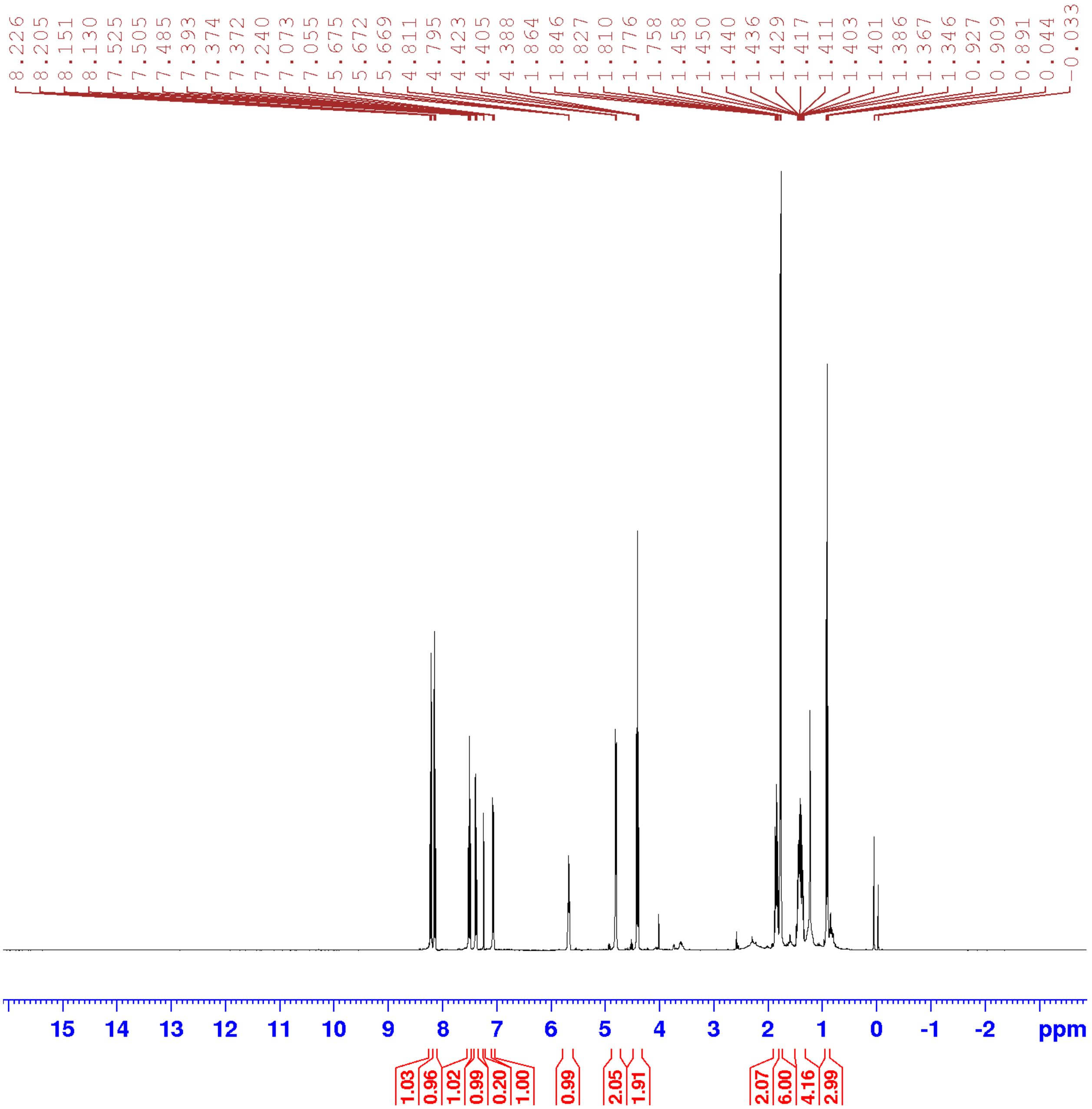

Figure S47: ^{1}H NMR of QD-12a

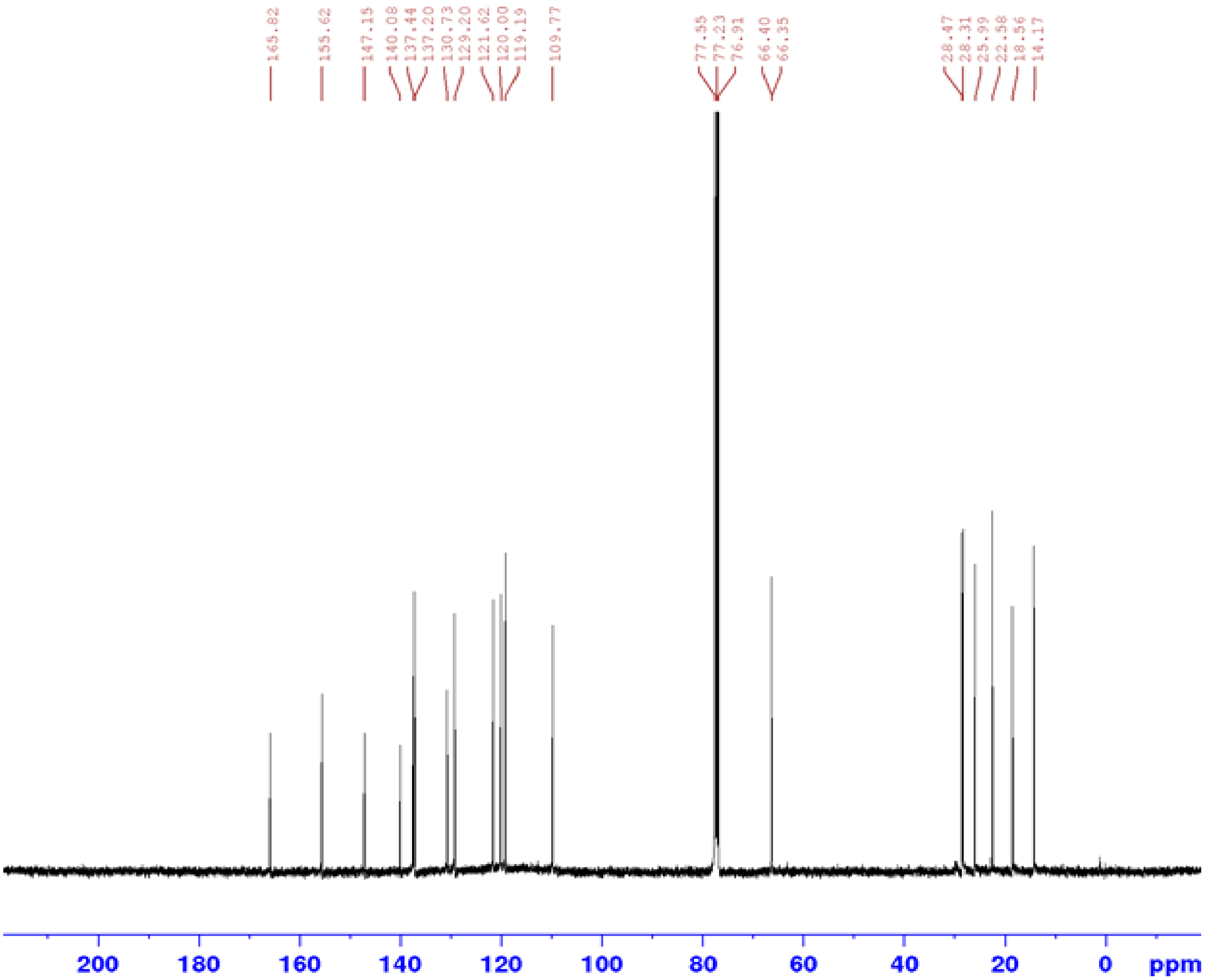

Figure S48: ^{13}C NMR of QD-12a

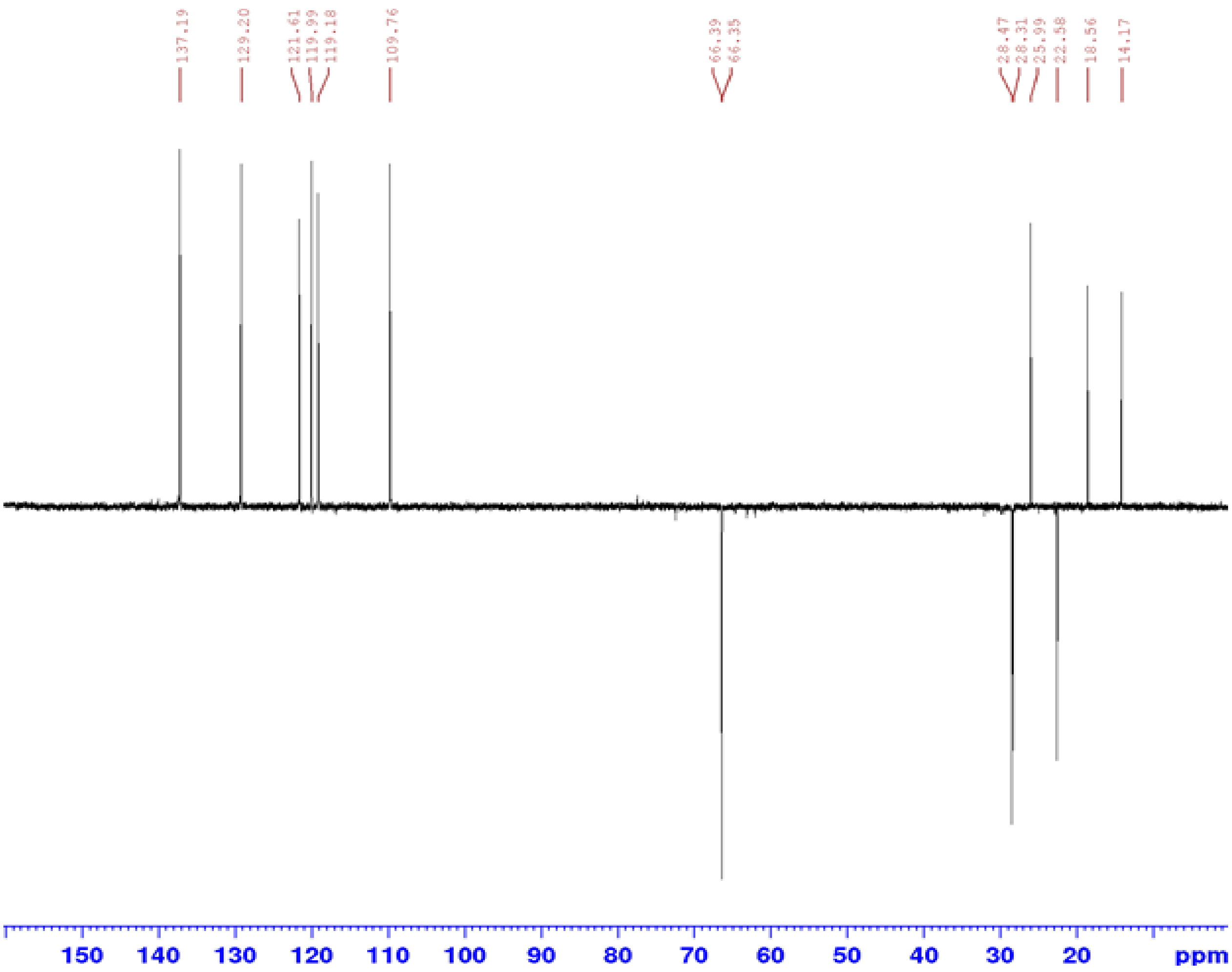

Figure S49: DEPT-135 NMR of QD-12a

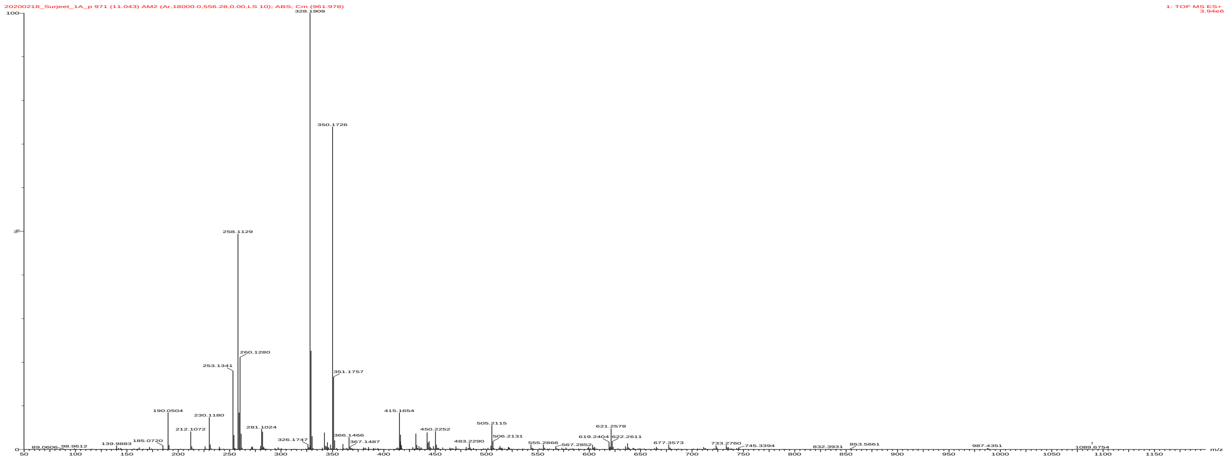

Figure S50: HRESI-MS of QD-12a

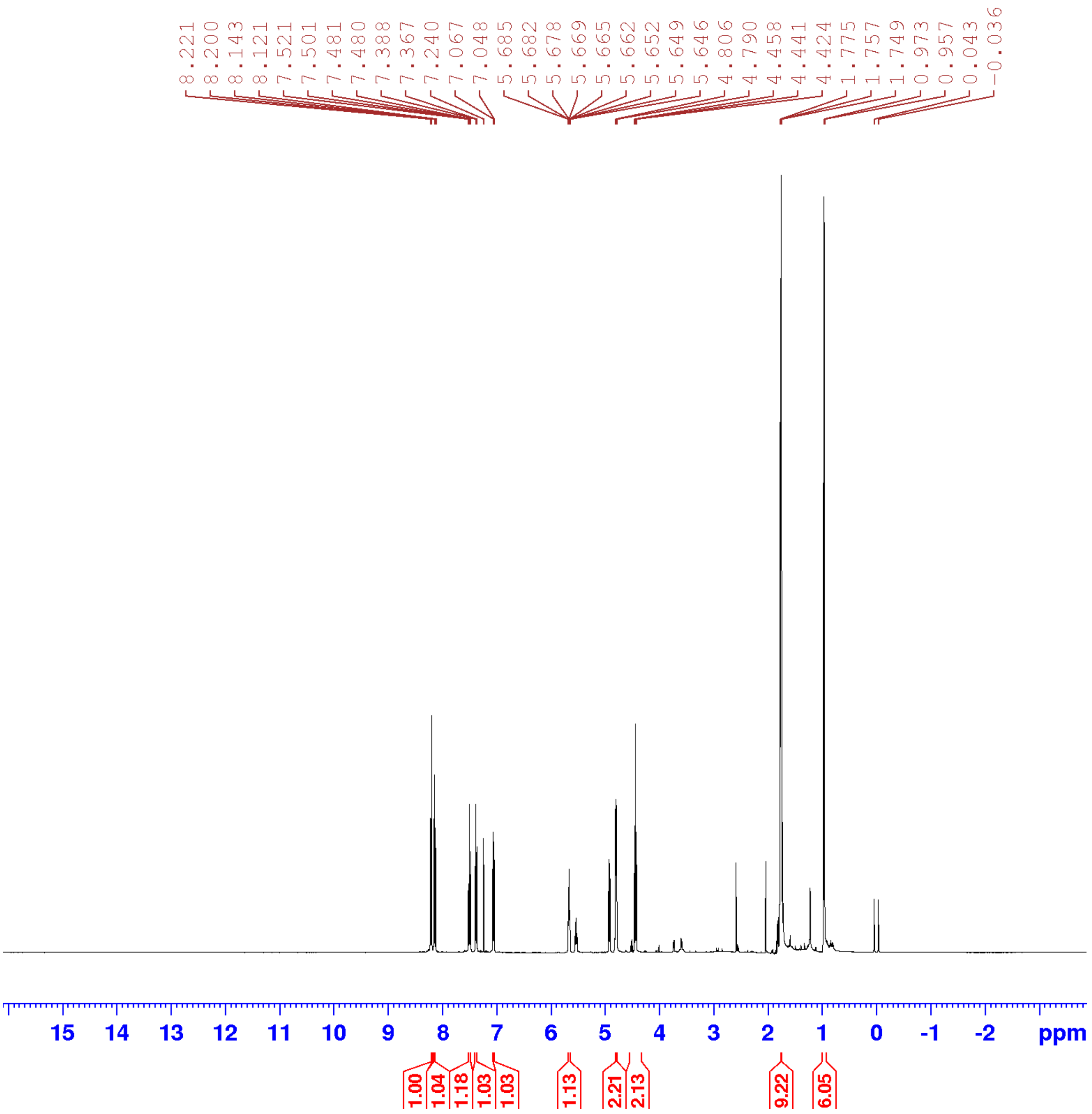

Figure S51: ^{1}H NMR of QD-12b

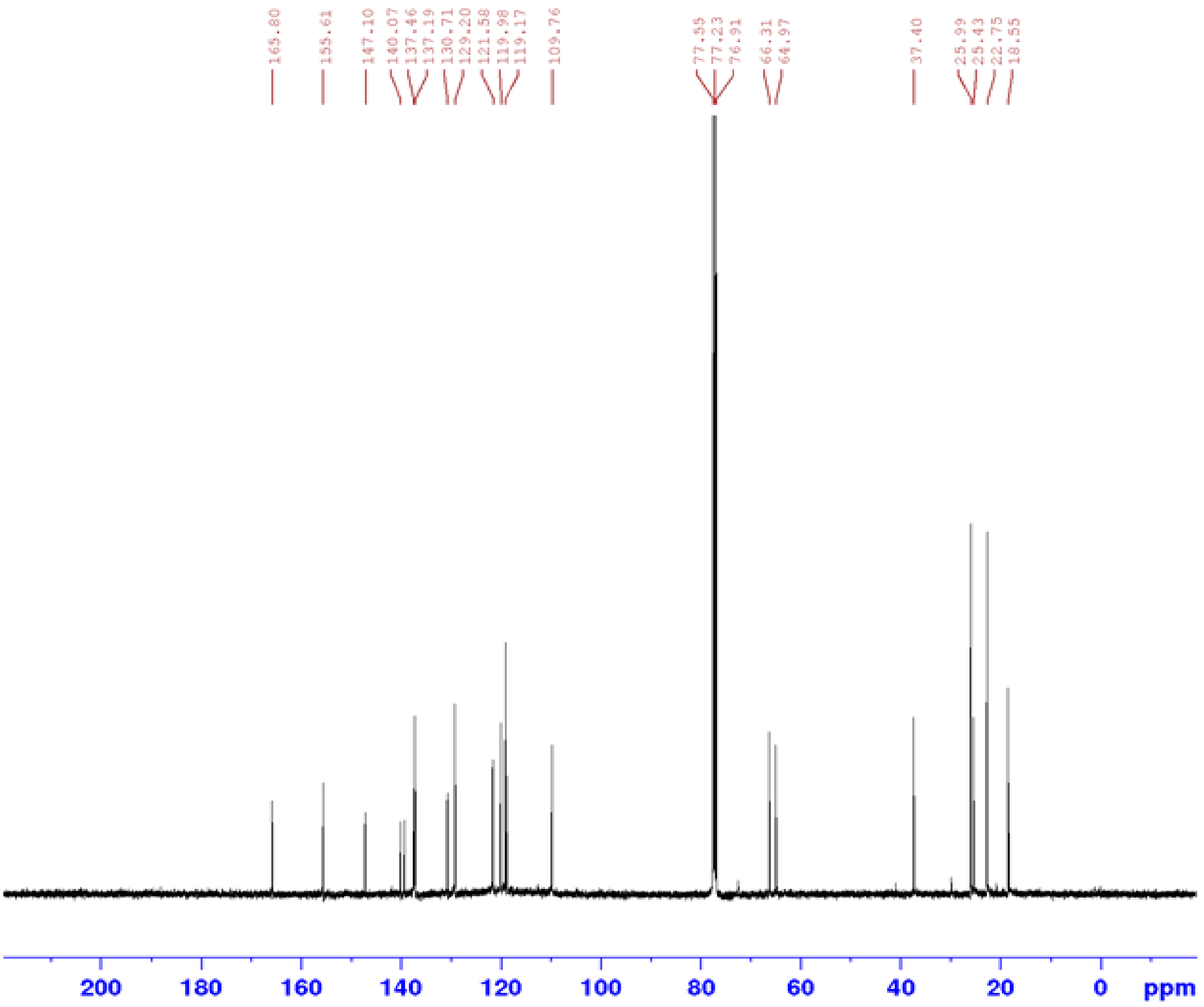

Figure S52: ^{13}C NMR of QD-12b

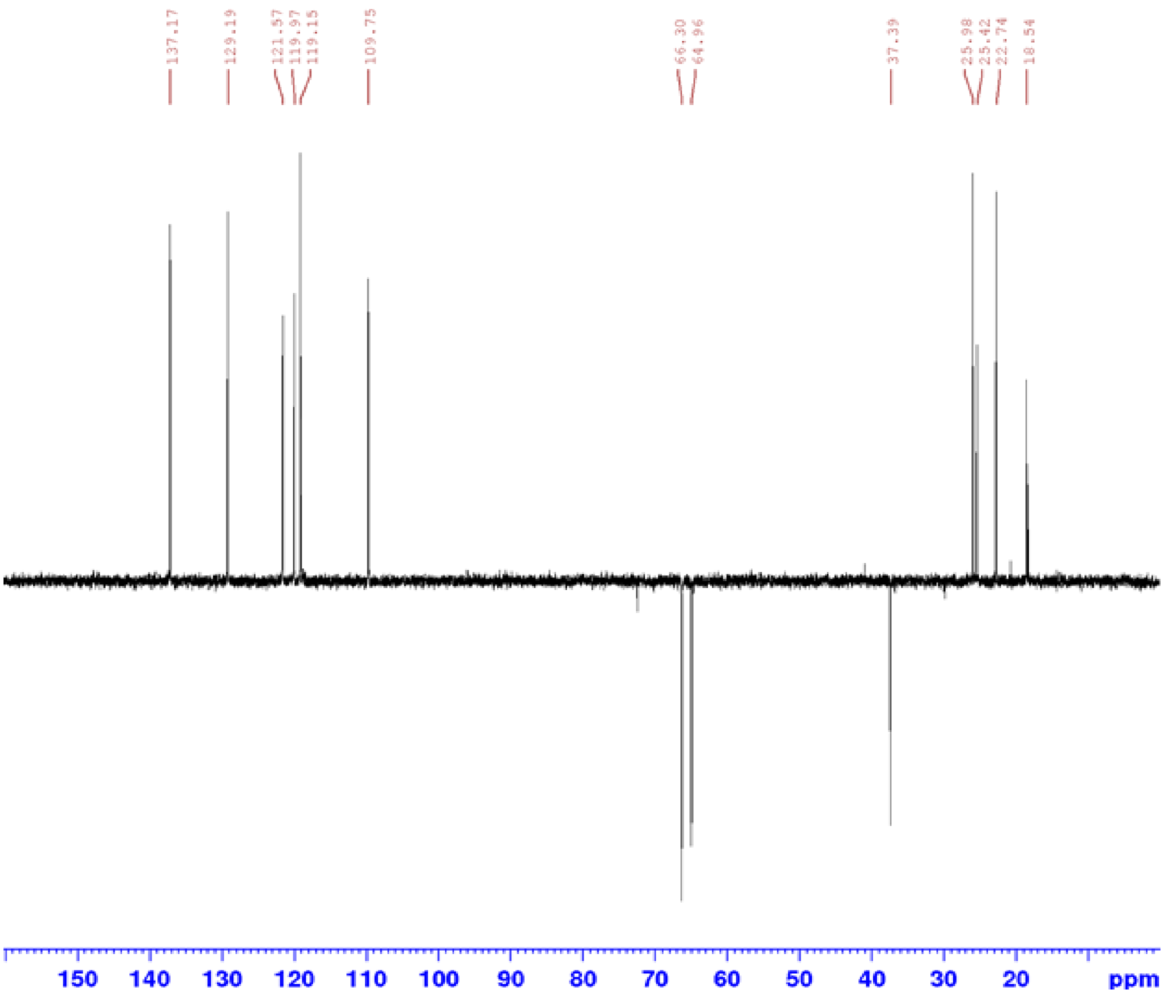

Figure S53: DEPT-135 NMR of QD-12b

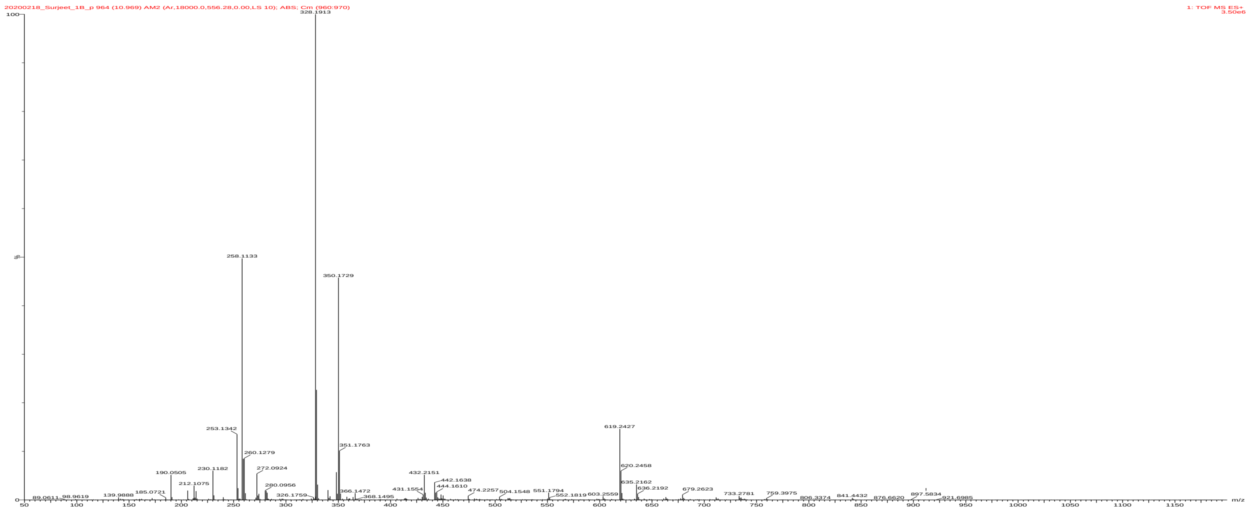

Figure S54: HRESI-MS of QD-12b
